# Supplementary material for: β-Ketophosphonates with Pentalenofuran Scaffolds Linked to the Ketone Group for the Synthesis of Prostaglandin Analogs
Source: Int J Mol Sci. 2021 Jun 24;22(13):6787. doi: 10.3390/ijms22136787 (PMC8268005; doi:10.3390/ijms22136787)
Supplement: Supplementary file 1 [file ijms-22-06787-s001.zip › ijms-1243296-supplementary.pdf]

β-Ketophosphonates with pentalenofurane scaffolds linked to the ketone group for the synthesis of prostaglandin analogues.

Constantin I. Tănase,[1*] Constantin Drăghici,[2] Miron Teodor Căproiu,[2] Anamaria Hanganu,[2] Gheorghe Borodi,[3] Maria Maganu,[2] Emese Gal,[4] Lucia Pintilie[1]

[1] *National Institute for Chemical-Pharmaceutical Research and Development, 112 Vitan Av., 74373, Bucharest 3, ROMANIA, cvtanase@gmail.com, lucia.pintilie@gmail.com*

[2] *Organic Chemistry Center "C.D. Nenitzescu", 202 B, Splaiul Independentei, Bucharest 6, ROMANIA, cst_drag@yahoo.com, dorucaproiu@gmail.com, anamaria_hanganu@yahoo.com, mmaganu@yahoo.com*

[3] *National Institute For R&D of Isotopic and Molecular Technologies, 67-103 Donat, Cluj-Napoca 400293, Romania, borodi@itim-cj.ro*

[4] *Babes-Bolyai University, Faculty of Chemistry and Chemical Engineering, Arany János 11, 400012, Cluj-Napoca, ROMANIA, gal.emese.81@gmail.com*

**Correspondence author: cvtanase@gmail.com*

1. NMR-Spectra of the compounds on Bruker aparatus; for the compound 5a, on a Varian aparatus.

2. IR spectra for the compounds 3b, 3c, 4c, 5b, 5c and 6c.

3. HR-MS spectra for the compounds 5b and 5c.

4. X-ray crystallography for the compounds 6b and 6c.

1. NMR-Spectra of the compounds on Bruker *a)* and on Varian *b)* aparatus.

1.1.^{1}H, ^{13}C, COSY and HETCOR-NMR spectra in $CDCl_3$ of the compound **3a**

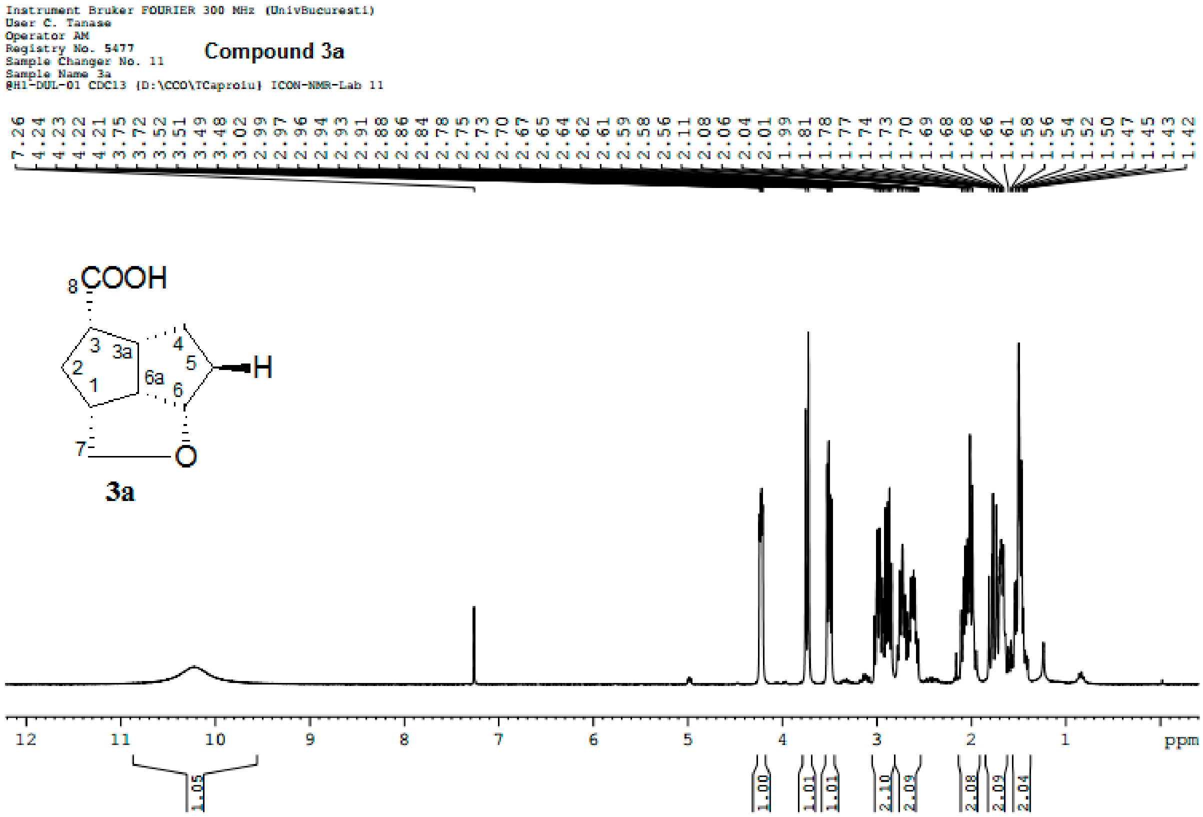

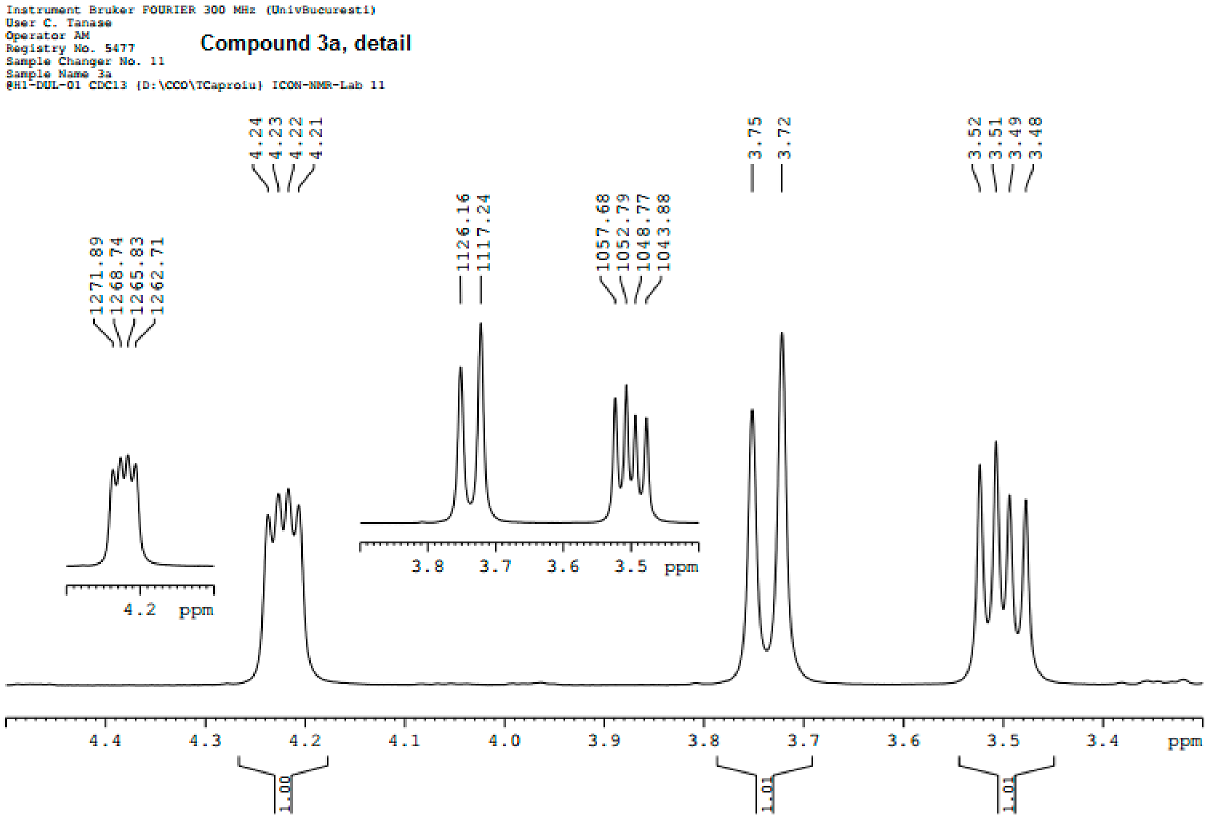

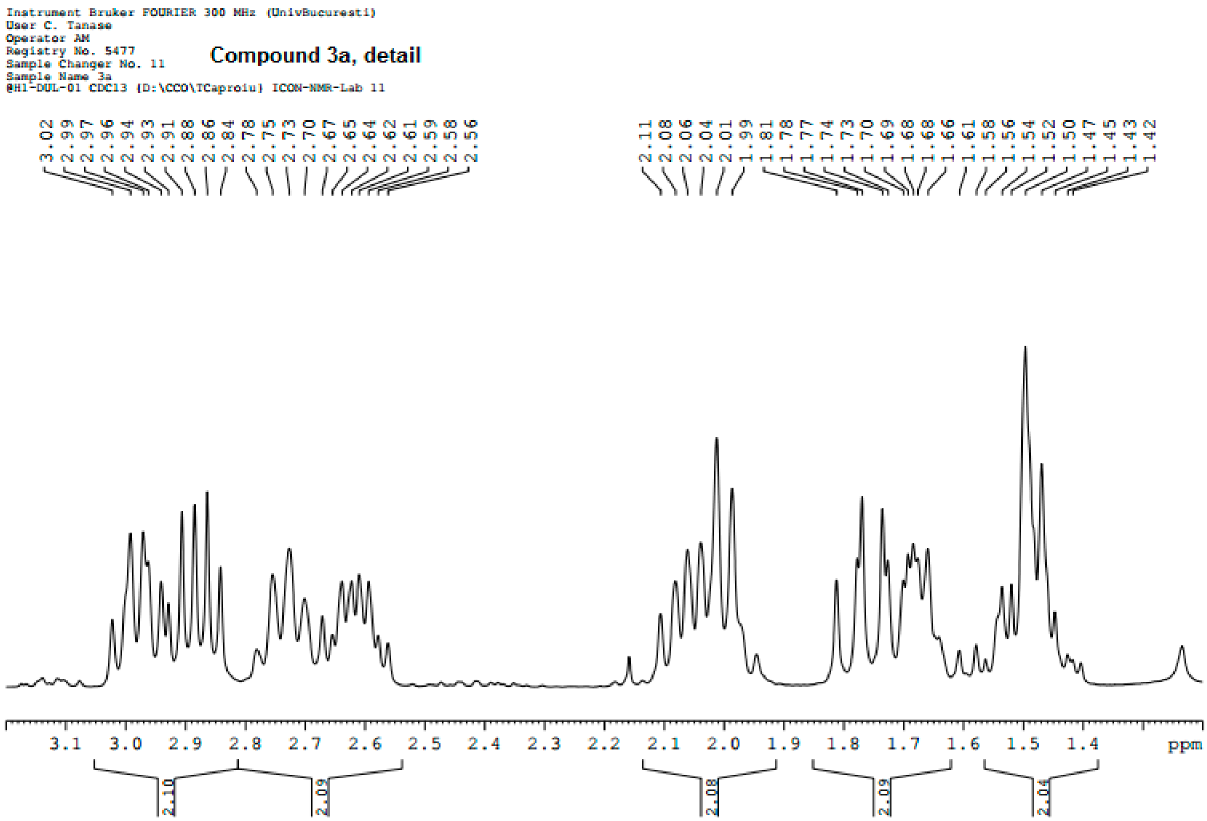
Instrument Bruker FOURIER 300 MHz (UnivBucuresti)
User C. Tanase
Operator AM
Registry No. 5477
Sample Changer No. 11
Sample Name 3a
@H1-DUL-01 CDCl3 (D:\CCO\TCaproiu) ICON-NMR-Lab 11
Compound 3a, detail
3.02 2.99 2.97 2.96 2.94 2.93 2.91 2.88 2.86 2.84 2.78 2.75 2.73 2.70 2.67 2.65 2.64 2.62 2.61 2.59 2.58 2.56
2.11 2.08 2.06 2.04 2.01 1.99 1.81 1.78 1.77 1.74 1.73 1.70 1.69 1.68 1.68 1.66 1.61 1.58 1.56 1.54 1.52 1.50 1.47 1.45 1.43 1.42
2.10 2.09 2.08 2.09 2.04
ppm

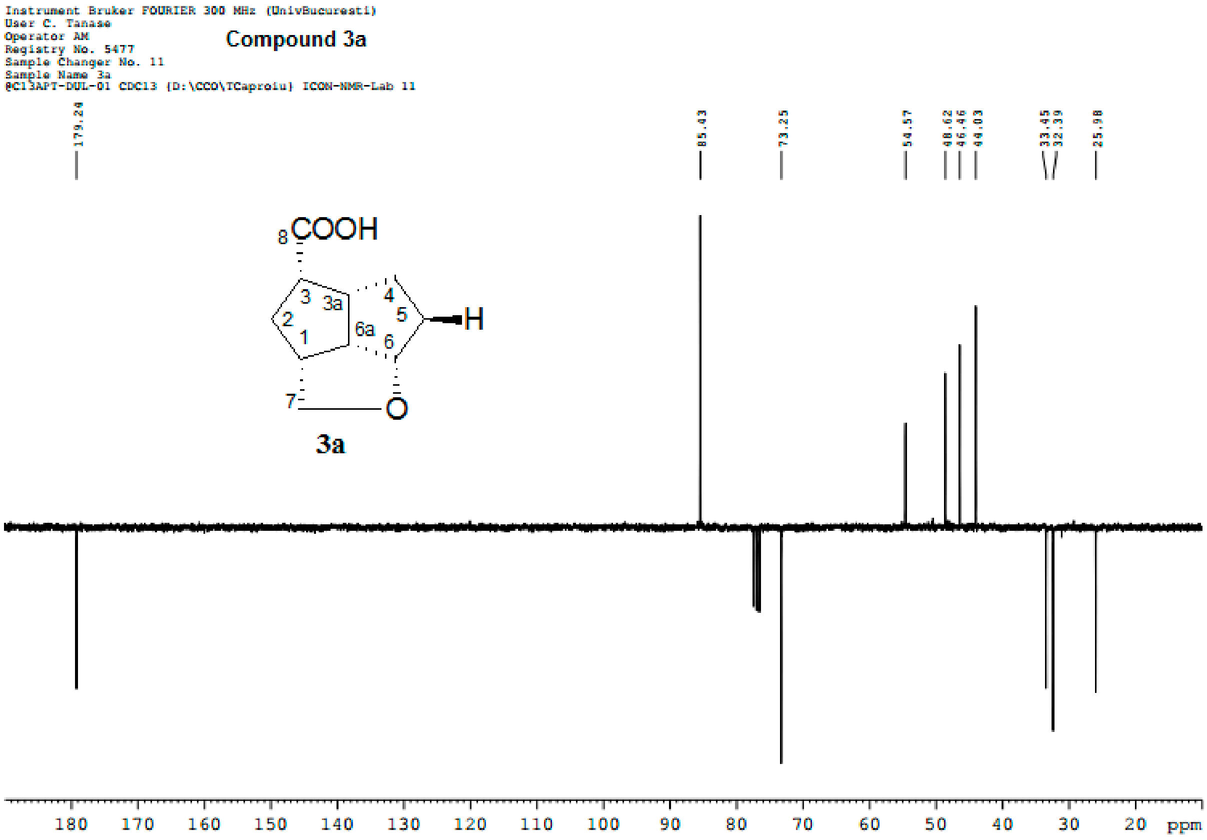
Instrument Bruker FOURIER 300 MHz (UnivBucuresti)
User C. Tanase
Operator AM
Registry No. 5477
Sample Changer No. 11
Sample Name 3a
@C13APT-DUL-01 CDCl3 (D:\CCO\TCaproiu) ICON-NMR-Lab 11
Compound 3a
179.24
85.43
73.25
54.57
48.62
46.46
44.03
33.45
32.39
25.98
8 COOH
3 3a 4 5 H 2 1 6a 6 7 O
3a
ppm

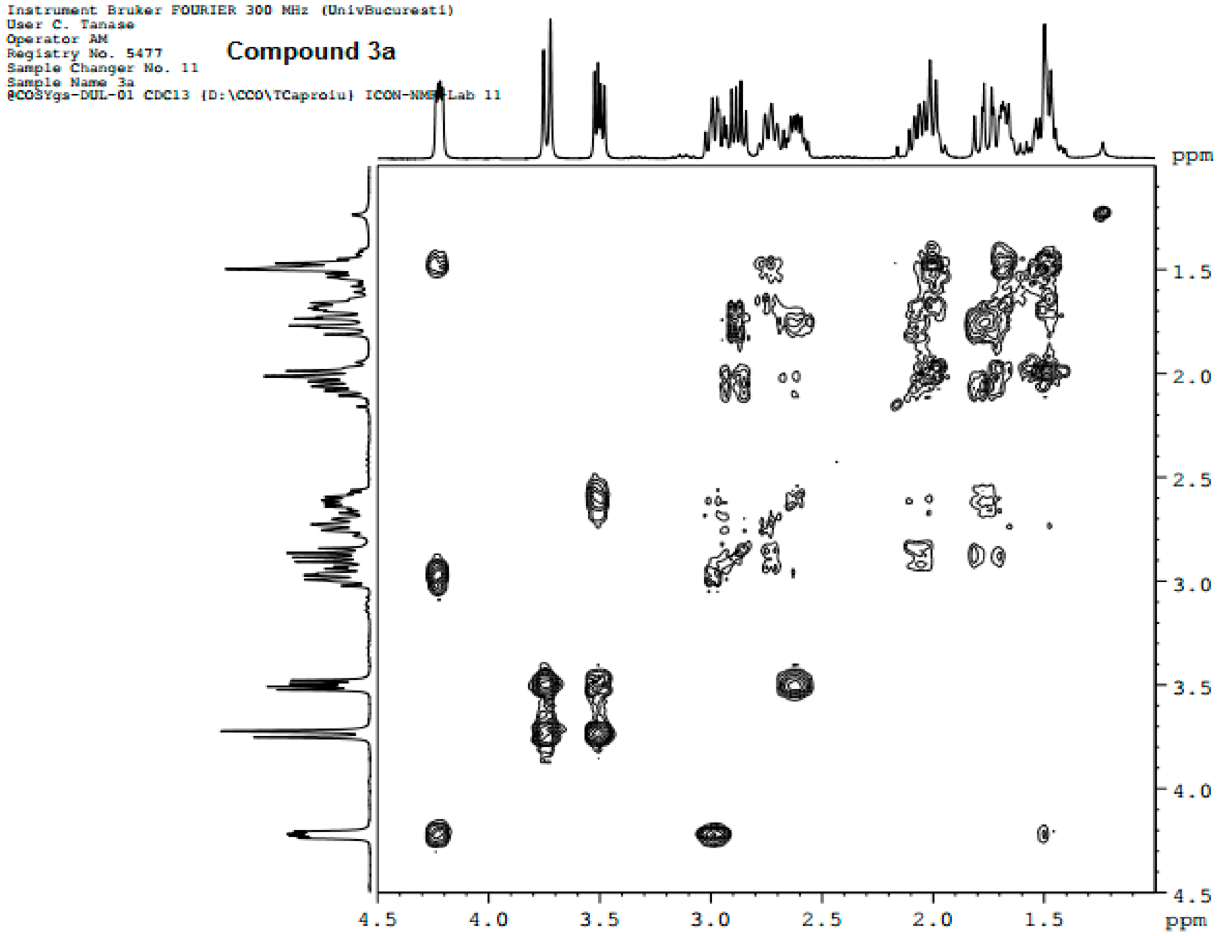
Instrument Bruker FOURIER 300 MHz (UnivBucuresti)
User C. Tanase
Operator AM
Registry No. 5477
Sample Changer No. 11
Sample Name 3a
@COSYgs-DUL-01 CDC13 (D:\CCO\TCaproiu) ICON-NMR-Lab 11
Compound 3a
ppm
1.5
2.0
2.5
3.0
3.5
4.0
4.5
4.5 4.0 3.5 3.0 2.5 2.0 1.5
ppm

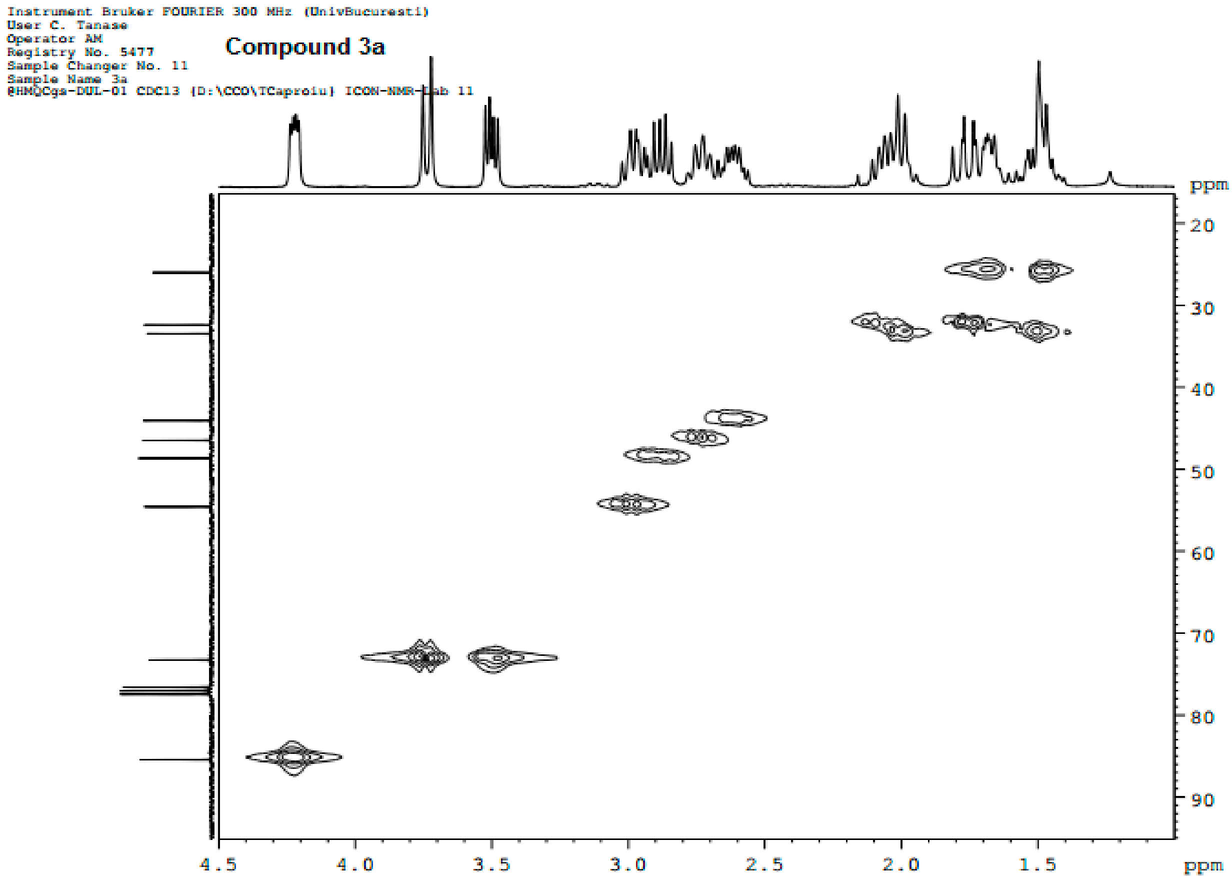
Instrument Bruker FOURIER 300 MHz (UnivBucuresti)
User C. Tanase
Operator AM
Registry No. 5477
Sample Changer No. 11
Sample Name 3a
@HMQCgs-DUL-01 CDC13 (D:\CCO\TCaproiu) ICON-NMR-Lab 11
Compound 3a
ppm
20
30
40
50
60
70
80
90
4.5 4.0 3.5 3.0 2.5 2.0 1.5
ppm

1.2. ^{1}H, ^{13}C, COSY and HETCOR-NMR spectra in $CDCl_3$ of the compound **3b**.

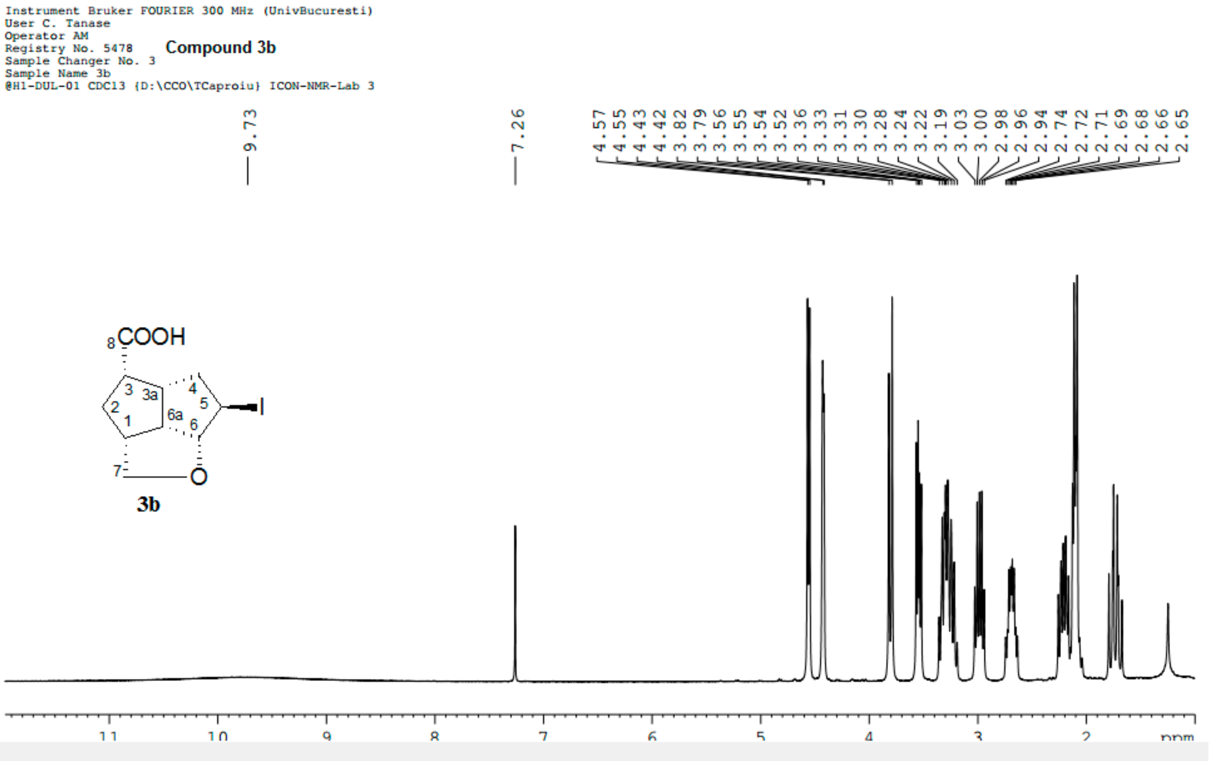

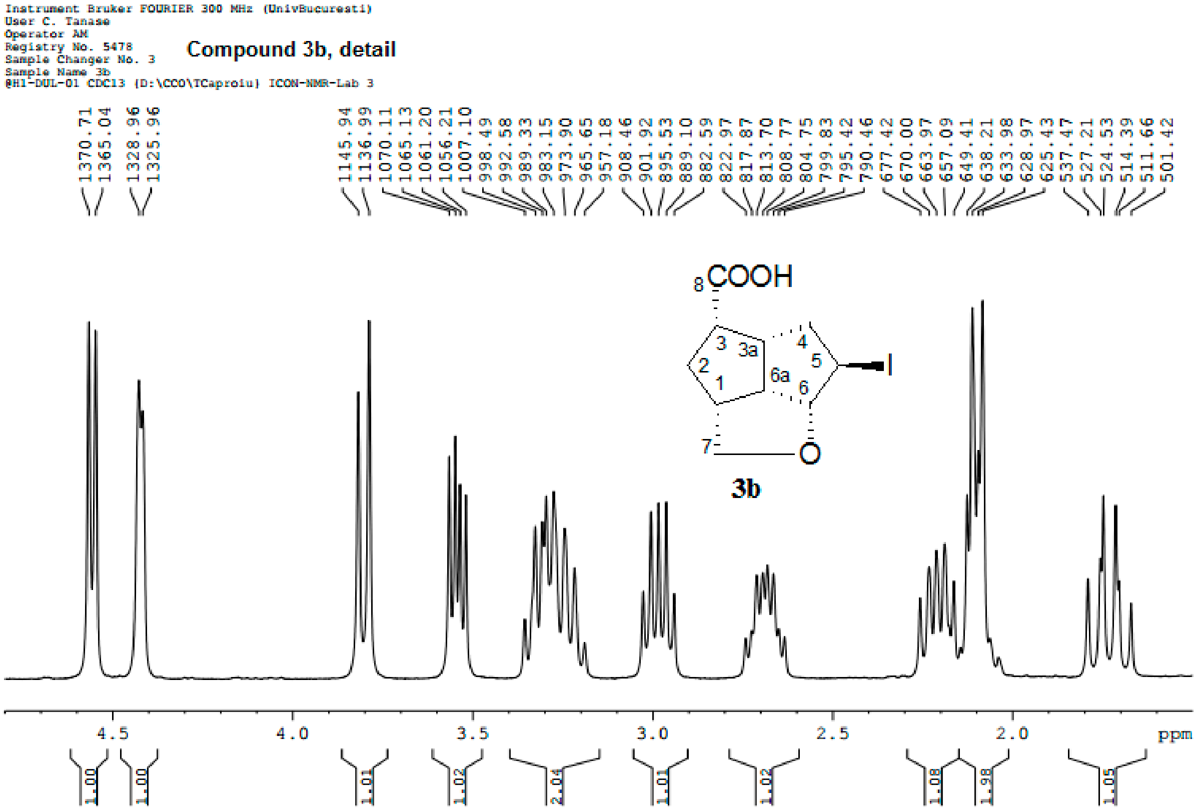

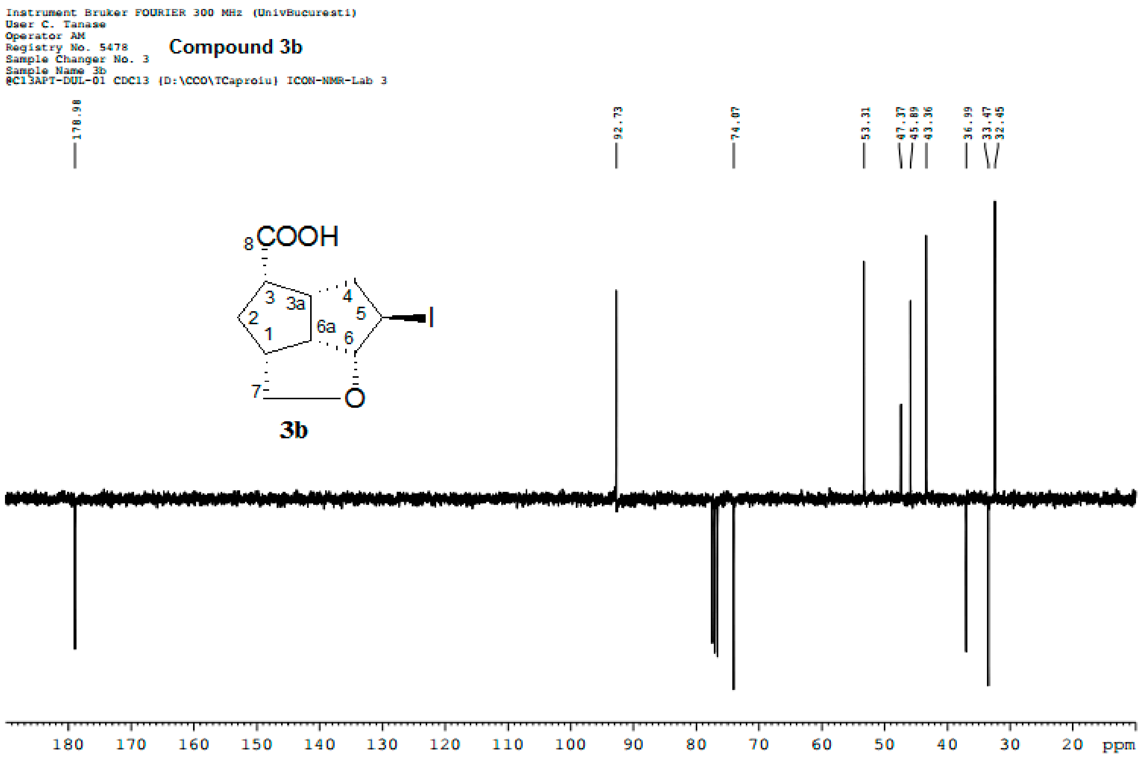
Instrument Bruker FOURIER 300 MHz (UnivBucuresti)
User C. Tanase
Operator AM
Registry No. 5478
Sample Changer No. 3
Sample Name 3b
@C13APT-DUL-01 CDC13 (D:\CCO\TCaproiu) ICON-NMR-Lab 3
Compound 3b
178.98
92.73
74.07
53.31
47.37
45.89
43.36
36.99
33.47
32.45
8
COOH
3
3a
4
5
2
6a
1
6
I
7
O
3b
180 170 160 150 140 130 120 110 100 90 80 70 60 50 40 30 20 ppm

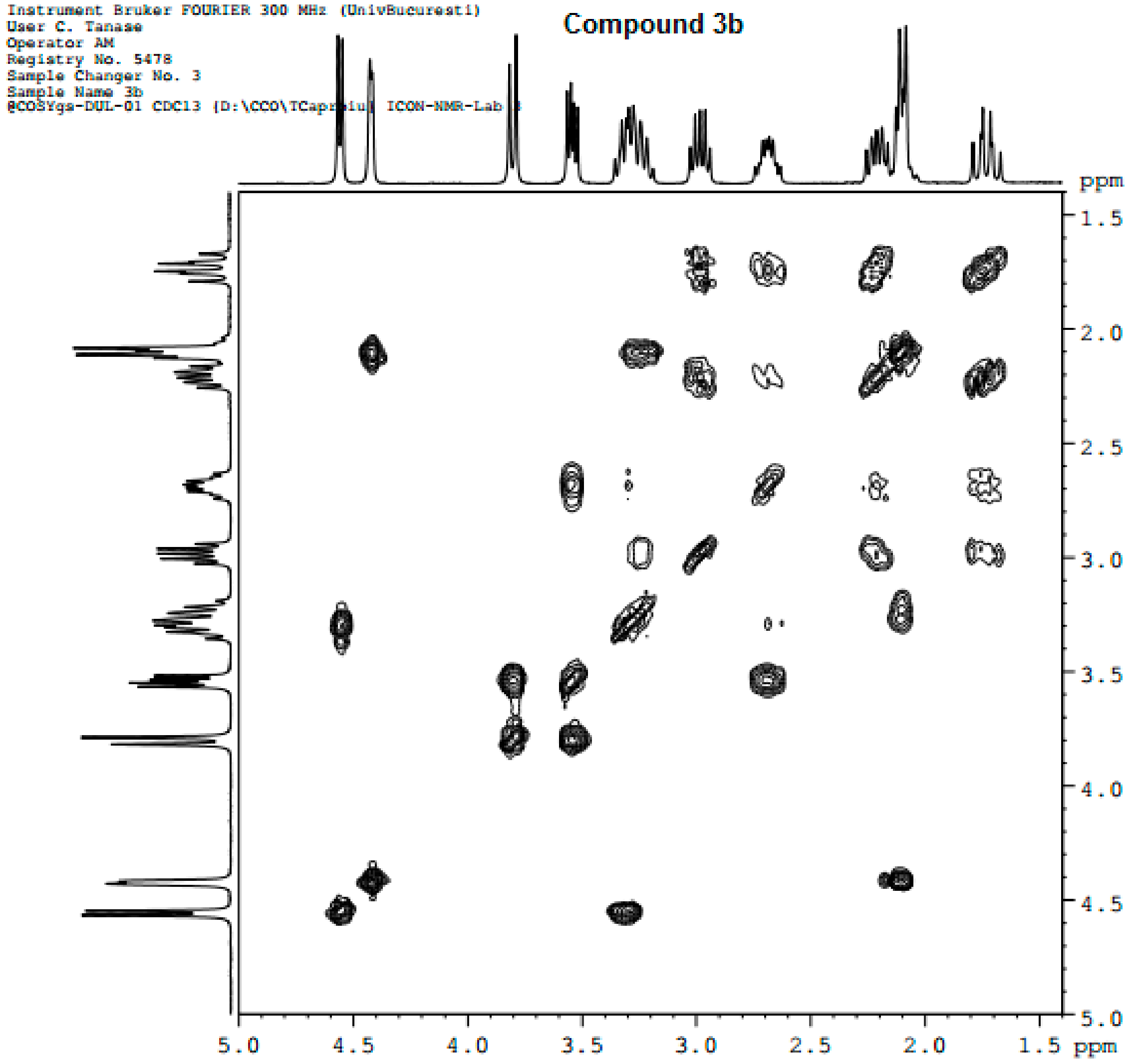
Instrument Bruker FOURIER 300 MHz (UnivBucuresti)
User C. Tanase
Operator AM
Registry No. 5478
Sample Changer No. 3
Sample Name 3b
@COSYgs-DUL-01 CDC13 (D:\CCO\TCaproiu) ICON-NMR-Lab 3
Compound 3b
ppm
1.5
2.0
2.5
3.0
3.5
4.0
4.5
5.0
5.0 4.5 4.0 3.5 3.0 2.5 2.0 1.5 ppm

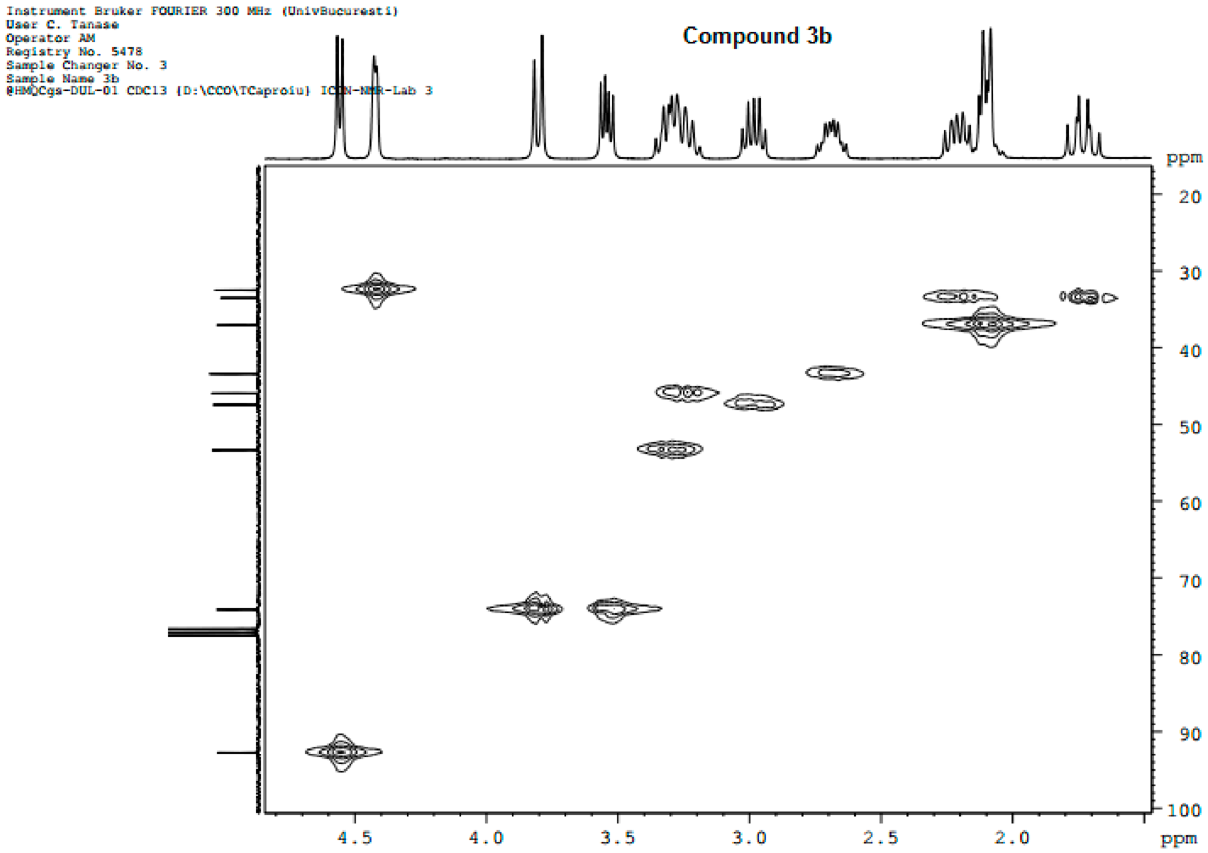

1.3. ^{1}H, ^{13}C, COSY and HETCOR-NMR spectra in $CDCl_3$ of the compound **3c**.

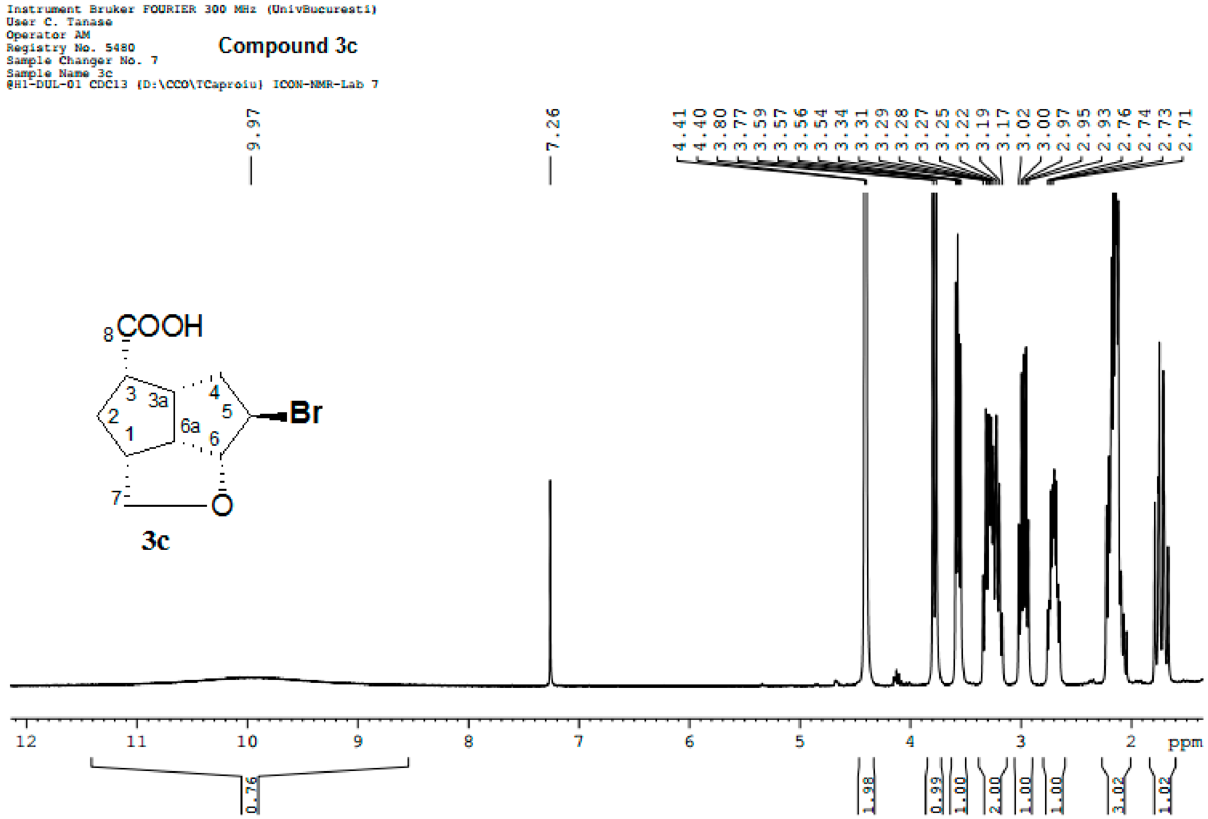

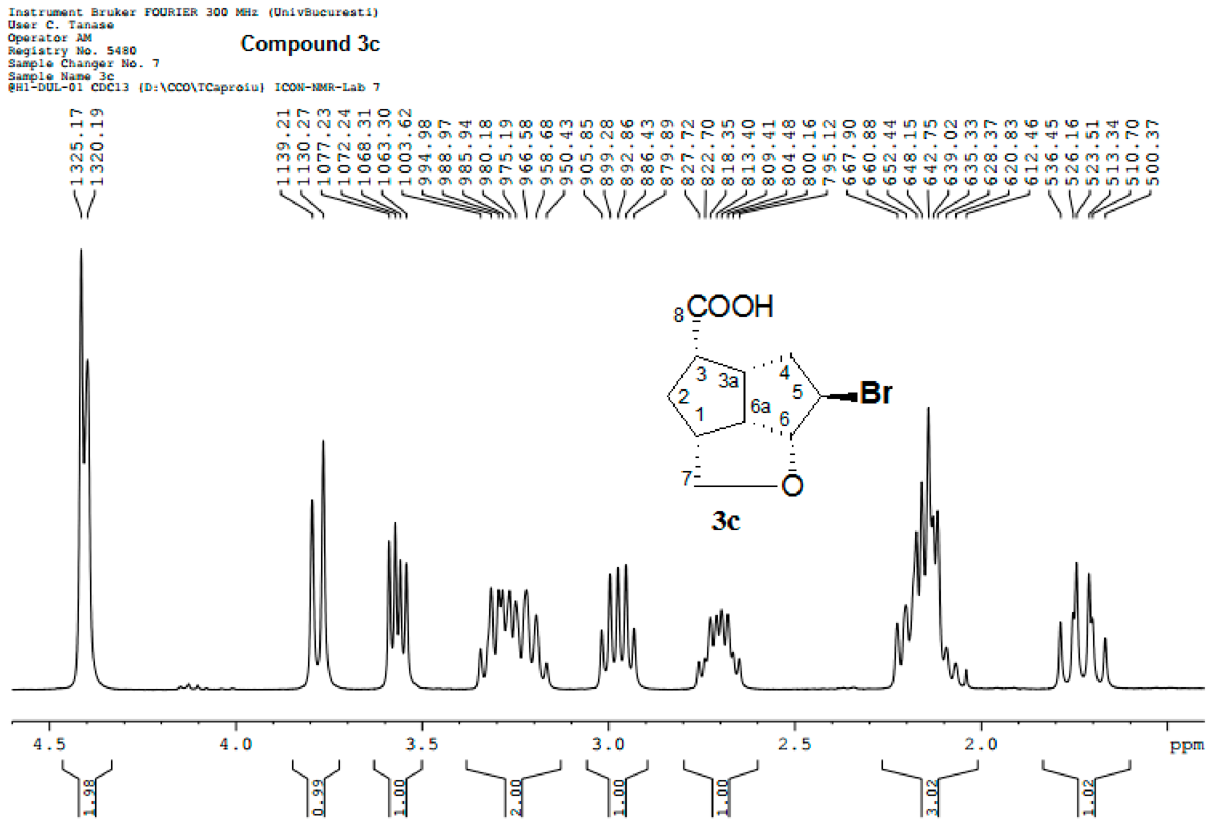
Instrument Bruker FOURIER 300 MHz (UnivBucuresti)
User C. Tanase
Operator AM
Registry No. 5480
Sample Changer No. 7
Sample Name 3c
@H1-DUL-01 CDCl3 (D:\CCO\TCaproiu) ICON-NMR-Lab 7
Compound 3c
1325.17
1320.19
1139.21
1130.27
1077.23
1072.24
1068.31
1063.30
1003.62
994.98
988.97
985.94
980.18
975.19
966.58
958.68
950.43
905.85
899.28
892.86
886.43
879.89
827.72
822.70
818.35
813.40
809.41
804.48
800.16
795.12
667.90
660.88
652.44
648.15
642.75
639.02
635.33
628.37
620.83
612.46
536.45
526.16
523.51
513.34
510.70
500.37
8 COOH
3
3a
4
5
Br
2
1
6a
6
7
O
3c
4.5
4.0
3.5
3.0
2.5
2.0
ppm
1.98
0.99
1.00
2.00
1.00
1.00
3.02
1.02

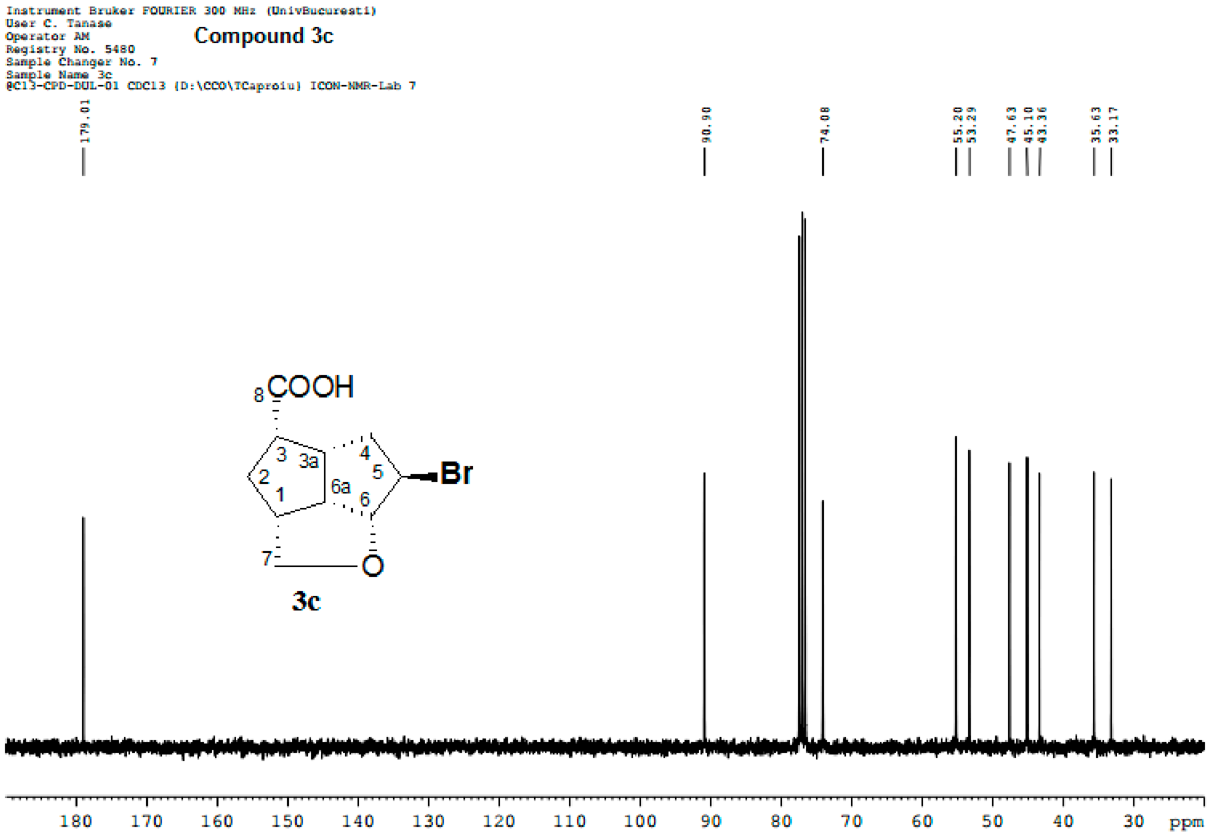
Instrument Bruker FOURIER 300 MHz (UnivBucuresti)
User C. Tanase
Operator AM
Registry No. 5480
Sample Changer No. 7
Sample Name 3c
@C13-CPD-DUL-01 CDCl3 (D:\CCO\TCaproiu) ICON-NMR-Lab 7
Compound 3c
179.01
90.90
74.08
55.20
53.29
47.63
45.10
43.36
35.63
33.17
8 COOH
3
3a
4
5
Br
2
1
6a
6
7
O
3c
180
170
160
150
140
130
120
110
100
90
80
70
60
50
40
30
ppm

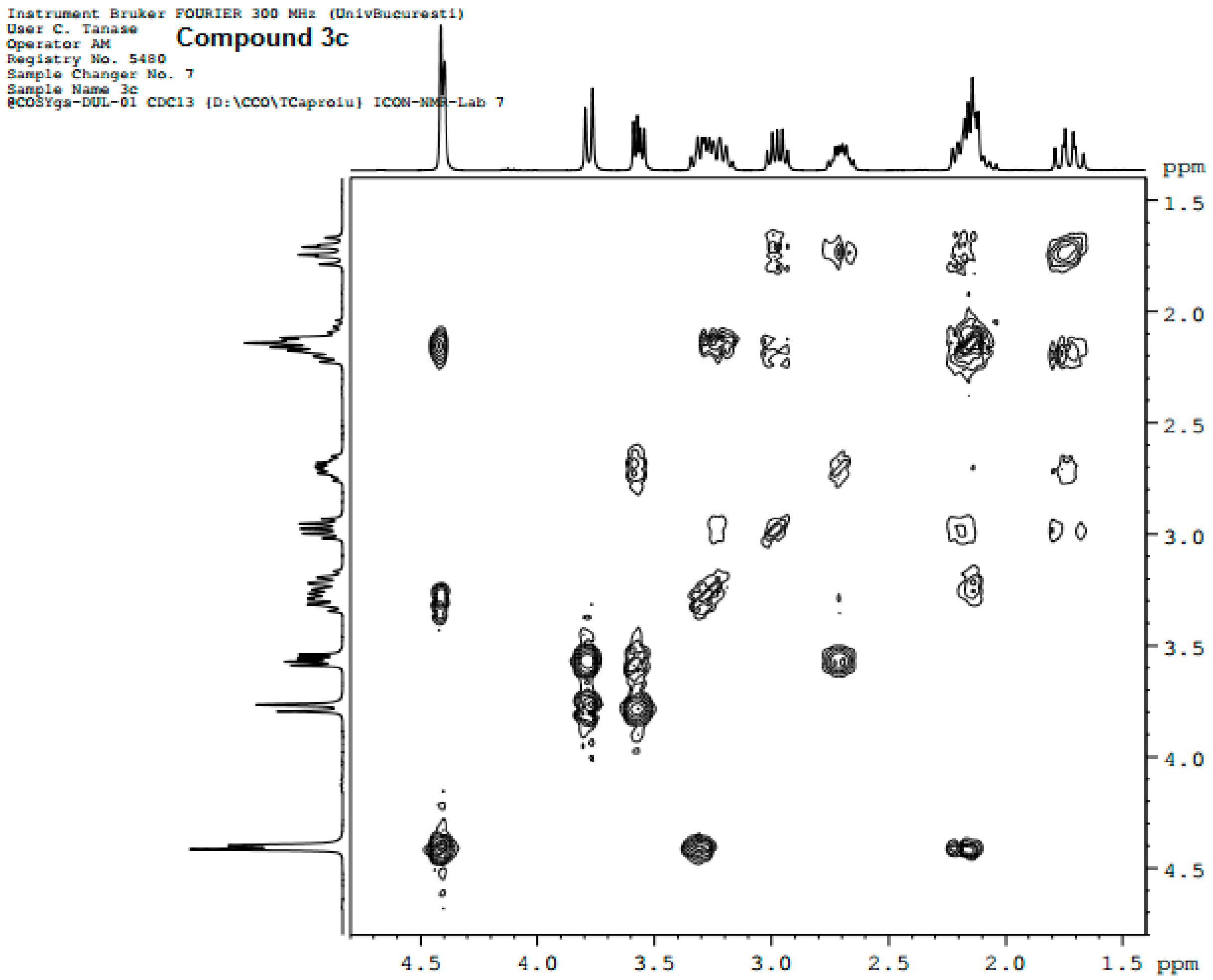
Instrument Bruker FOURIER 300 MHz (UnivBucuresti)
User C. Tanase
Operator AM
Registry No. 5480
Sample Changer No. 7
Sample Name 3c
@COSYgs-DUL-01 CDCl3 (D:\CCO\TCaproiu) ICON-NMR-Lab 7
Compound 3c
ppm
1.5
2.0
2.5
3.0
3.5
4.0
4.5
4.5
4.0
3.5
3.0
2.5
2.0
1.5 ppm

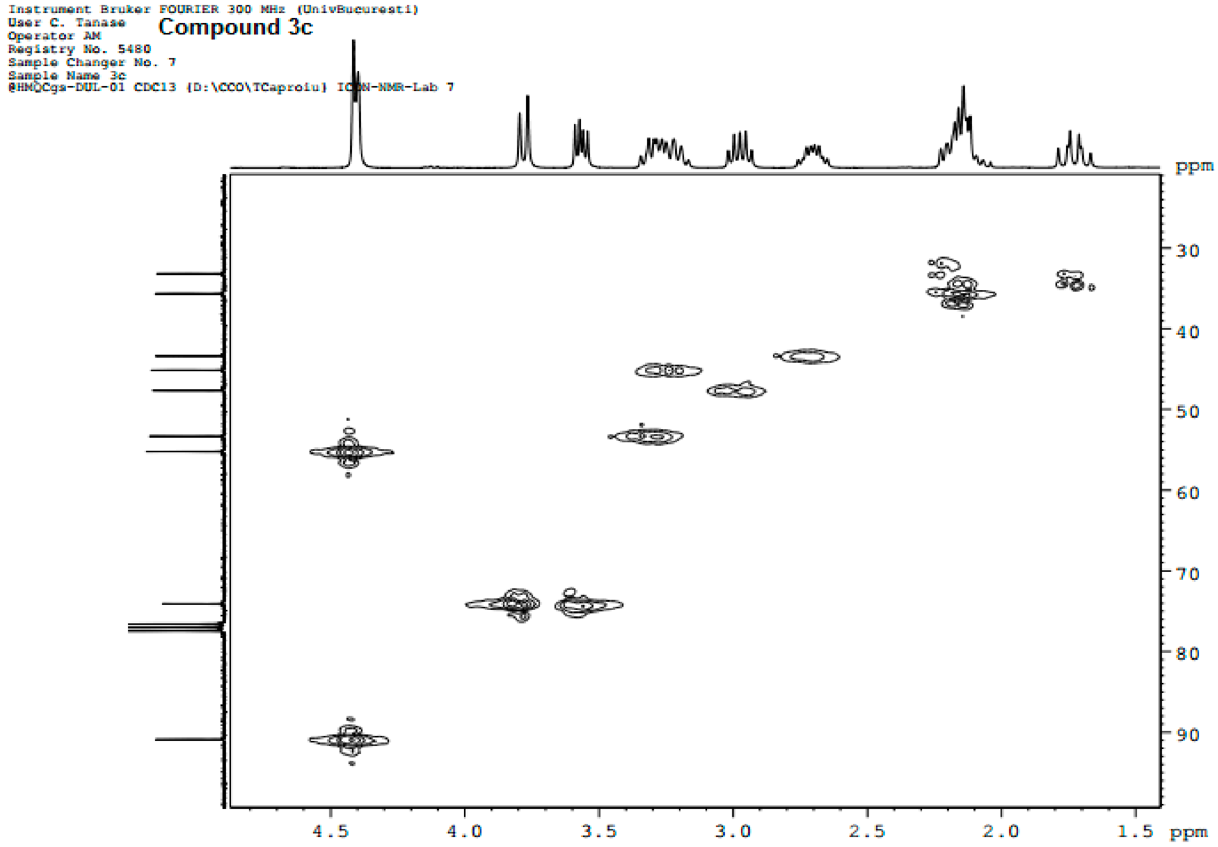
Instrument Bruker FOURIER 300 MHz (UnivBucuresti)
User C. Tanase
Operator AM
Registry No. 5480
Sample Changer No. 7
Sample Name 3c
@HMQCgs-DUL-01 CDCl3 (D:\CCO\TCaproiu) ICON-NMR-Lab 7
Compound 3c
ppm
30
40
50
60
70
80
90
4.5
4.0
3.5
3.0
2.5
2.0
1.5 ppm

1.4. ^{1}H, ^{13}C, COSY and HETCOR-NMR spectra in $CDCl_3$ of the compound **4a**.

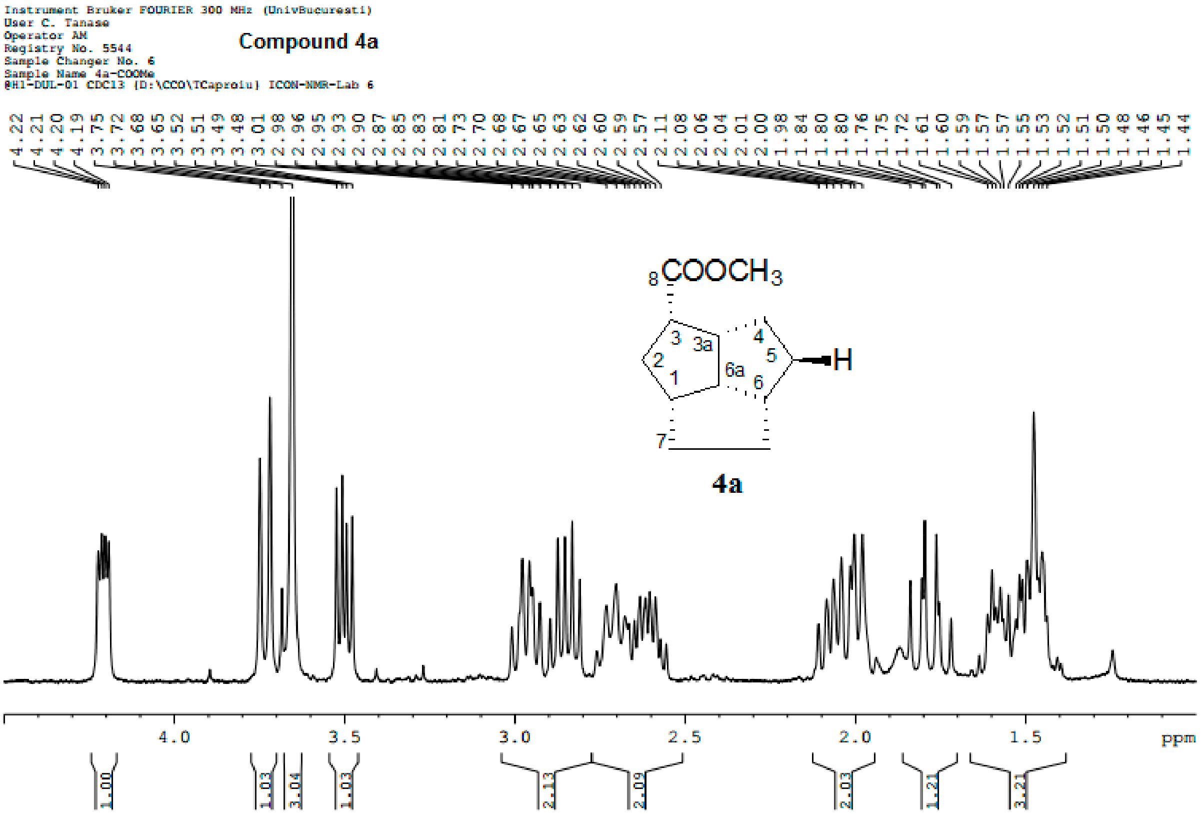

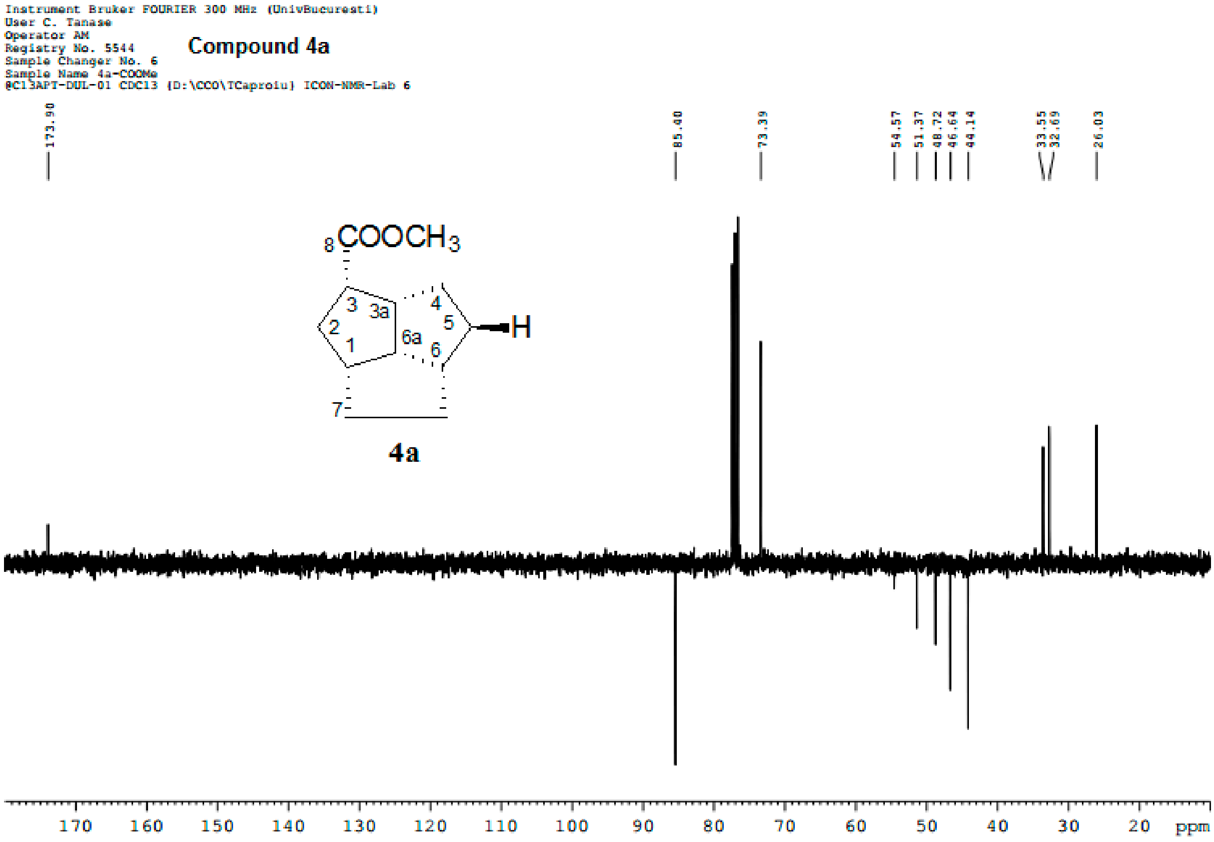

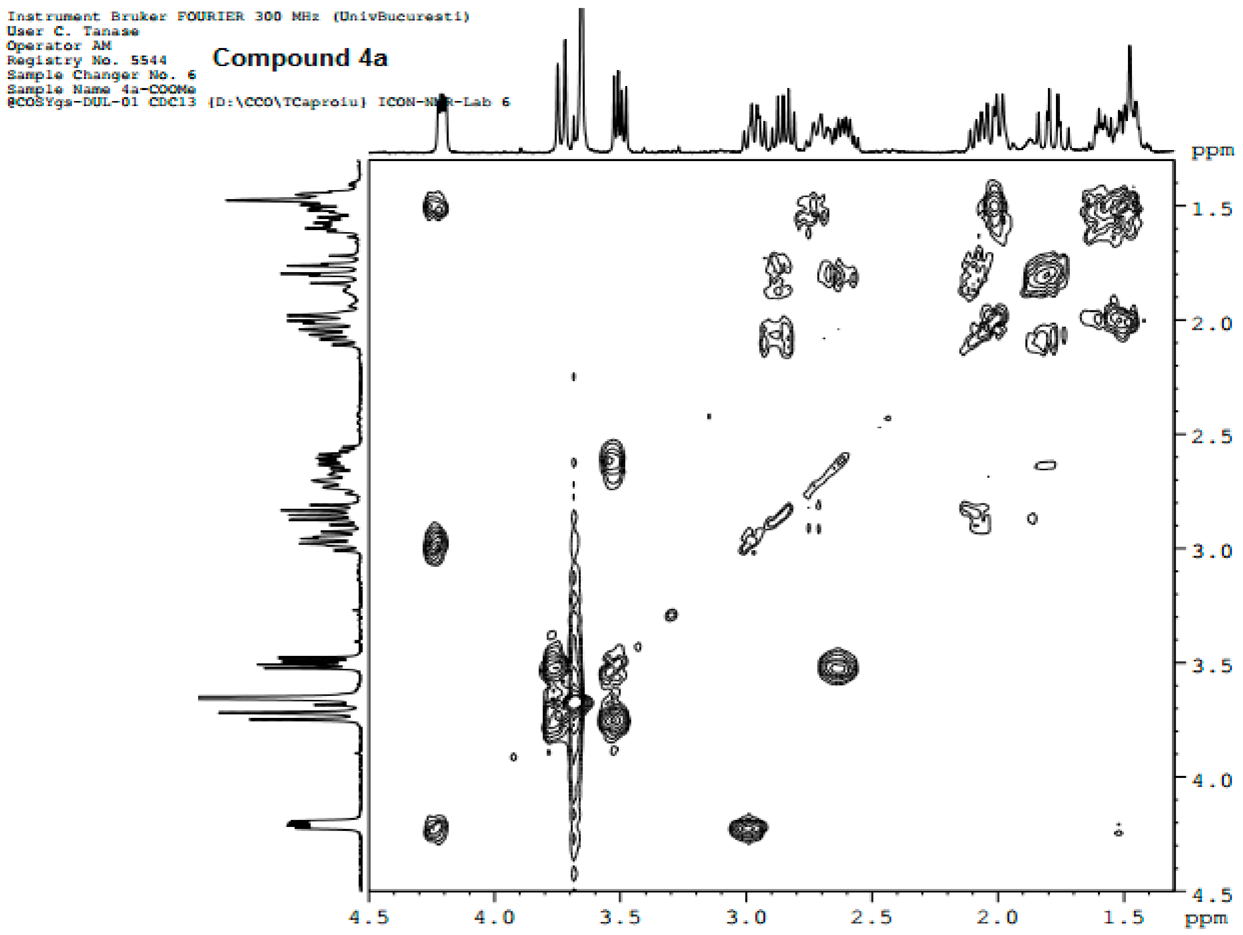
Instrument Bruker FOURIER 300 MHz (UnivBucuresti)
User C. Tanase
Operator AM
Registry No. 5544
Sample Changer No. 6
Sample Name 4a-COOMe
@COSYgs-DUL-01 CDCl3 (D:\CCO\TCaproiu) ICON-NMR-Lab 6
Compound 4a
ppm
1.5
2.0
2.5
3.0
3.5
4.0
4.5
4.5
4.0
3.5
3.0
2.5
2.0
1.5
ppm

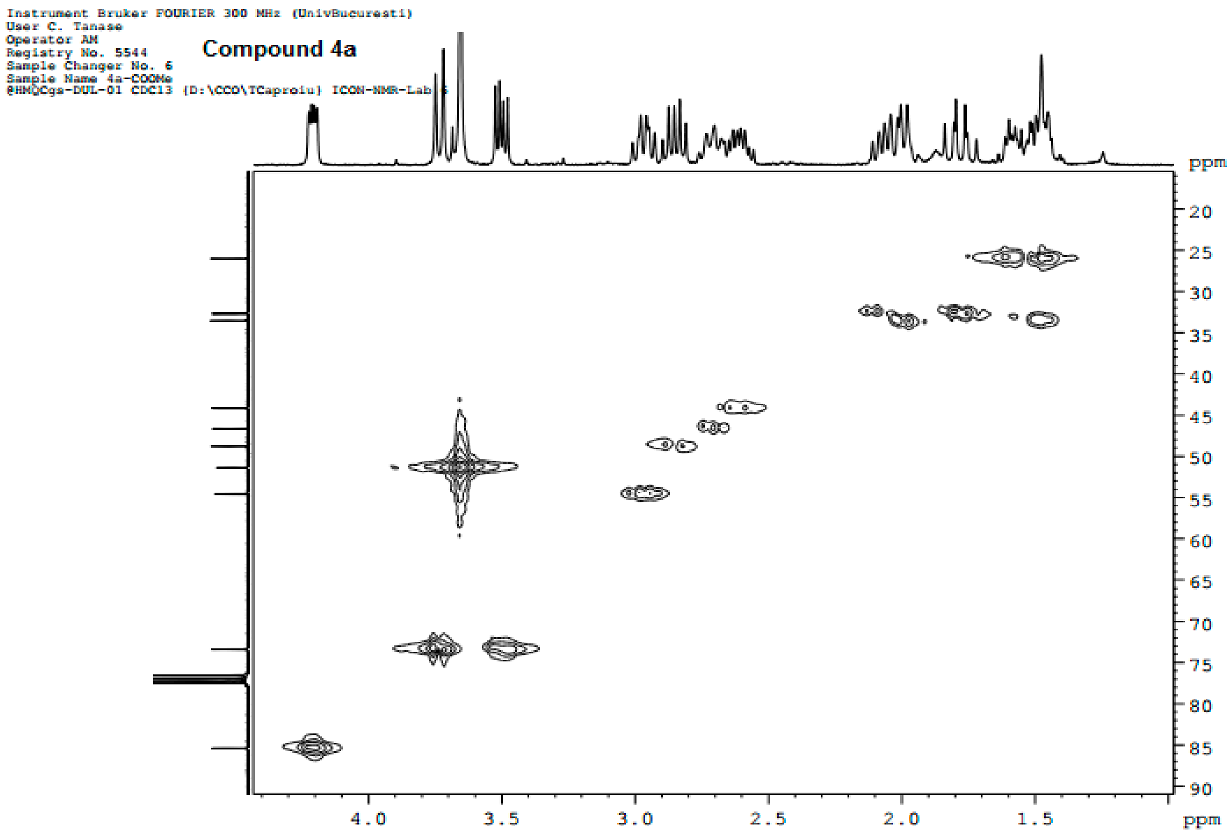
Instrument Bruker FOURIER 300 MHz (UnivBucuresti)
User C. Tanase
Operator AM
Registry No. 5544
Sample Changer No. 6
Sample Name 4a-COOMe
@HMQCgs-DUL-01 CDCl3 (D:\CCO\TCaproiu) ICON-NMR-Lab 6
Compound 4a
ppm
20
25
30
35
40
45
50
55
60
65
70
75
80
85
90
4.0
3.5
3.0
2.5
2.0
1.5
ppm

1.5. ^{1}H, ^{13}C, COSY and HETCOR-NMR spectra in $CDCl_3$ of the compound **4b**.

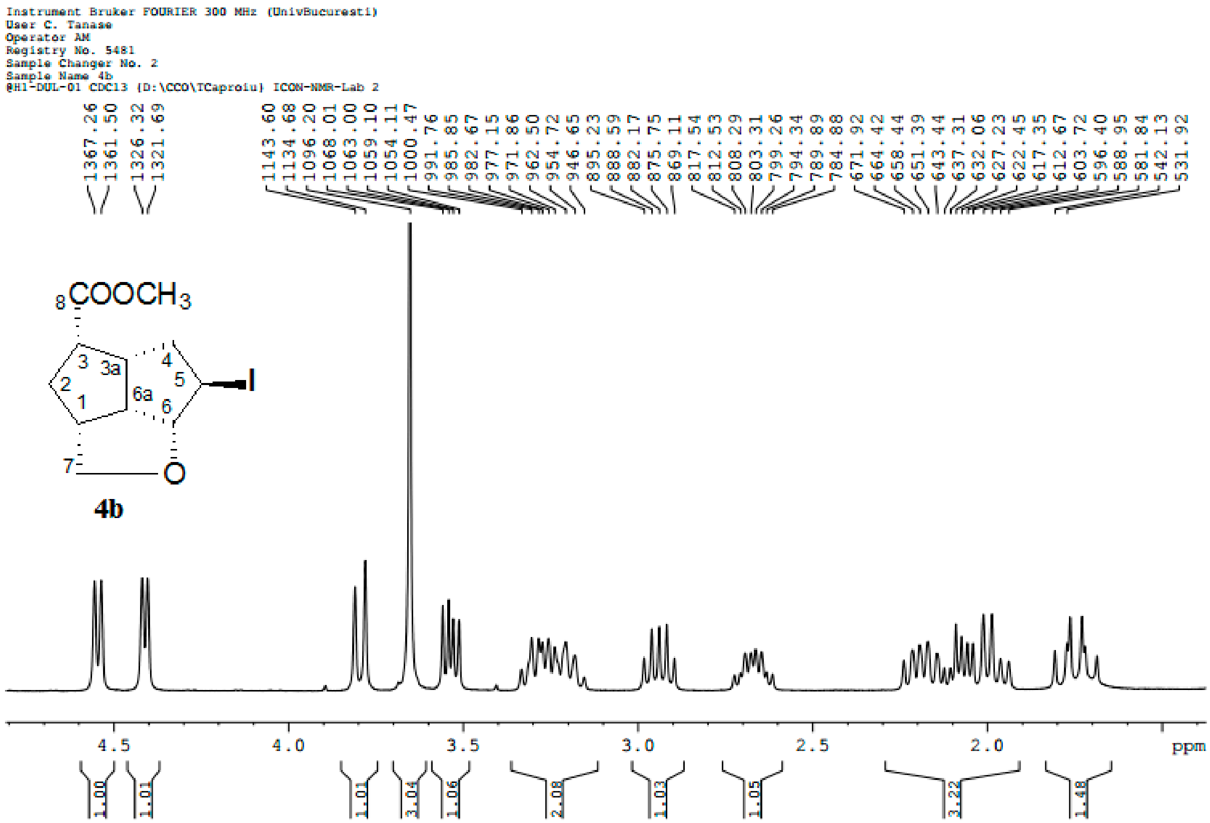

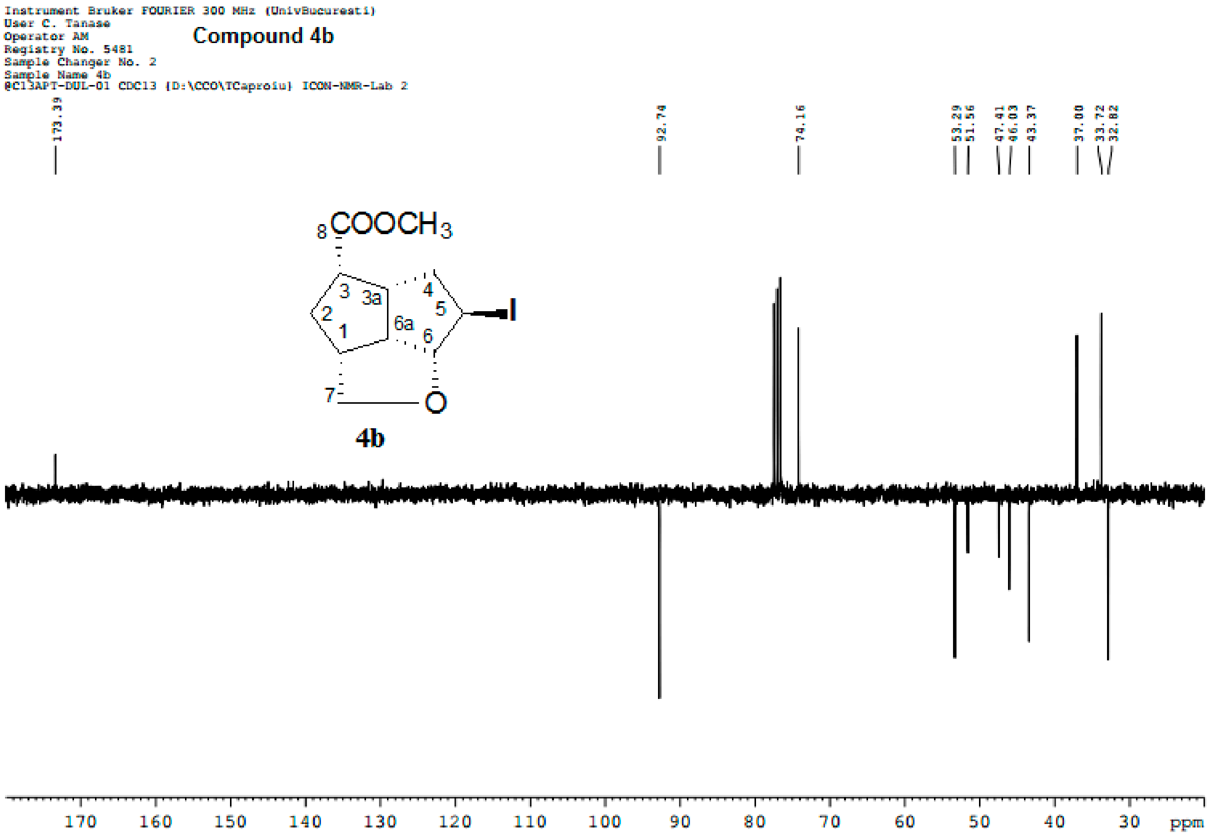

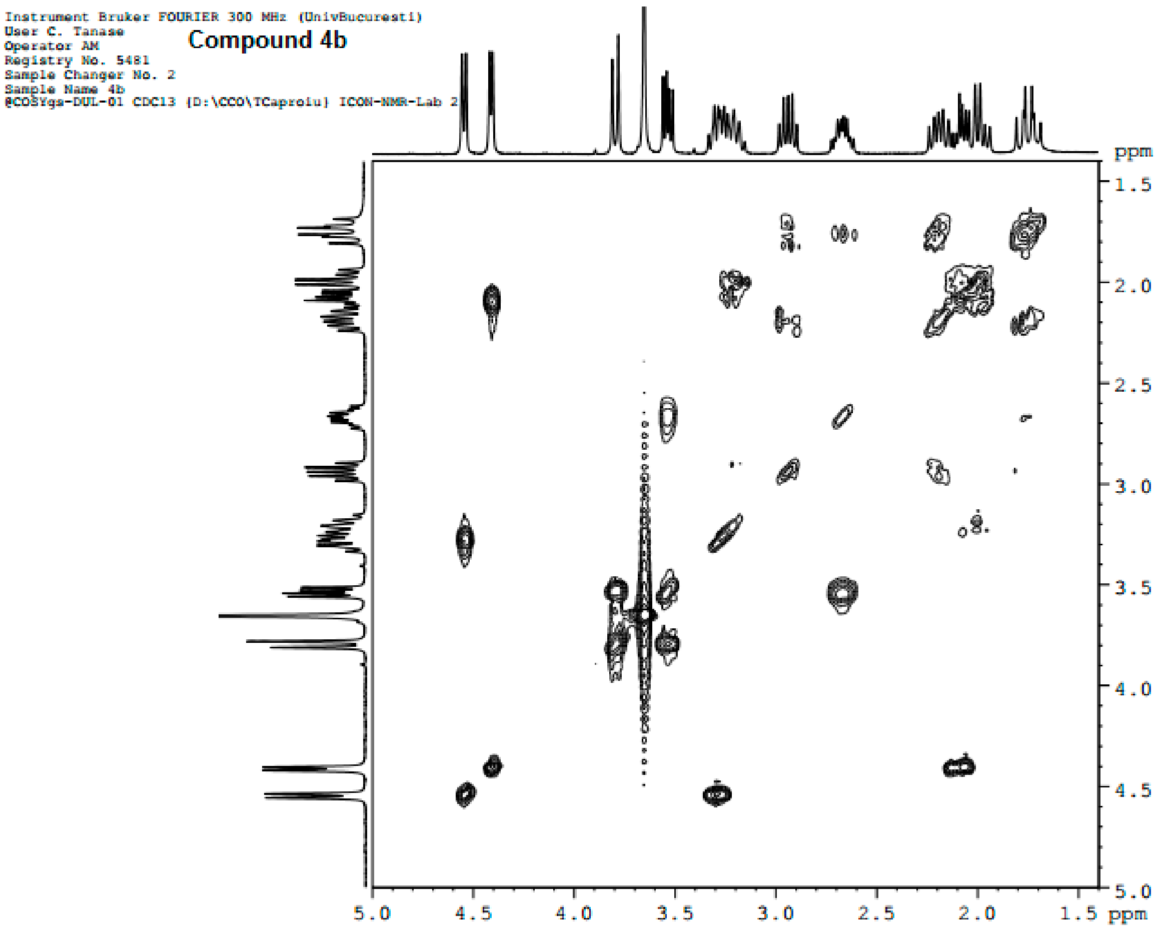
Instrument Bruker FOURIER 300 MHz (UnivBucuresti)
User C. Tanase
Operator AM
Registry No. 5481
Sample Changer No. 2
Sample Name 4b
@COSYgs-DUL-01 CDC13 {D:\CCO\TCaproiu} ICON-NMR-Lab 2
Compound 4b
ppm
1.5
2.0
2.5
3.0
3.5
4.0
4.5
5.0
5.0
4.5
4.0
3.5
3.0
2.5
2.0
1.5
ppm

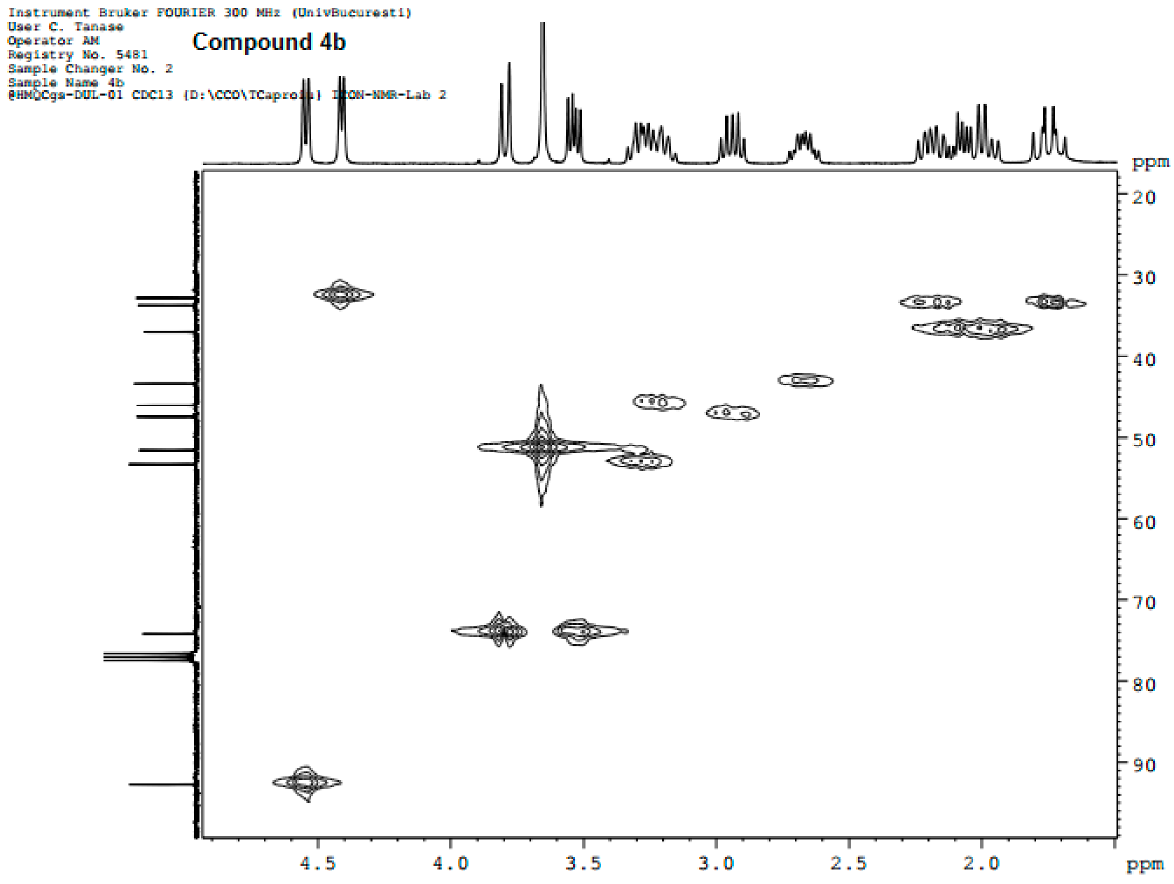
Instrument Bruker FOURIER 300 MHz (UnivBucuresti)
User C. Tanase
Operator AM
Registry No. 5481
Sample Changer No. 2
Sample Name 4b
@HMQCgs-DUL-01 CDC13 {D:\CCO\TCaproiu} ICON-NMR-Lab 2
Compound 4b
ppm
20
30
40
50
60
70
80
90
4.5
4.0
3.5
3.0
2.5
2.0
ppm

1.6. ^{1}H, ^{13}C, COSY and HETCOR-NMR spectra in $CDCl_3$ of the compound **4c**.

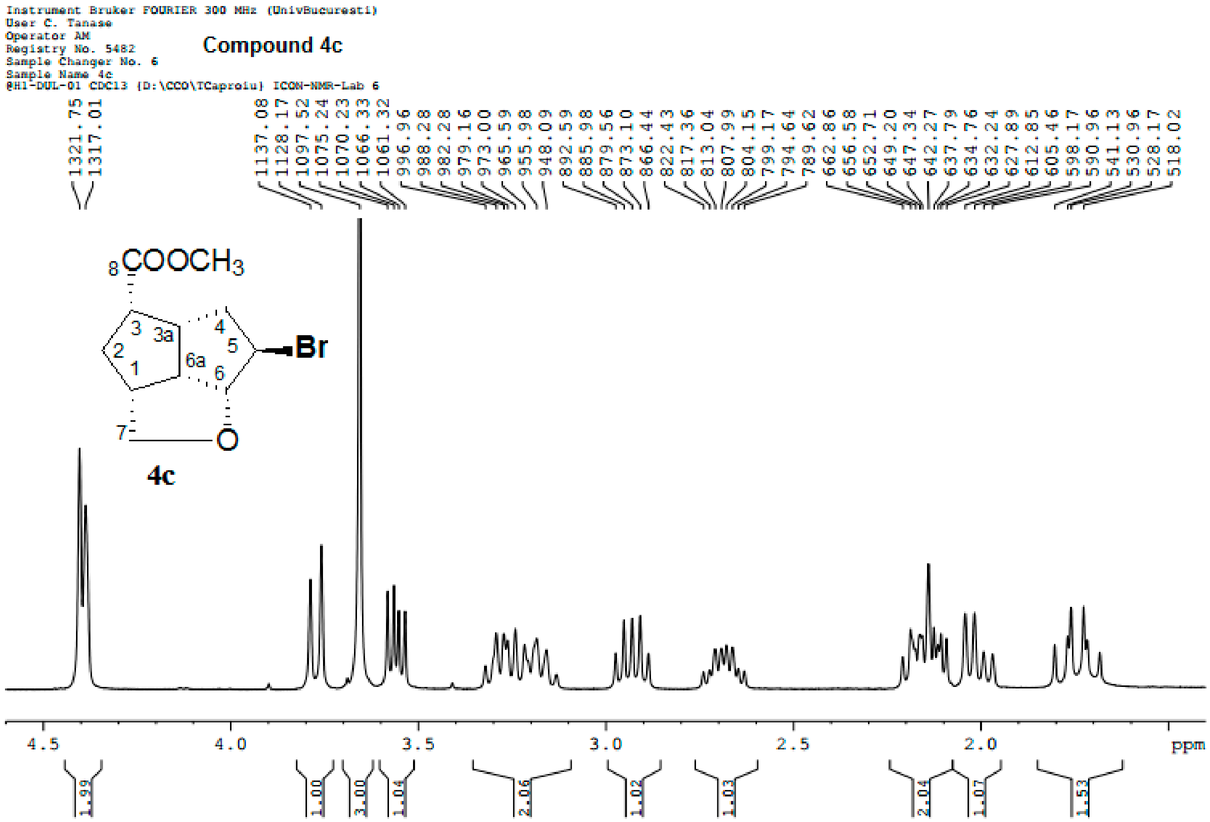

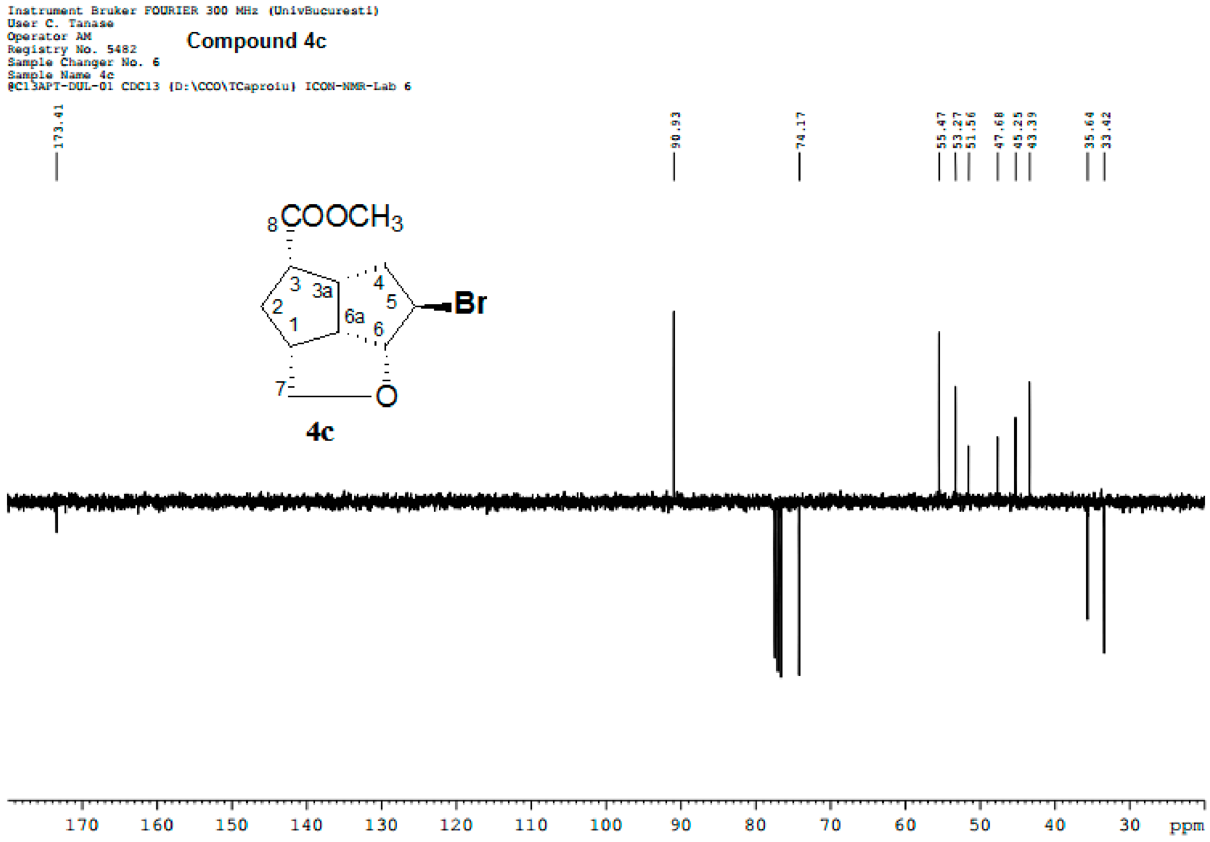

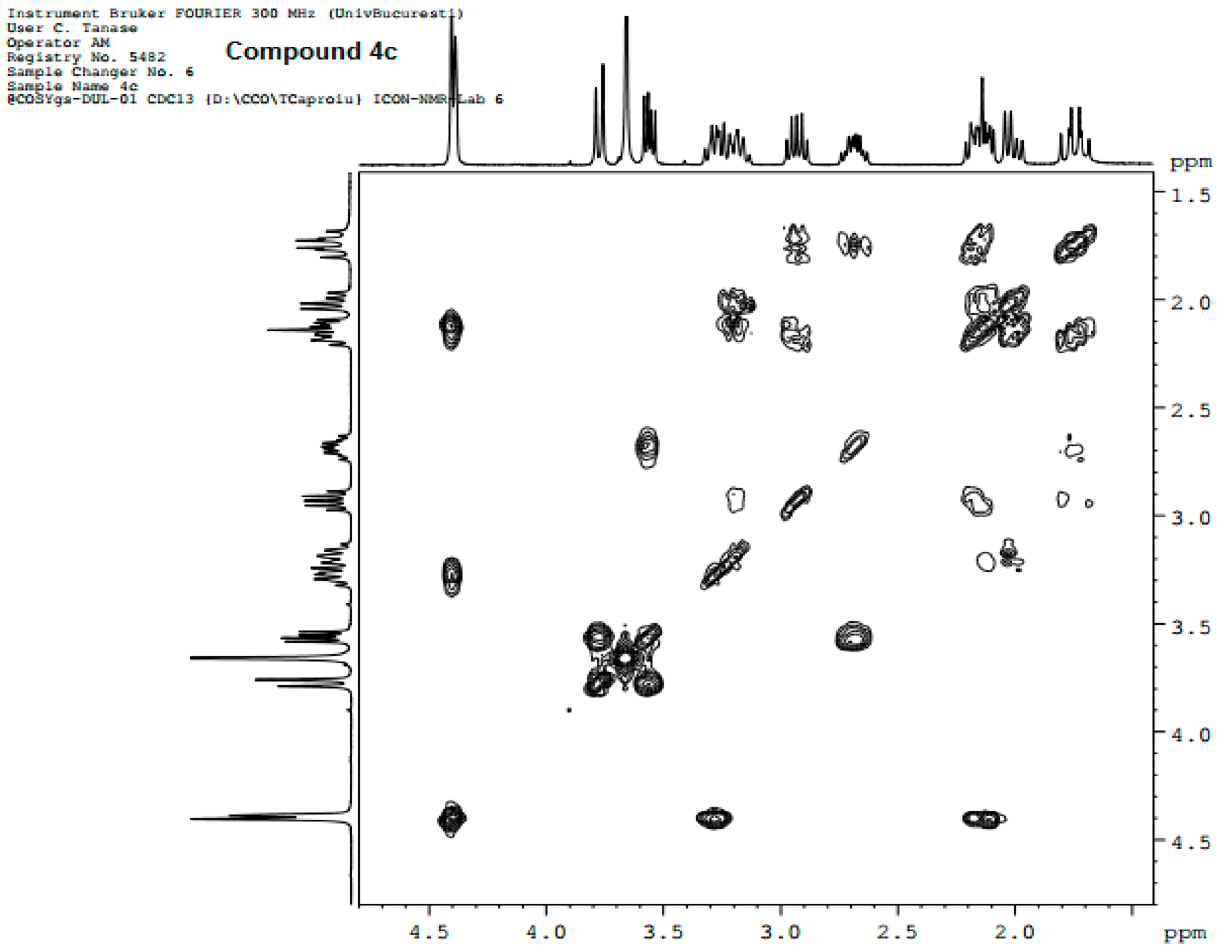
Instrument Bruker FOURIER 300 MHz (UnivBucuresti)
User C. Tanase
Operator AM
Registry No. 5482
Sample Changer No. 6
Sample Name 4c
@COSYgs-DUL-01 CDCl3 (D:\CCO\TCaproiu) ICON-NMR-Lab 6
Compound 4c
ppm
1.5
2.0
2.5
3.0
3.5
4.0
4.5
4.5
4.0
3.5
3.0
2.5
2.0
ppm

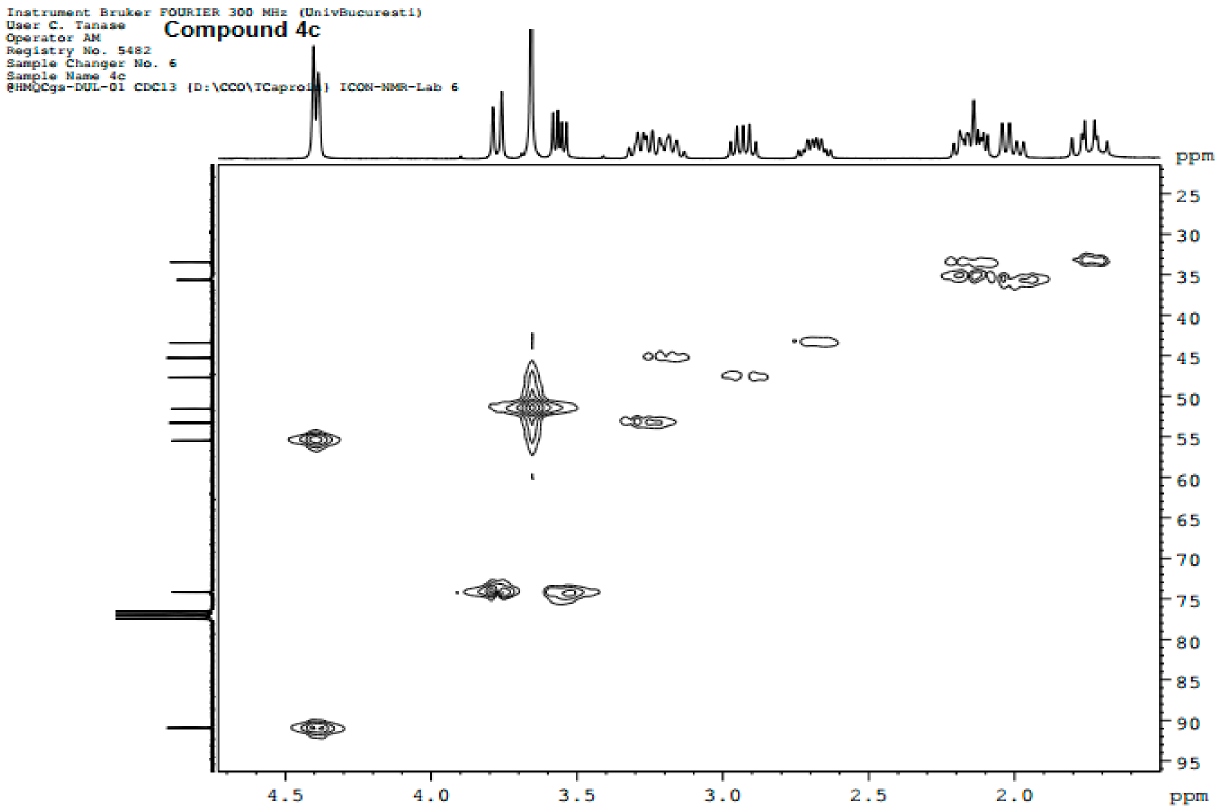
Instrument Bruker FOURIER 300 MHz (UnivBucuresti)
User C. Tanase
Operator AM
Registry No. 5482
Sample Changer No. 6
Sample Name 4c
@HMQCgs-DUL-01 CDCl3 (D:\CCO\TCaproiu) ICON-NMR-Lab 6
Compound 4c
ppm
25
30
35
40
45
50
55
60
65
70
75
80
85
90
95
4.5
4.0
3.5
3.0
2.5
2.0
ppm

1.7. ^{1}H, ^{13}C, COSY and HETCOR-NMR spectra in $CDCl_3$ of the compound **5a** (Varian)

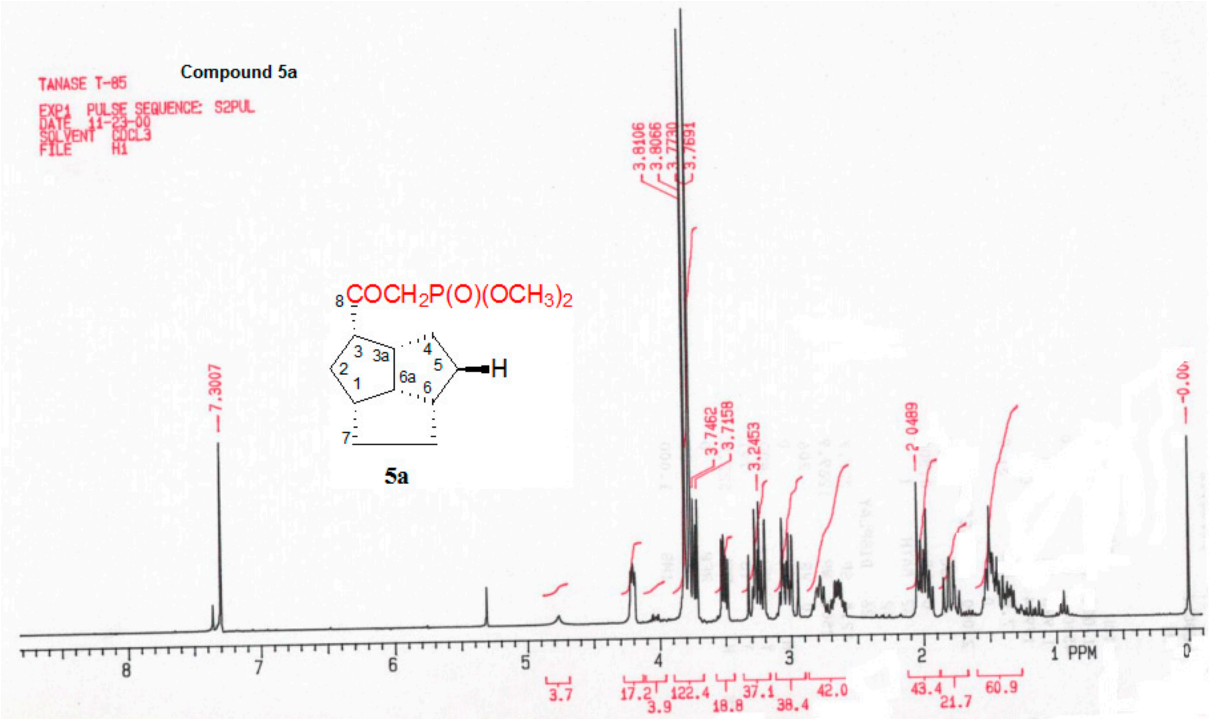

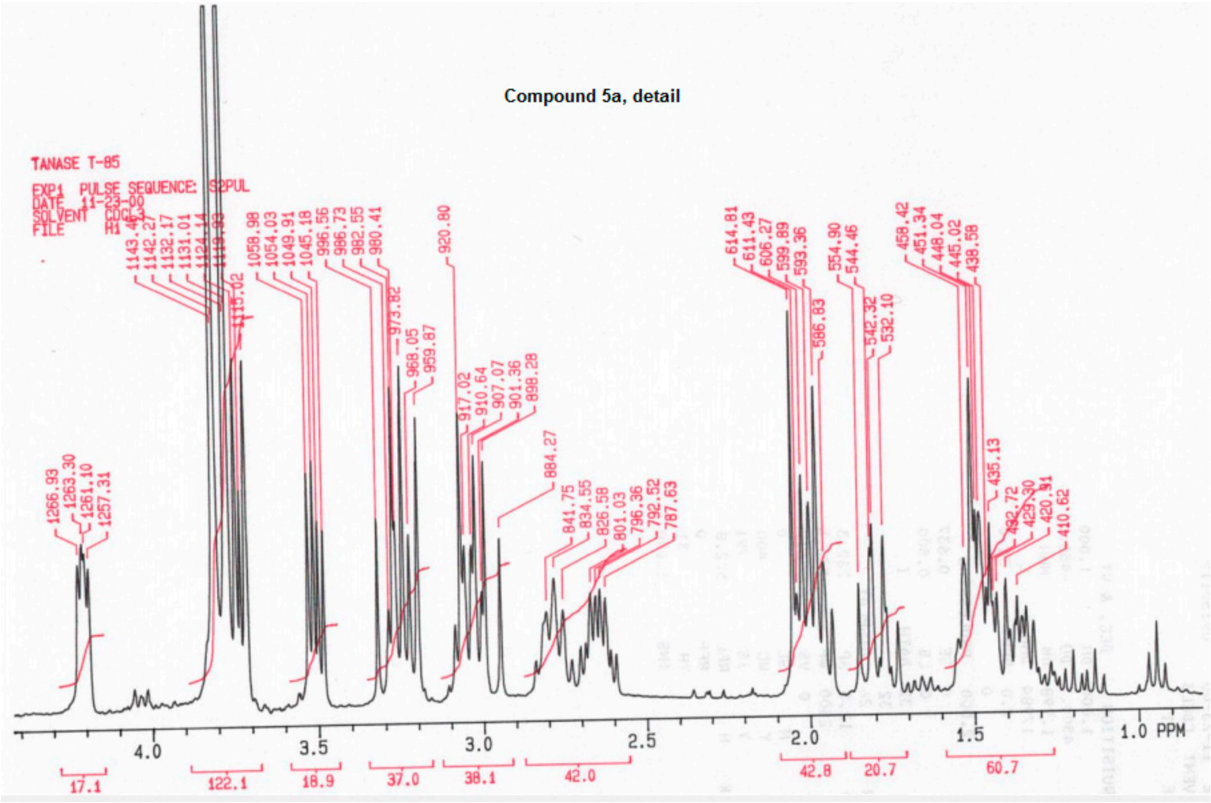

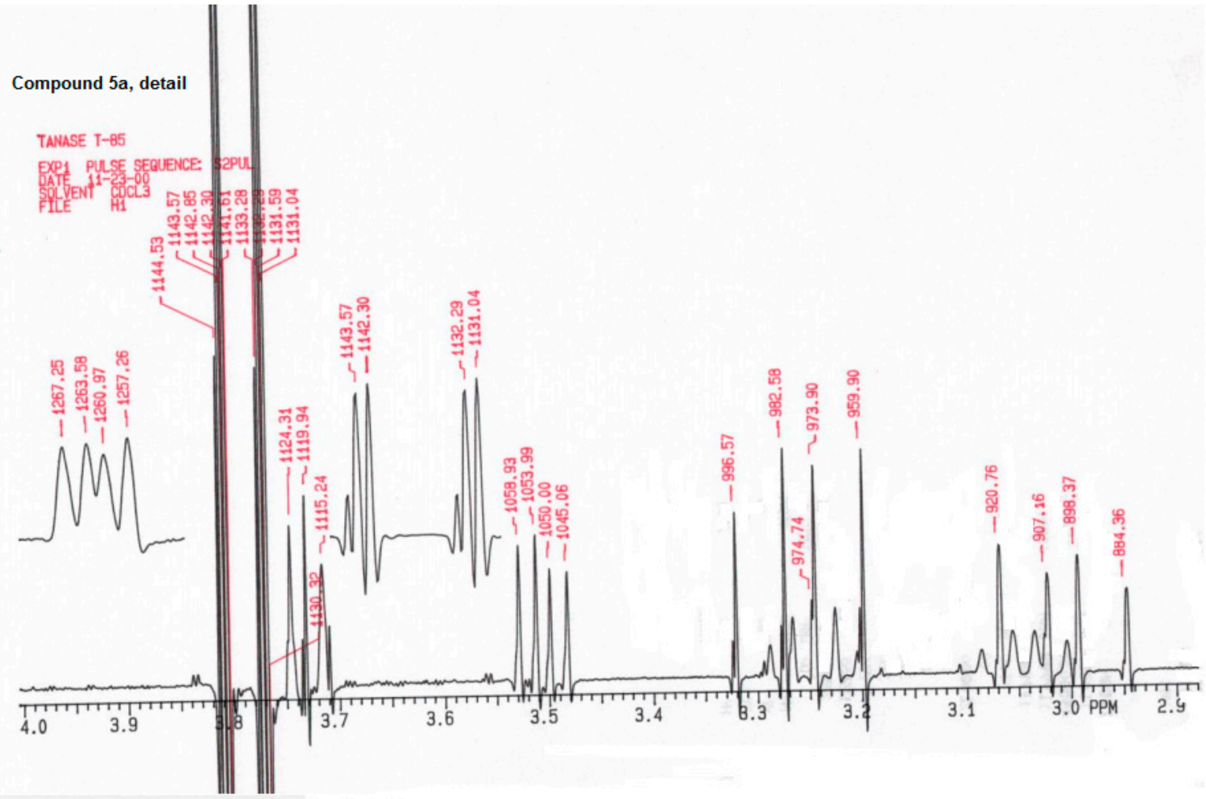
Compound 5a, detail
TANASE T-85
EXP1 PULSE SEQUENCE: S2PUL
DATE 11-23-00
SOLVENT CDCL3
FILE H1
1267.25
1263.58
1260.97
1257.26
1144.53
1143.57
1142.85
1142.30
1141.61
1133.28
1131.59
1131.04
1124.31
1119.94
1115.24
1130.32
1143.57
1142.30
1132.29
1131.04
1058.93
1053.99
1050.00
1045.06
996.57
982.58
974.74
973.90
959.90
920.76
907.16
898.37
884.36
4.0
3.9
3.8
3.7
3.6
3.5
3.4
3.3
3.2
3.1
3.0 PPM
2.9

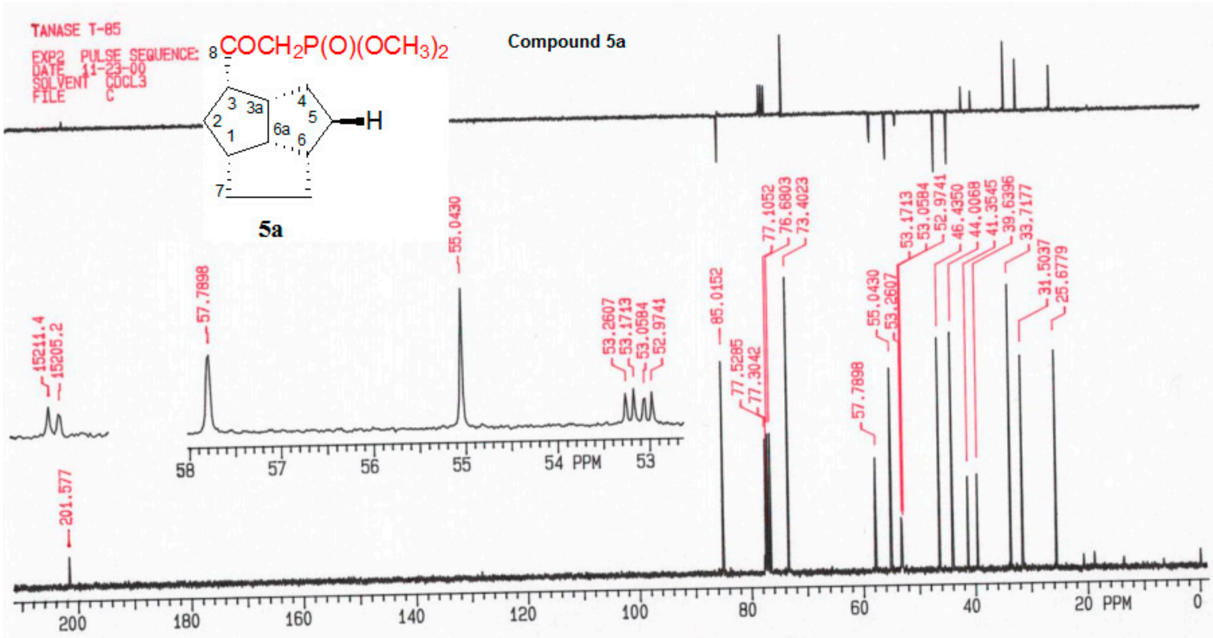
TANASE T-85
EXP2 PULSE SEQUENCE: S2PUL
DATE 11-23-00
SOLVENT CDCL3
FILE C
Compound 5a
8 COCH2P(O)(OCH3)2
5a
15211.4
15205.2
57.7898
55.0430
53.2607
53.1713
53.0584
52.9741
58
57
56
55
54 PPM
53
201.577
85.0152
77.5285
77.3042
77.1052
76.6803
73.4023
57.7898
55.0430
53.2607
53.1713
53.0584
52.9741
46.4350
44.0068
41.3545
39.6396
33.7177
31.5037
25.6779
200
180
160
140
120
100
80
60
40
20 PPM
0

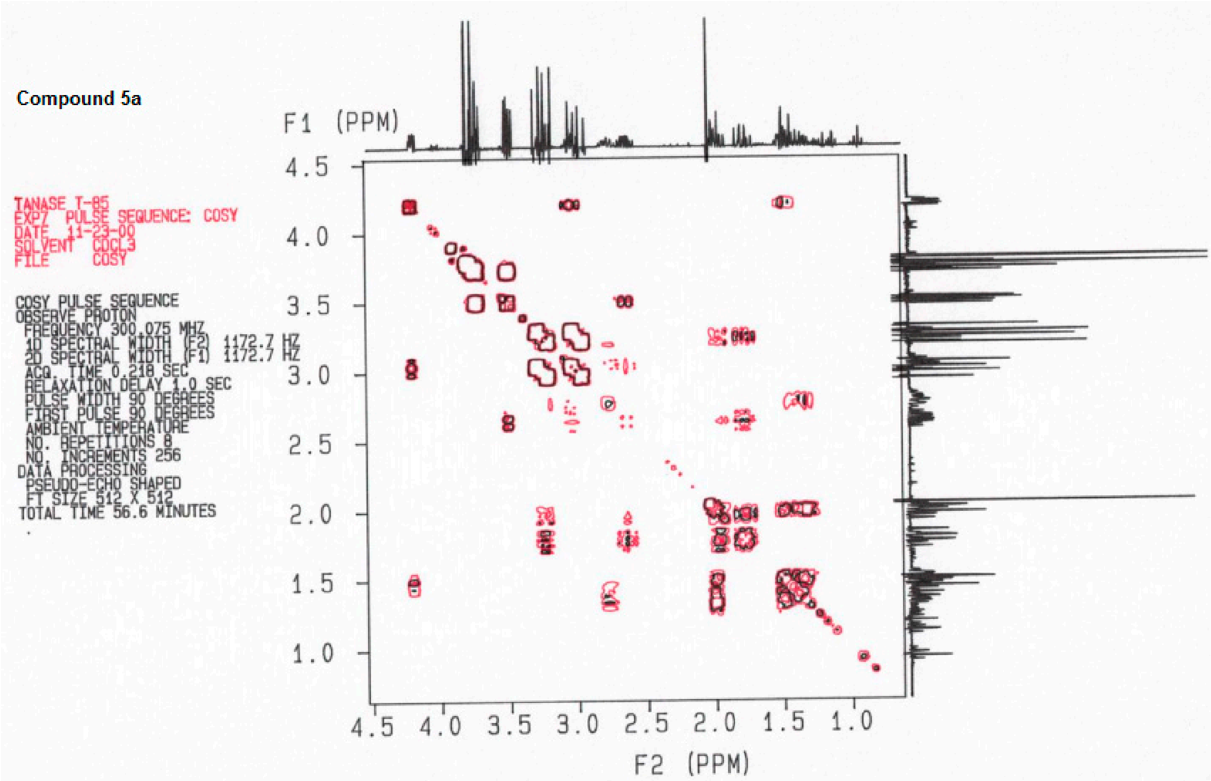

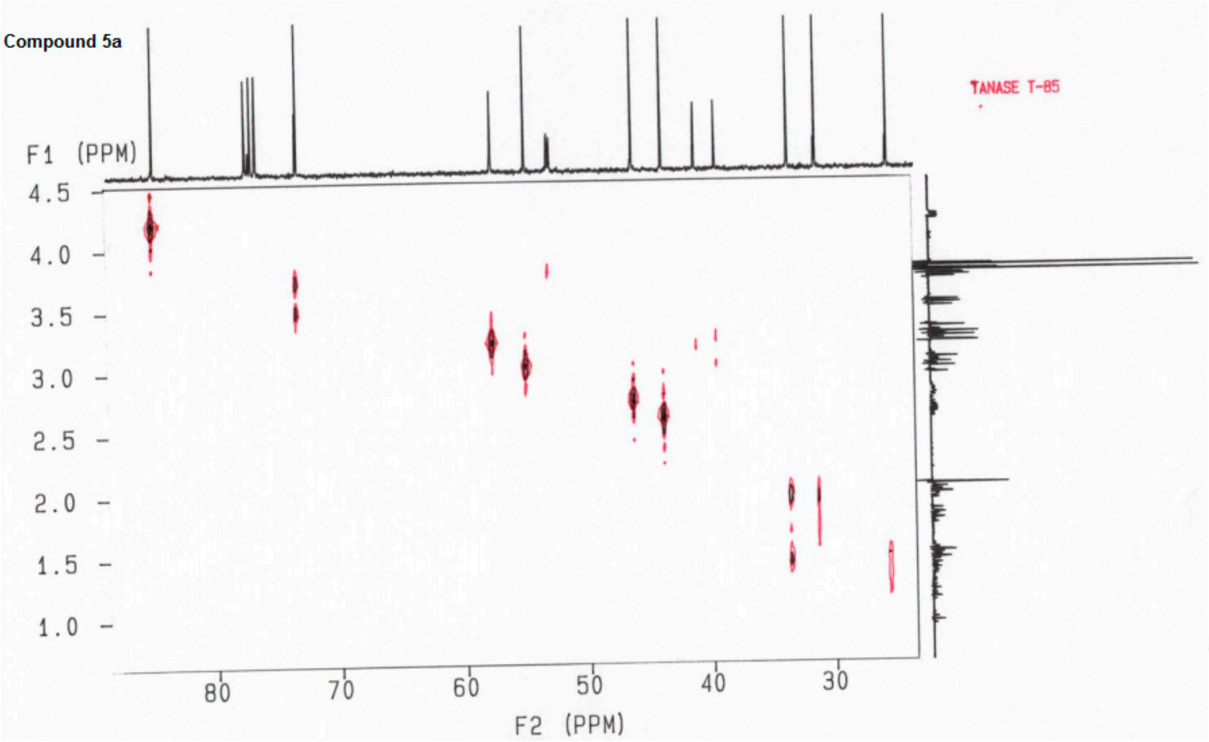

1.8. ^{1}H, ^{13}C, COSY and HETCOR-NMR spectra in $CDCl_3$ of the compound **5b**.

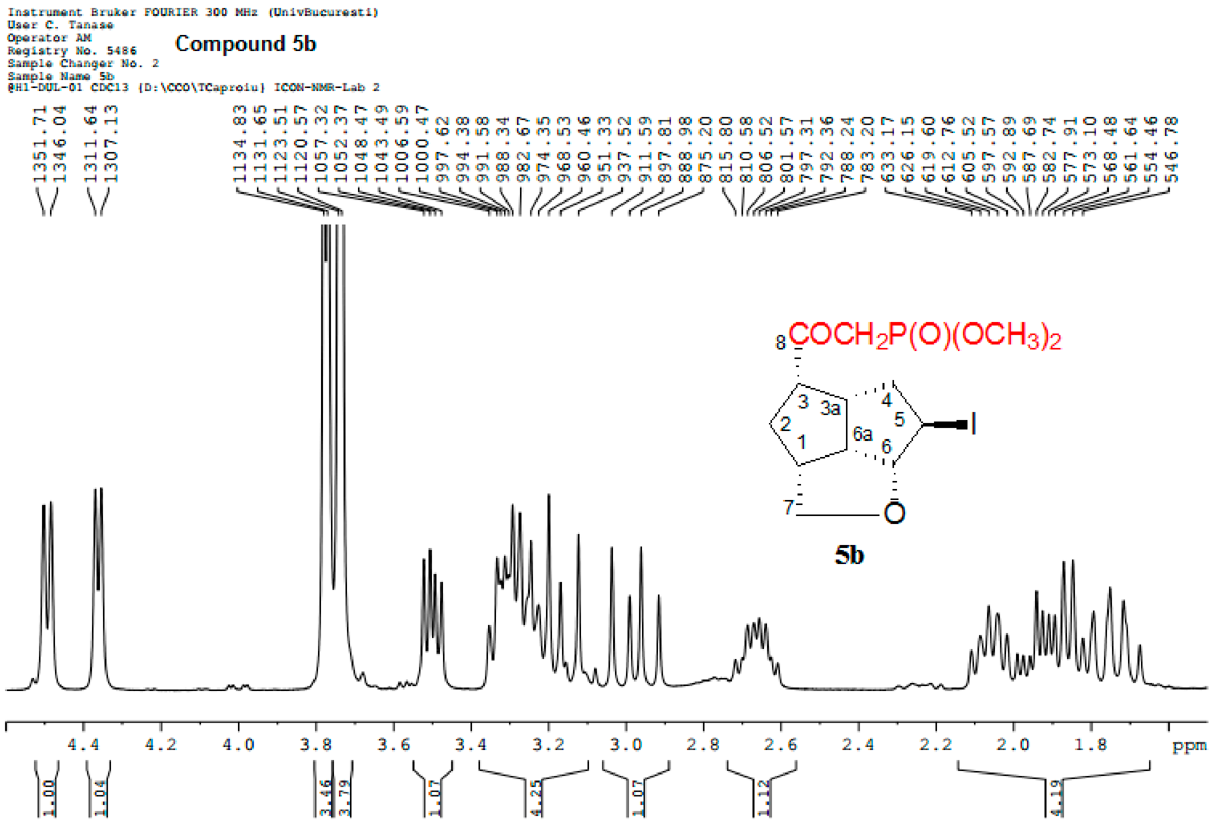
Instrument Bruker FOURIER 300 MHz (UnivBucuresti)
User C. Tanase
Operator AM
Registry No. 5486
Sample Changer No. 2
Sample Name 5b
@H1-DUL-01 CDCl3 (D:\CCO\TCaproiu) ICON-NMR-Lab 2
Compound 5b
1351.71 1346.04 1311.64 1307.13
1134.83 1131.65 1123.51 1120.57 1057.32 1052.37 1048.47 1043.49 1006.59 1000.47 997.62 994.38 991.58 988.34 982.67 974.35 968.53 960.46 951.33 937.52 911.59 897.81 888.98 875.20 815.80 810.58 806.52 801.57 797.31 792.36 788.24 783.20 633.17 626.15 619.60 612.76 605.52 597.57 592.89 587.69 582.74 577.91 573.10 568.48 561.64 554.46 546.78
8COCH2P(O)(OCH3)2
3 3a 4 5 2 1 6a 6 7 O I
5b
4.4 4.2 4.0 3.8 3.6 3.4 3.2 3.0 2.8 2.6 2.4 2.2 2.0 1.8 ppm
1.00 1.04 3.46 3.79 1.07 4.25 1.07 1.12 4.19

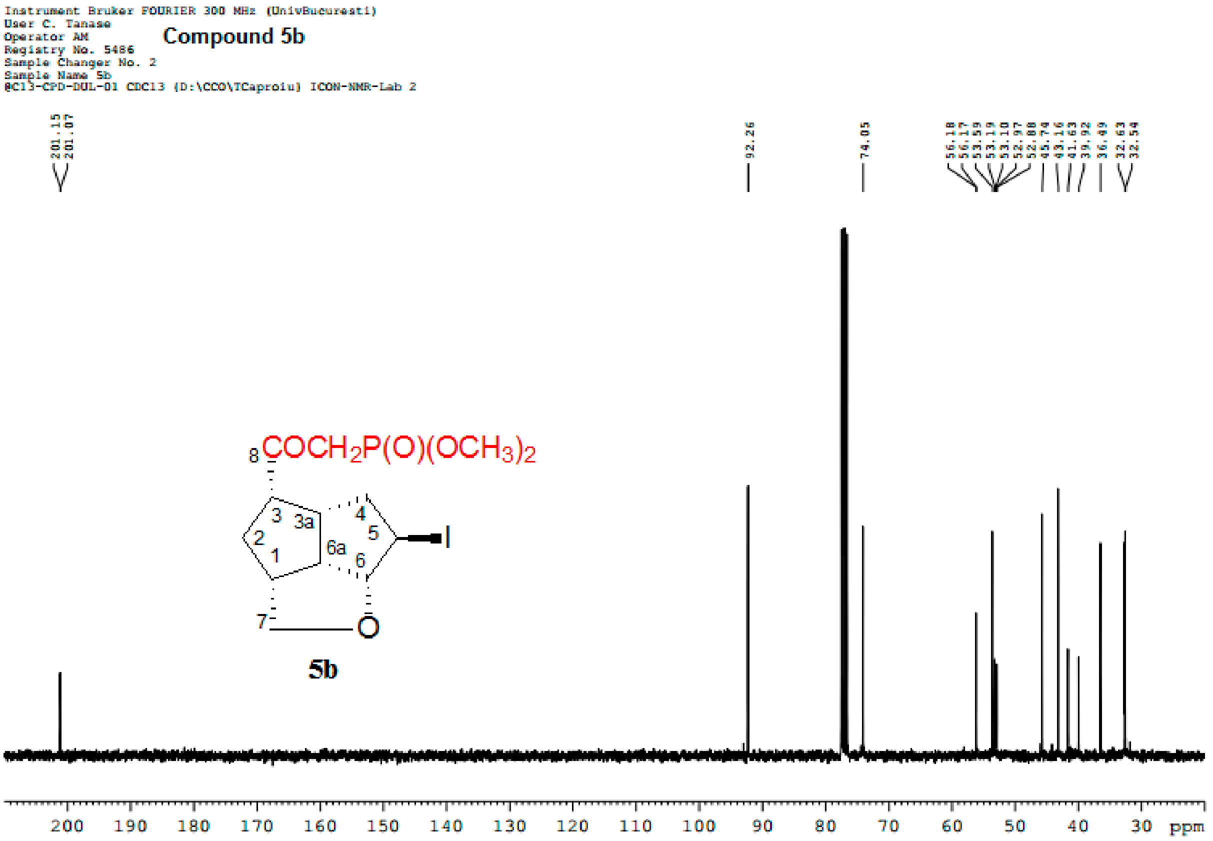
Instrument Bruker FOURIER 300 MHz (UnivBucuresti)
User C. Tanase
Operator AM
Registry No. 5486
Sample Changer No. 2
Sample Name 5b
@C13-CPD-DUL-01 CDCl3 (D:\CCO\TCaproiu) ICON-NMR-Lab 2
Compound 5b
201.15 201.07
92.26
74.05
56.18 56.17 53.59 53.19 53.10 52.97 52.88 45.74 43.16 41.63 39.92 36.49 32.63 32.54
8COCH2P(O)(OCH3)2
3 3a 4 5 2 1 6a 6 7 O I
5b
200 190 180 170 160 150 140 130 120 110 100 90 80 70 60 50 40 30 ppm

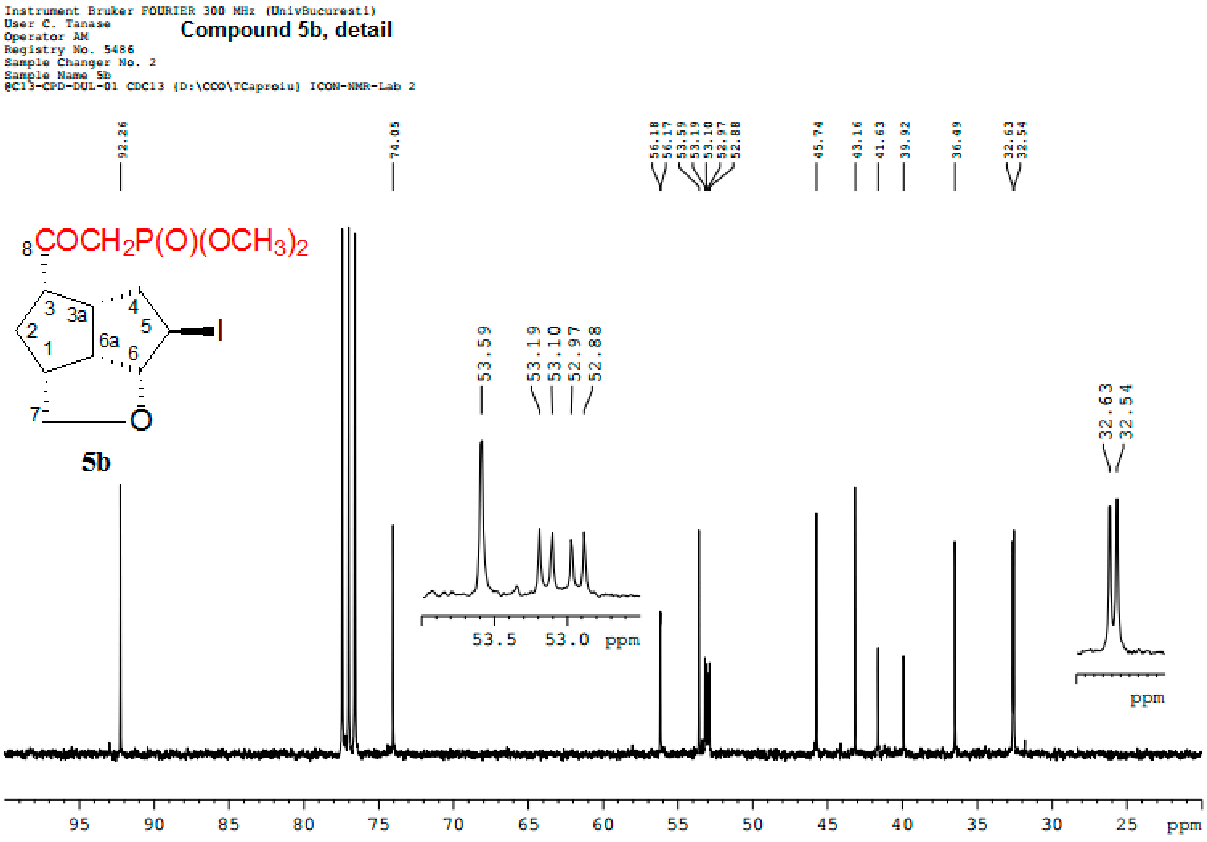
Instrument Bruker FOURIER 300 MHz (UnivBucuresti)
User C. Tanase
Operator AM
Registry No. 5486
Sample Changer No. 2
Sample Name 5b
@C13-CPD-DUL-01 CDCl3 (D:\CCO\TCaproiu) ICON-NMR-Lab 2
Compound 5b, detail
92.26
74.05
56.18
56.17
53.59
53.19
53.10
52.97
52.88
45.74
43.16
41.63
39.92
36.49
32.63
32.54
8 COCH2P(O)(OCH3)2
5b
ppm

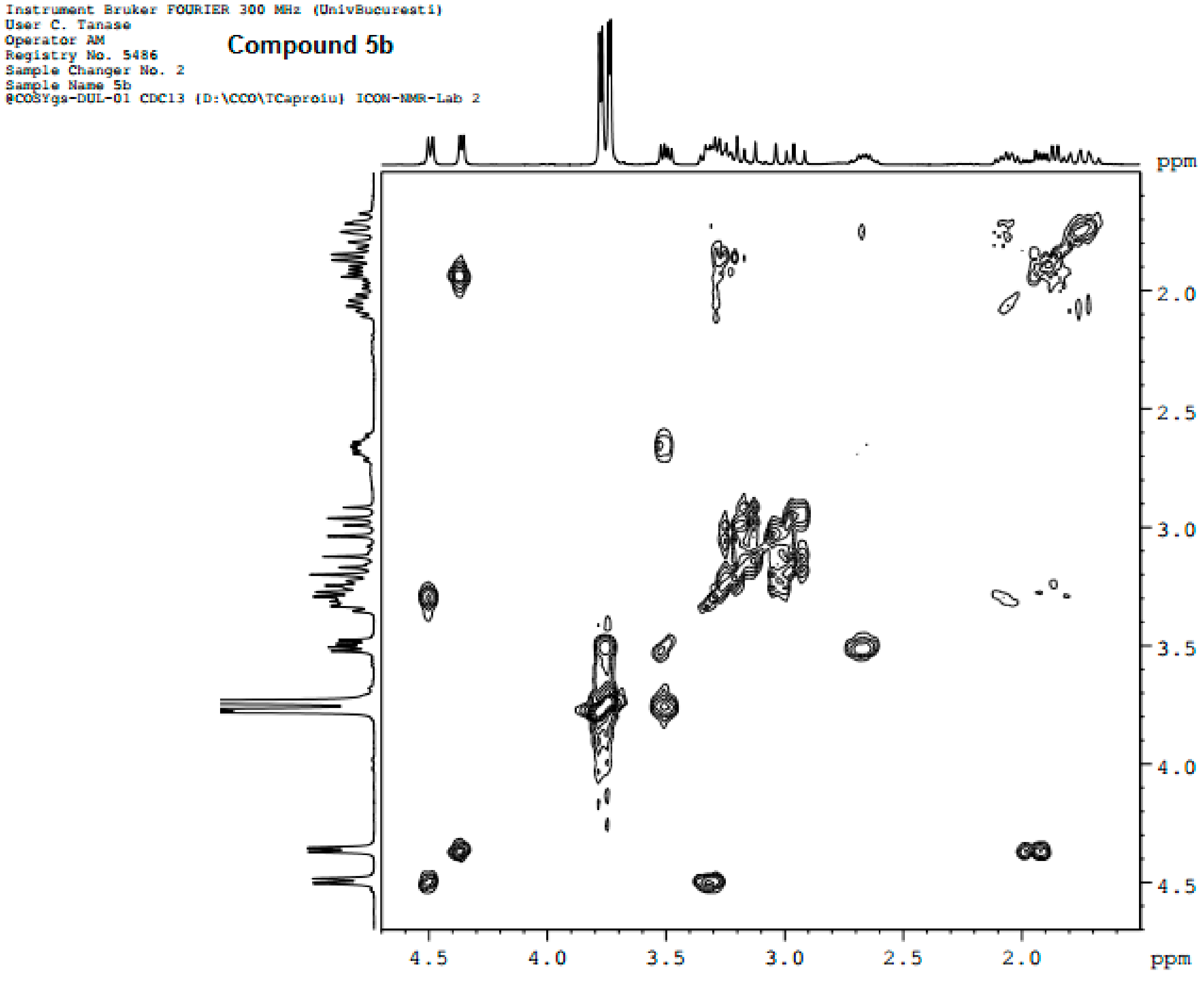
Instrument Bruker FOURIER 300 MHz (UnivBucuresti)
User C. Tanase
Operator AM
Registry No. 5486
Sample Changer No. 2
Sample Name 5b
@COSYgs-DUL-01 CDCl3 (D:\CCO\TCaproiu) ICON-NMR-Lab 2
Compound 5b
ppm

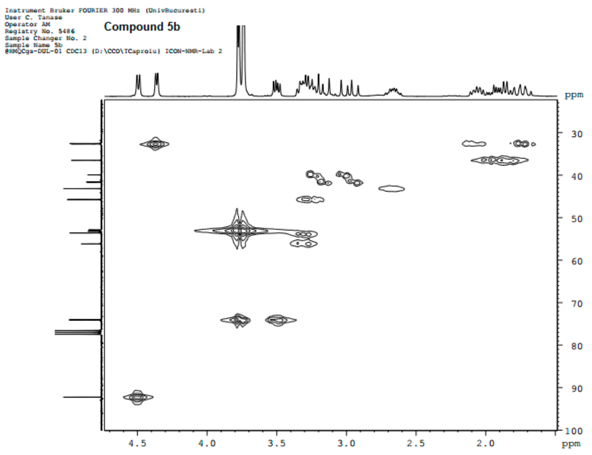

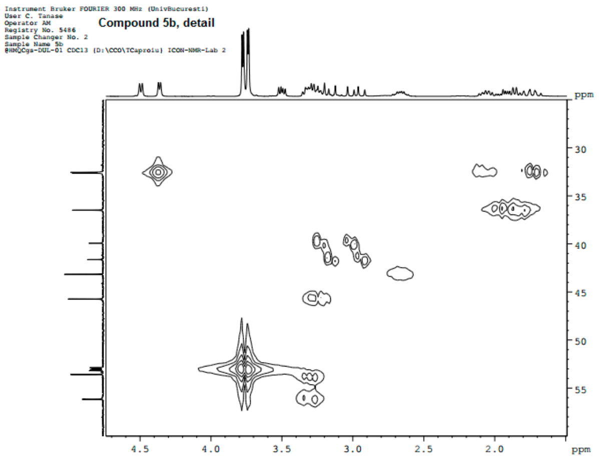

1.9. ^{1}H, ^{13}C, COSY and HETCOR-NMR spectra in $CDCl_3$ of the compound **5c**.

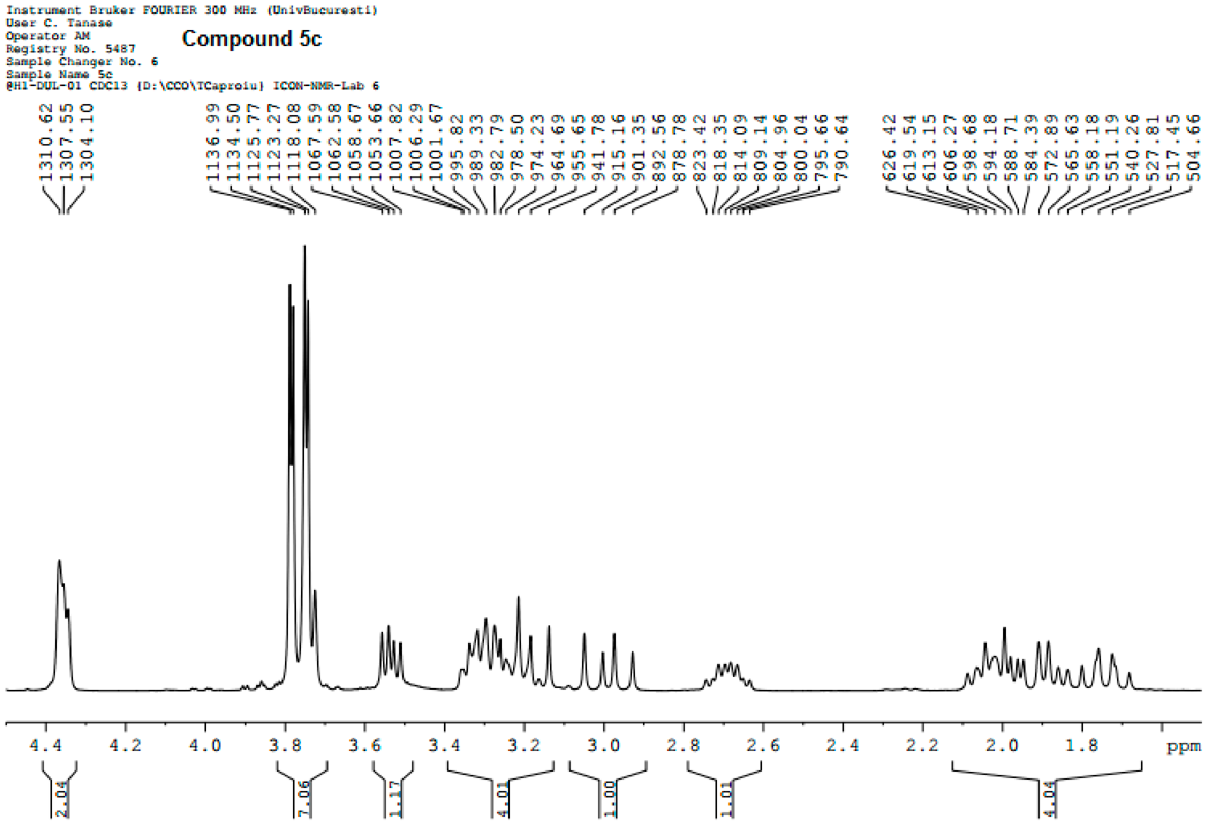

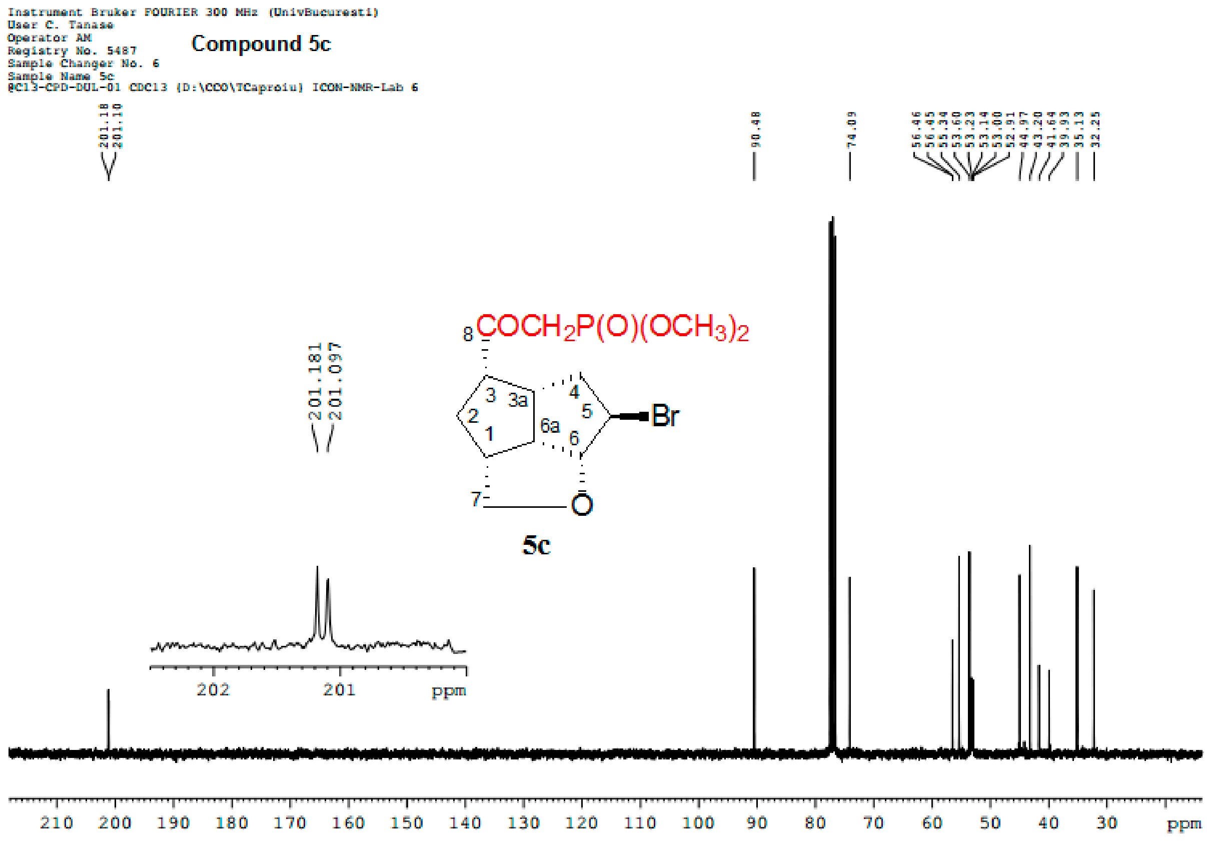

Instrument Bruker FOURIER 300 MHz (UnivBucuresti)
User C. Tanase
Operator AM
Registry No. 5487
Sample Changer No. 6
Sample Name 5c
@C13-CPD-DUL-01 CDCl3 (D:\CCO\TCaproiu) ICON-NMR-Lab 6
Compound 5c
201.18 201.10 90.48 74.09 56.46 56.45 55.34 53.60 53.23 53.14 53.00 52.91 44.97 43.20 41.64 39.93 35.13 32.25
201.181 201.097
8 COCH2P(O)(OCH3)2
Br
5c
ppm

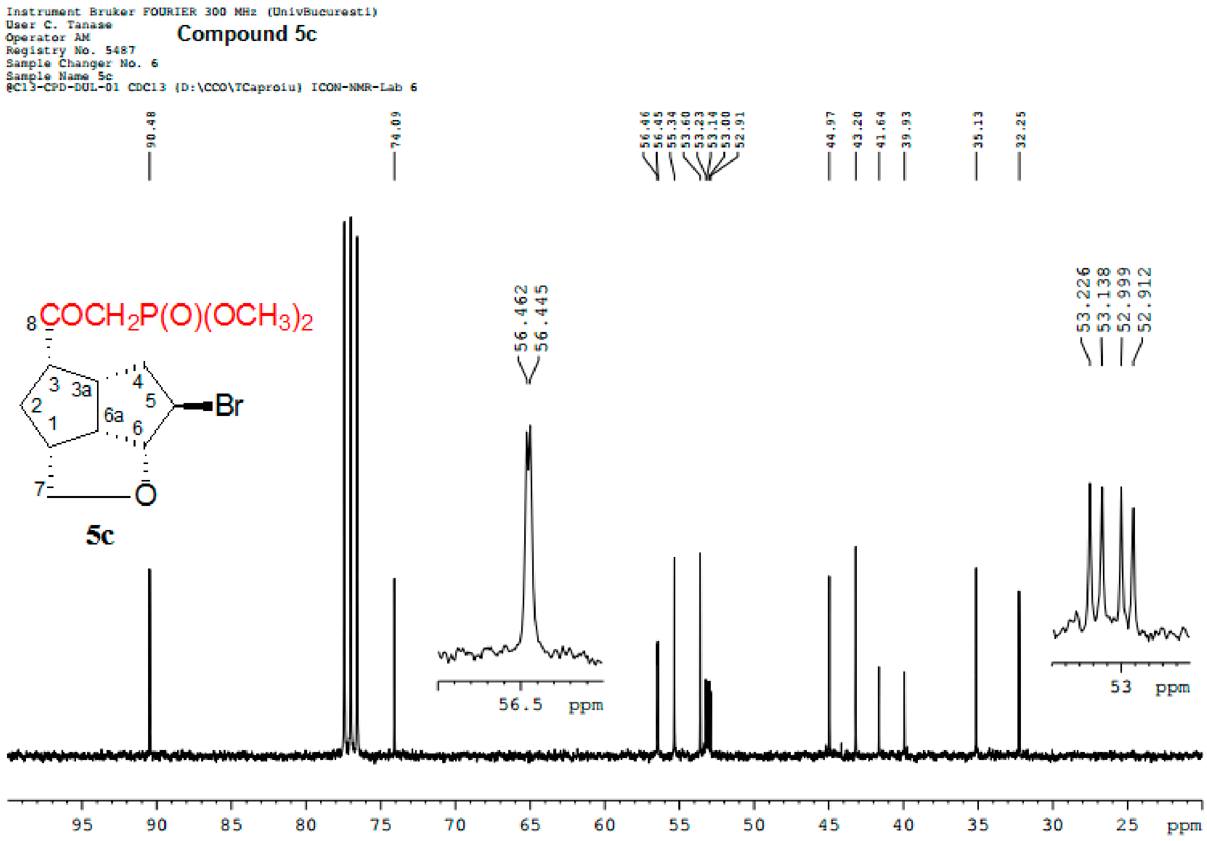

Instrument Bruker FOURIER 300 MHz (UnivBucuresti)
User C. Tanase
Operator AM
Registry No. 5487
Sample Changer No. 6
Sample Name 5c
@C13-CPD-DUL-01 CDCl3 (D:\CCO\TCaproiu) ICON-NMR-Lab 6
Compound 5c
90.48 74.09 56.46 56.45 55.34 53.60 53.23 53.14 53.00 52.91 44.97 43.20 41.64 39.93 35.13 32.25
56.462 56.445
53.226 53.138 52.999 52.912
8 COCH2P(O)(OCH3)2
Br
5c
ppm

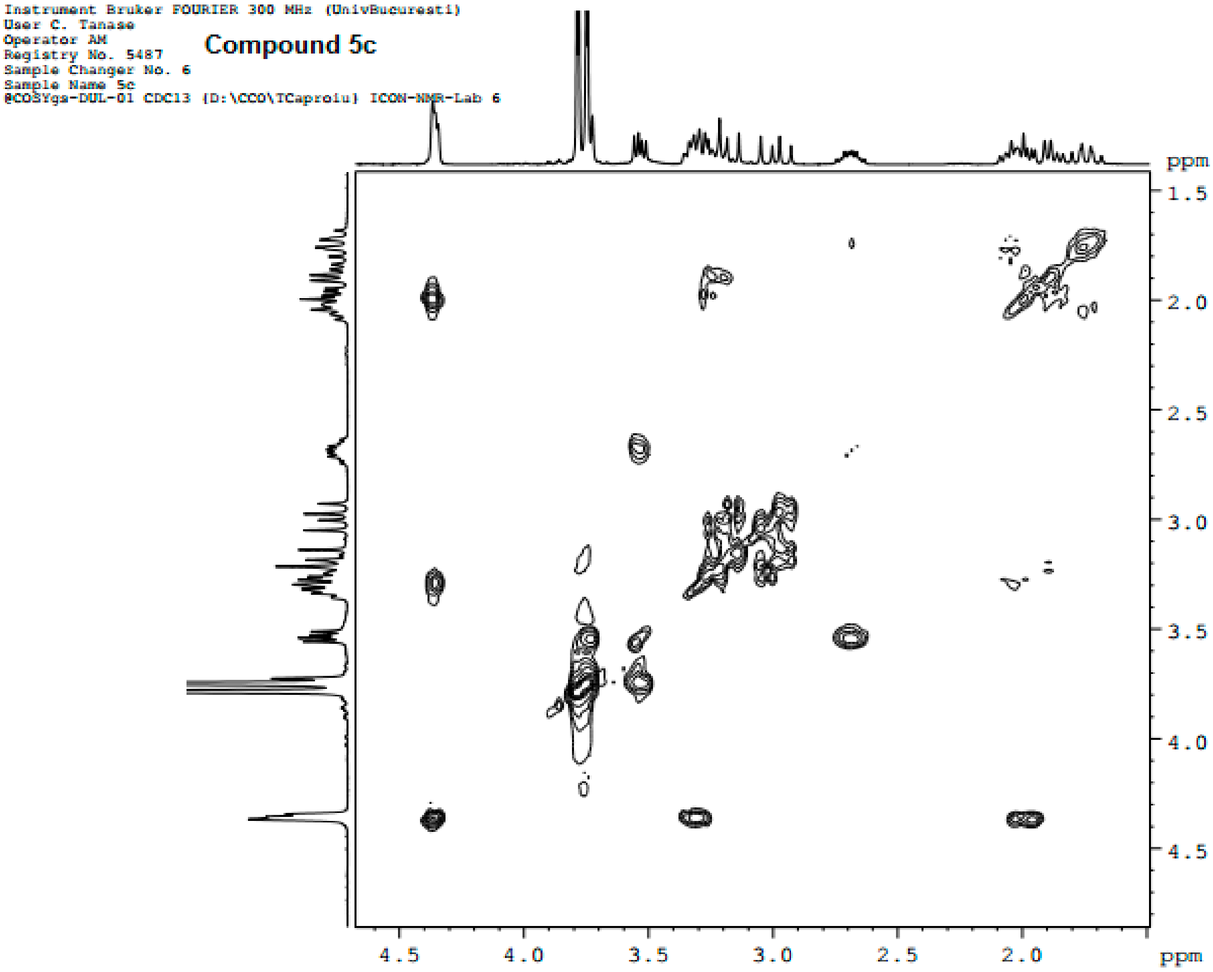

Instrument Bruker FOURIER 300 MHz (UnivBucuresti)
User C. Tanase
Operator AM
Registry No. 5487
Compound 5c
Sample Changer No. 6
Sample Name 5c
@COSYgs-DUL-01 CDCl3 (D:\CCO\TCaproiu) ICON-NMR-Lab 6
ppm
1.5
2.0
2.5
3.0
3.5
4.0
4.5
4.5
4.0
3.5
3.0
2.5
2.0
ppm

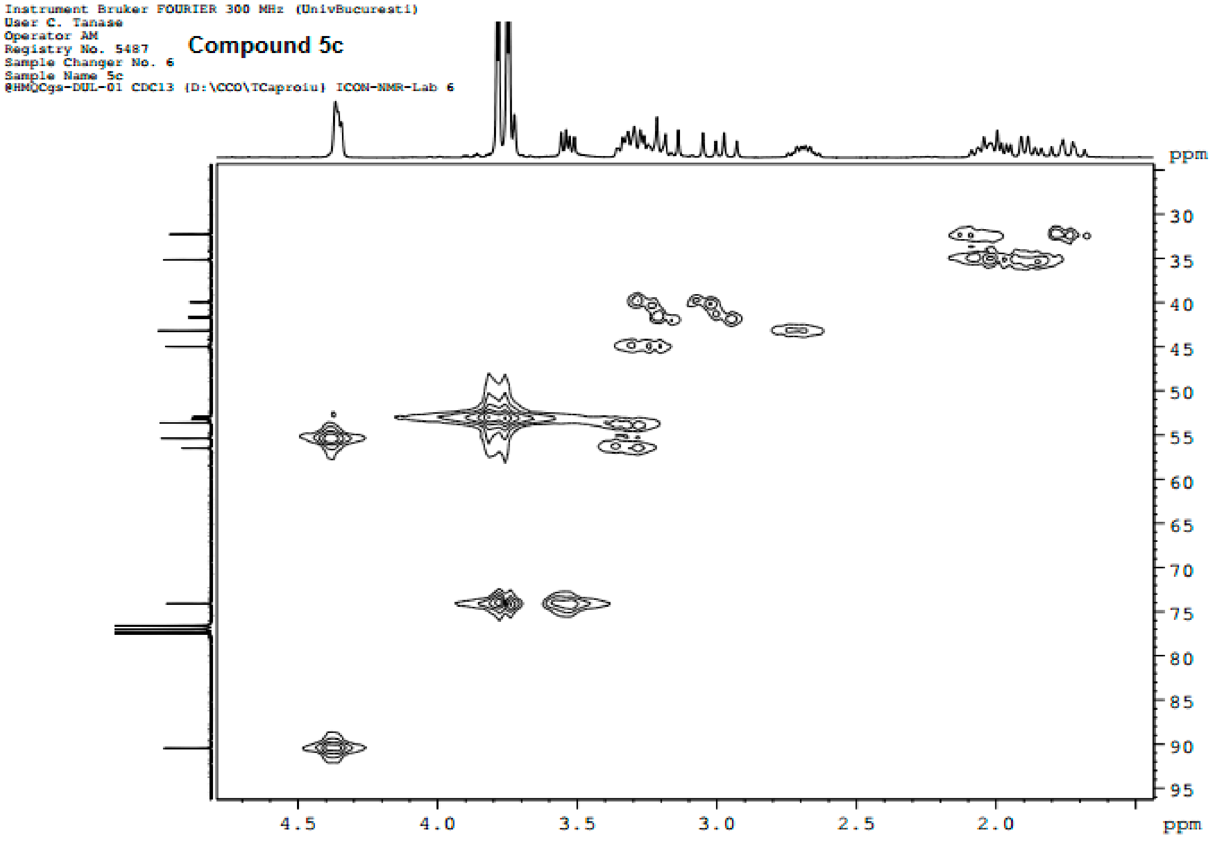

Instrument Bruker FOURIER 300 MHz (UnivBucuresti)
User C. Tanase
Operator AM
Registry No. 5487
Compound 5c
Sample Changer No. 6
Sample Name 5c
@HMQCgs-DUL-01 CDCl3 (D:\CCO\TCaproiu) ICON-NMR-Lab 6
ppm
30
35
40
45
50
55
60
65
70
75
80
85
90
95
4.5
4.0
3.5
3.0
2.5
2.0
ppm

1.10. ^{1}H and ^{13}C-NMR spectra in $CDCl_3$ of the compound **6b**.

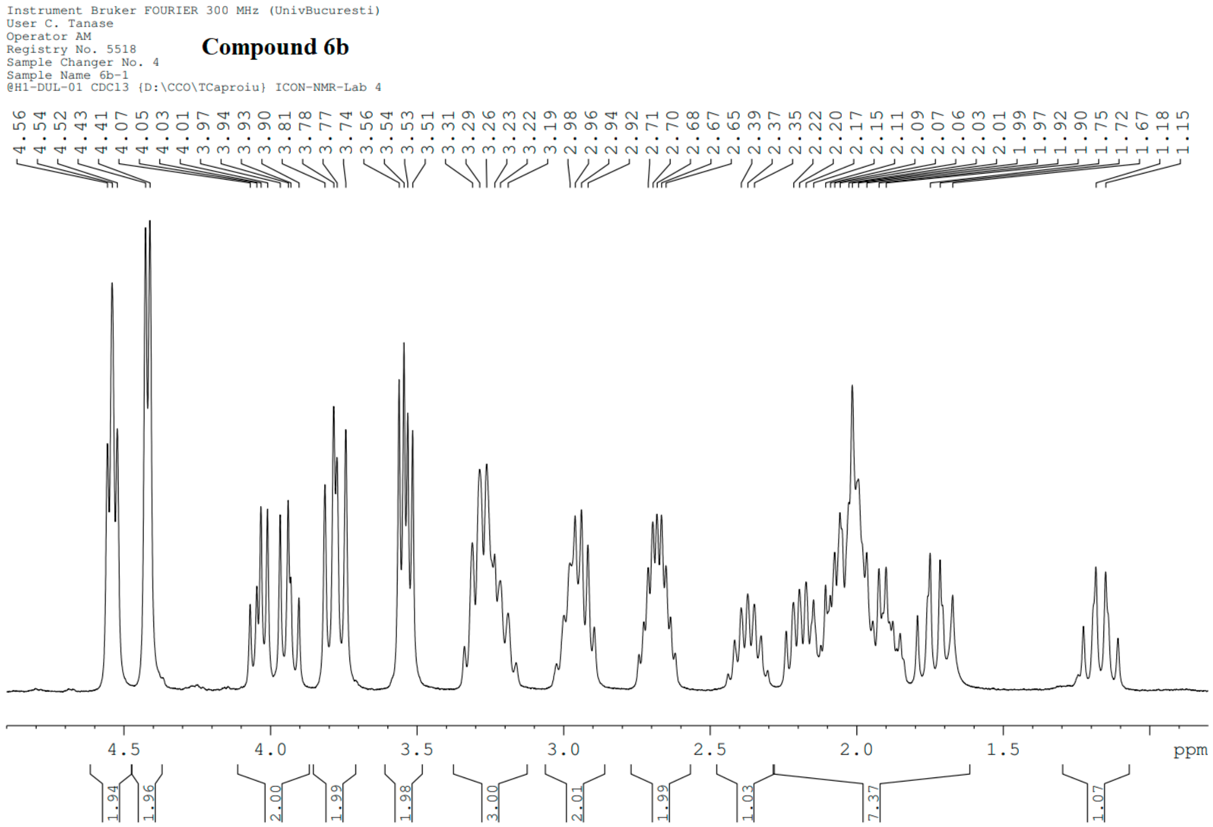

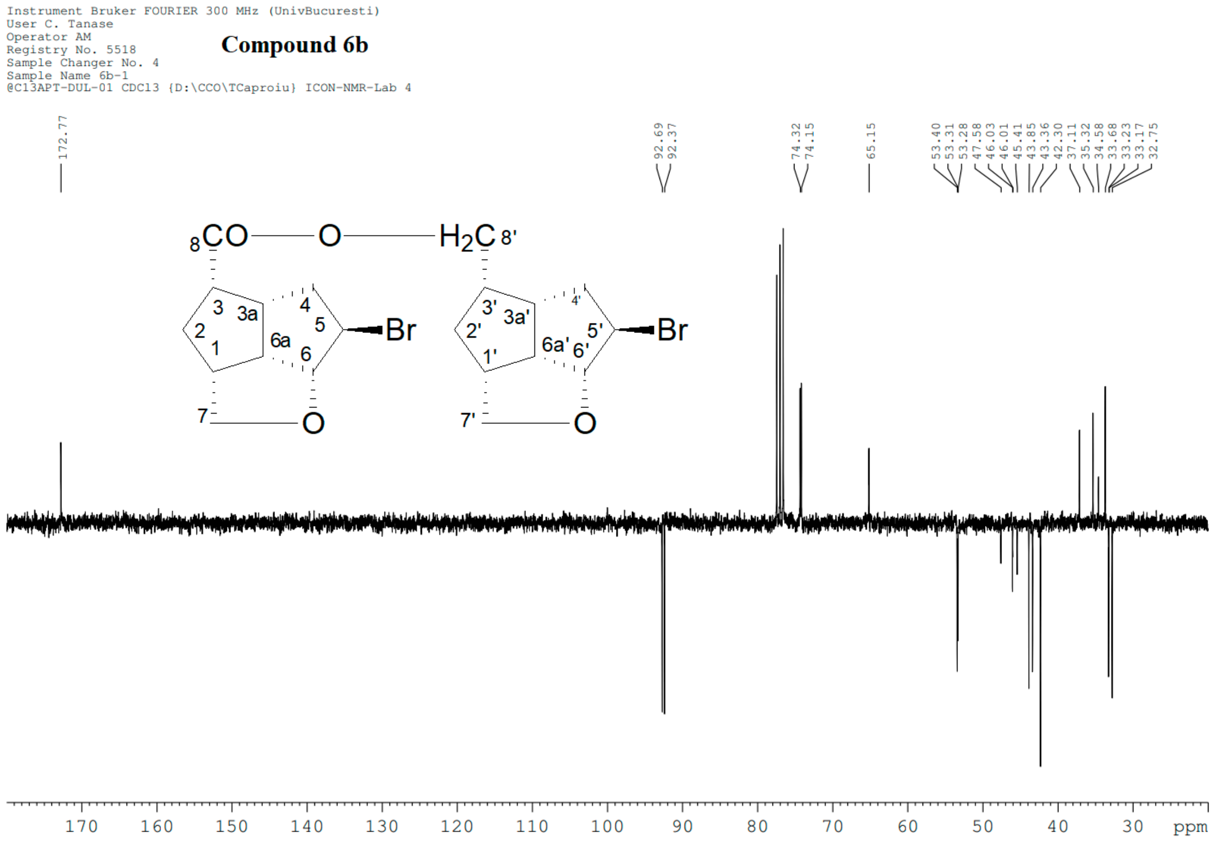

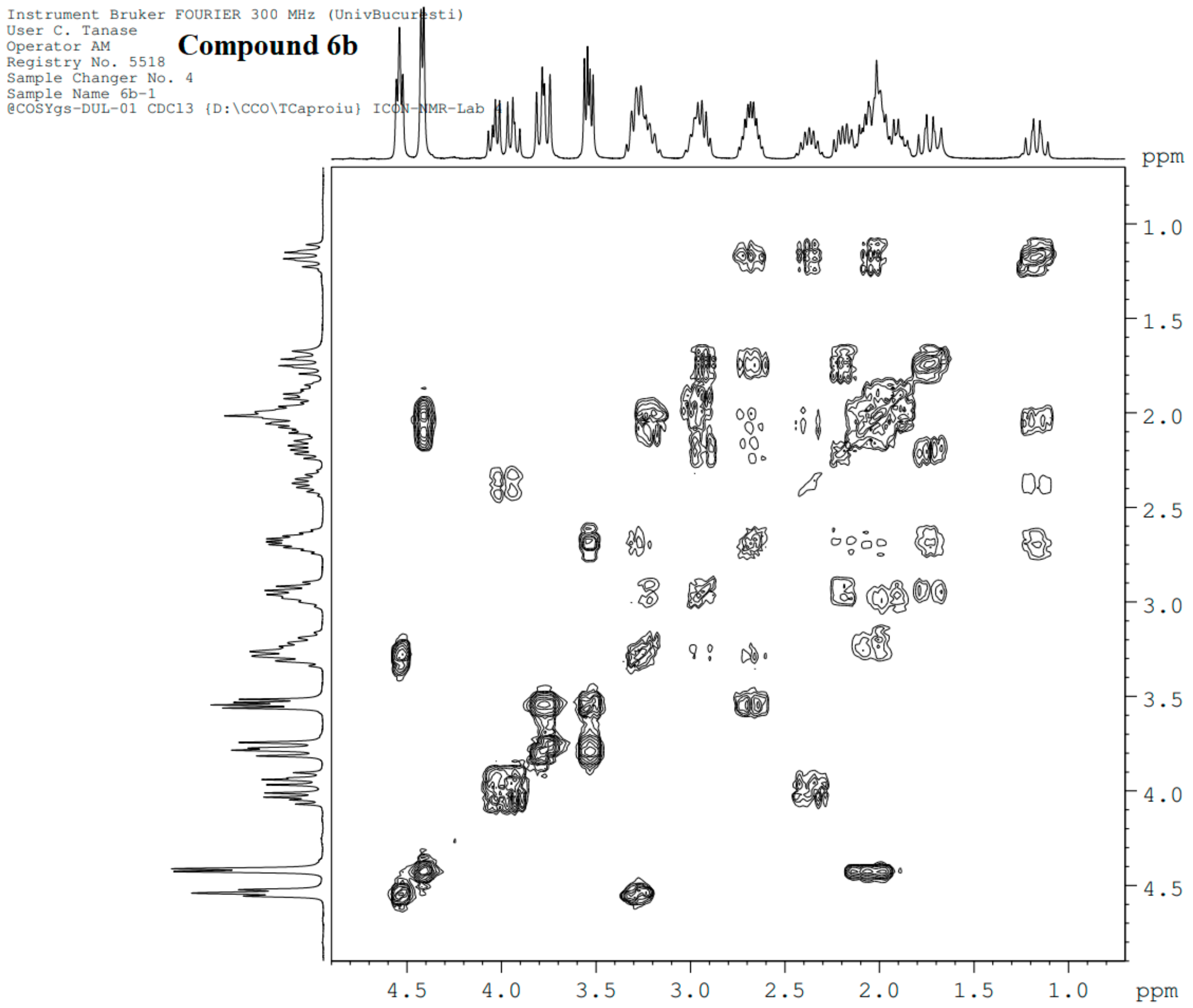
Instrument Bruker FOURIER 300 MHz (UnivBucuresti)
User C. Tanase
Operator AM
Registry No. 5518
Sample Changer No. 4
Sample Name 6b-1
@COSYgs-DUL-01 CDCl3 {D:\CCO\TCaproiu} ICON-NMR-Lab 4
Compound 6b
ppm

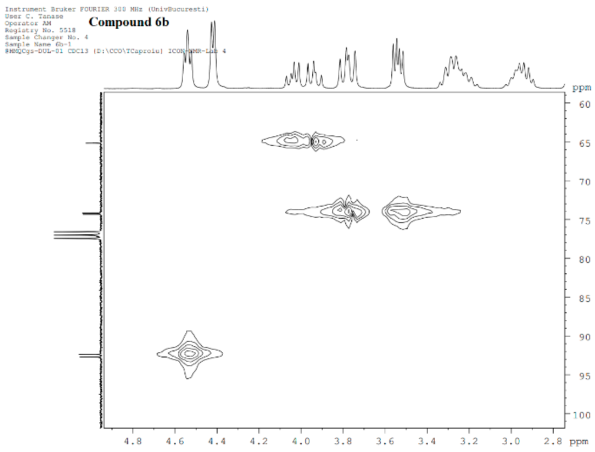
Compound 6b
ppm

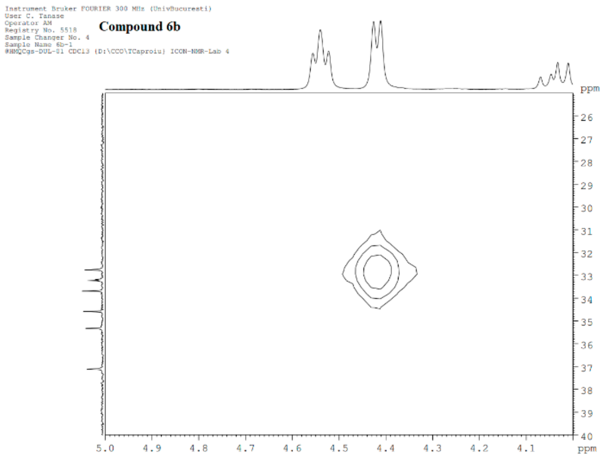
Compound 6b
ppm

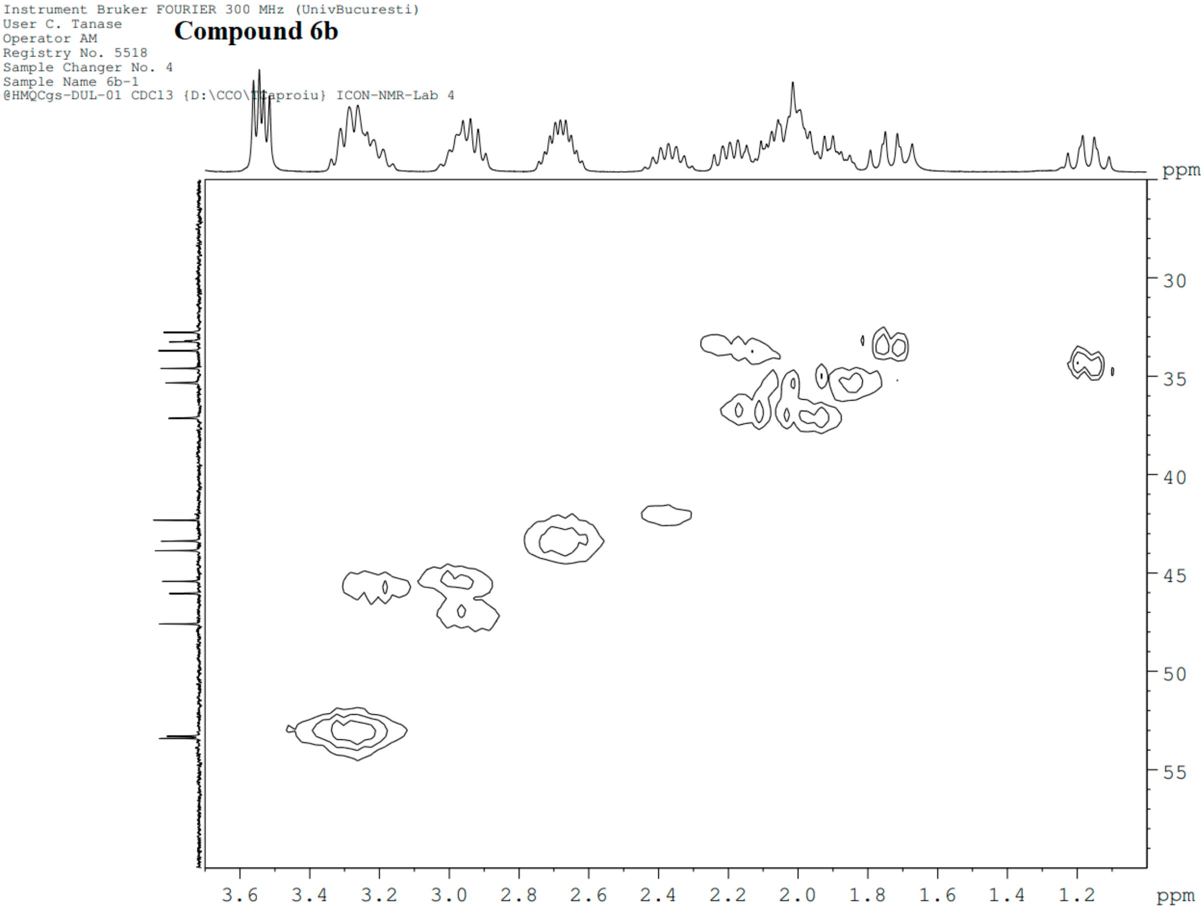

1.11. ^{1}H,^{13}C, COSY and HETCOR-NMR spectra in $CDCl_3$ of the compound **6c**.

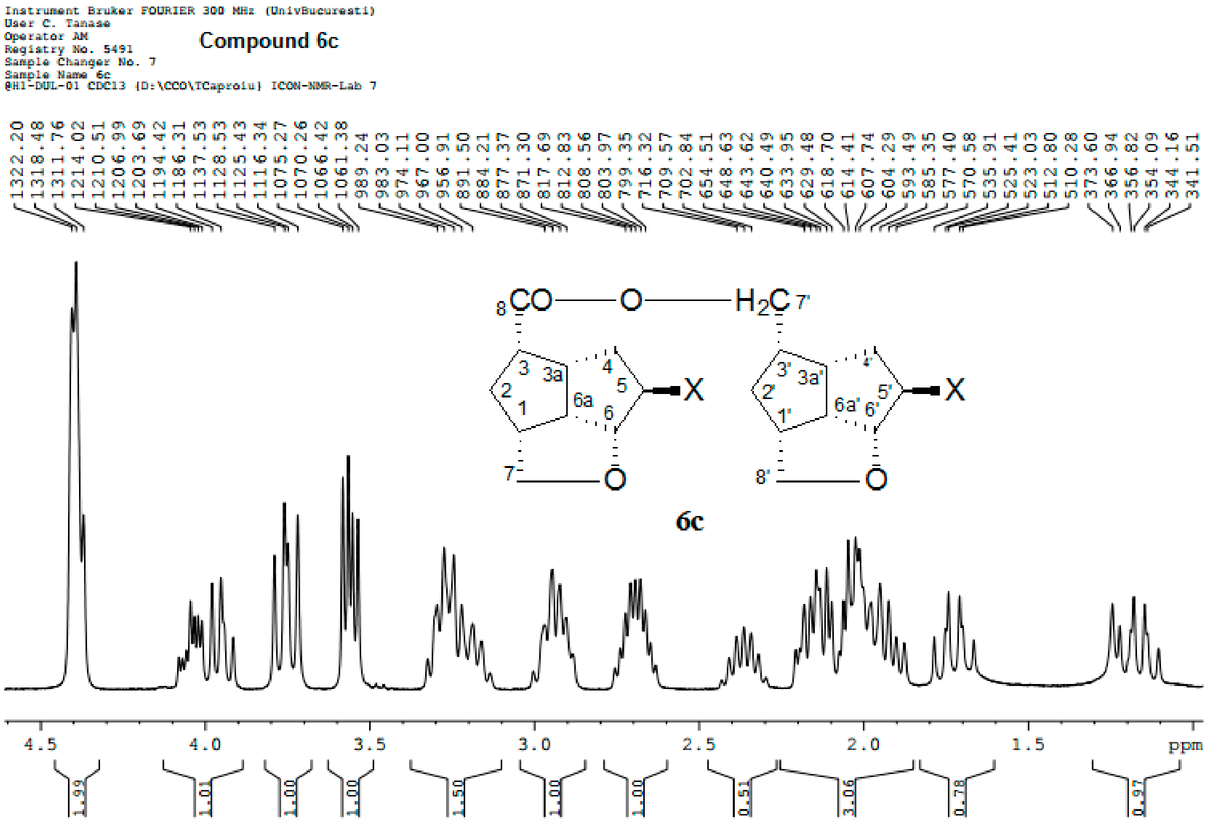

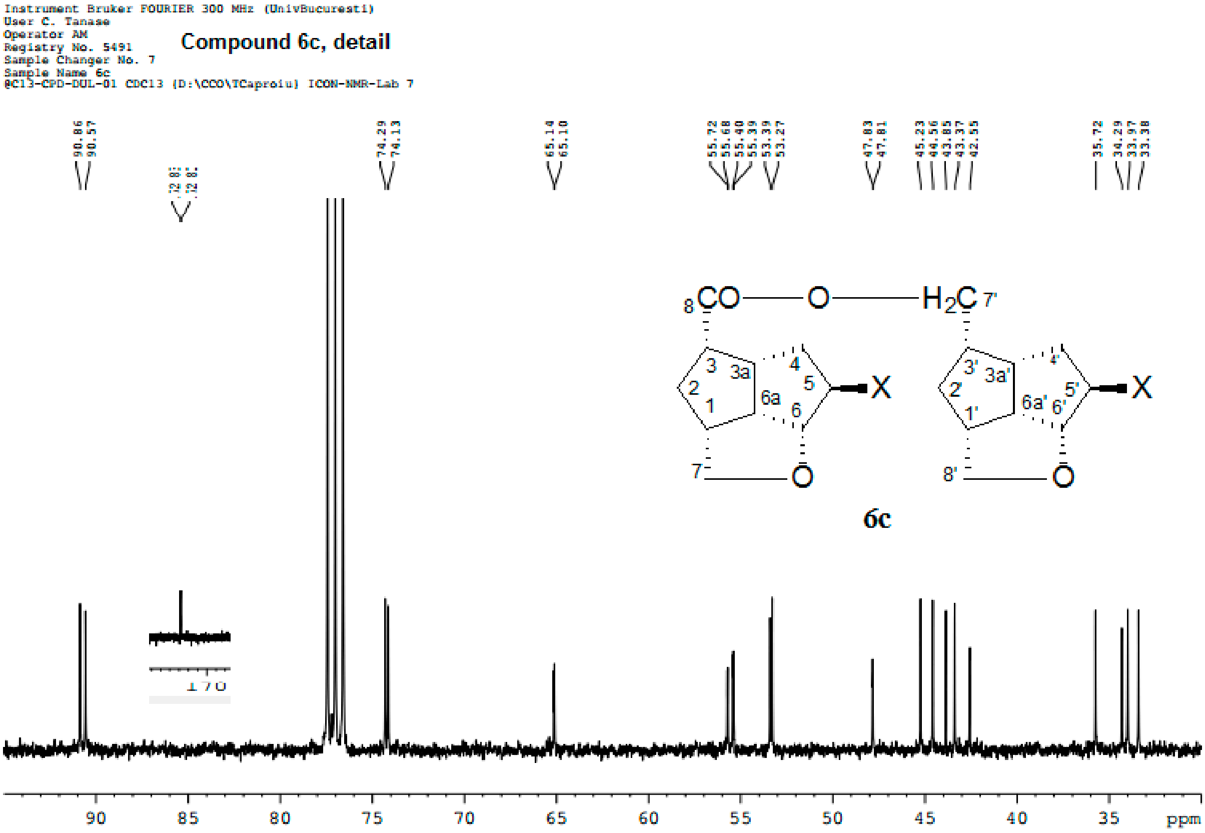
Instrument Bruker FOURIER 300 MHz (UnivBucuresti)
User C. Tanase
Operator AM
Registry No. 5491
Sample Changer No. 7
Sample Name 6c
@C13-CPD-DUL-01 CDC13 (D:\CCO\TCaproiu) ICON-NMR-Lab 7
Compound 6c, detail
6c
ppm

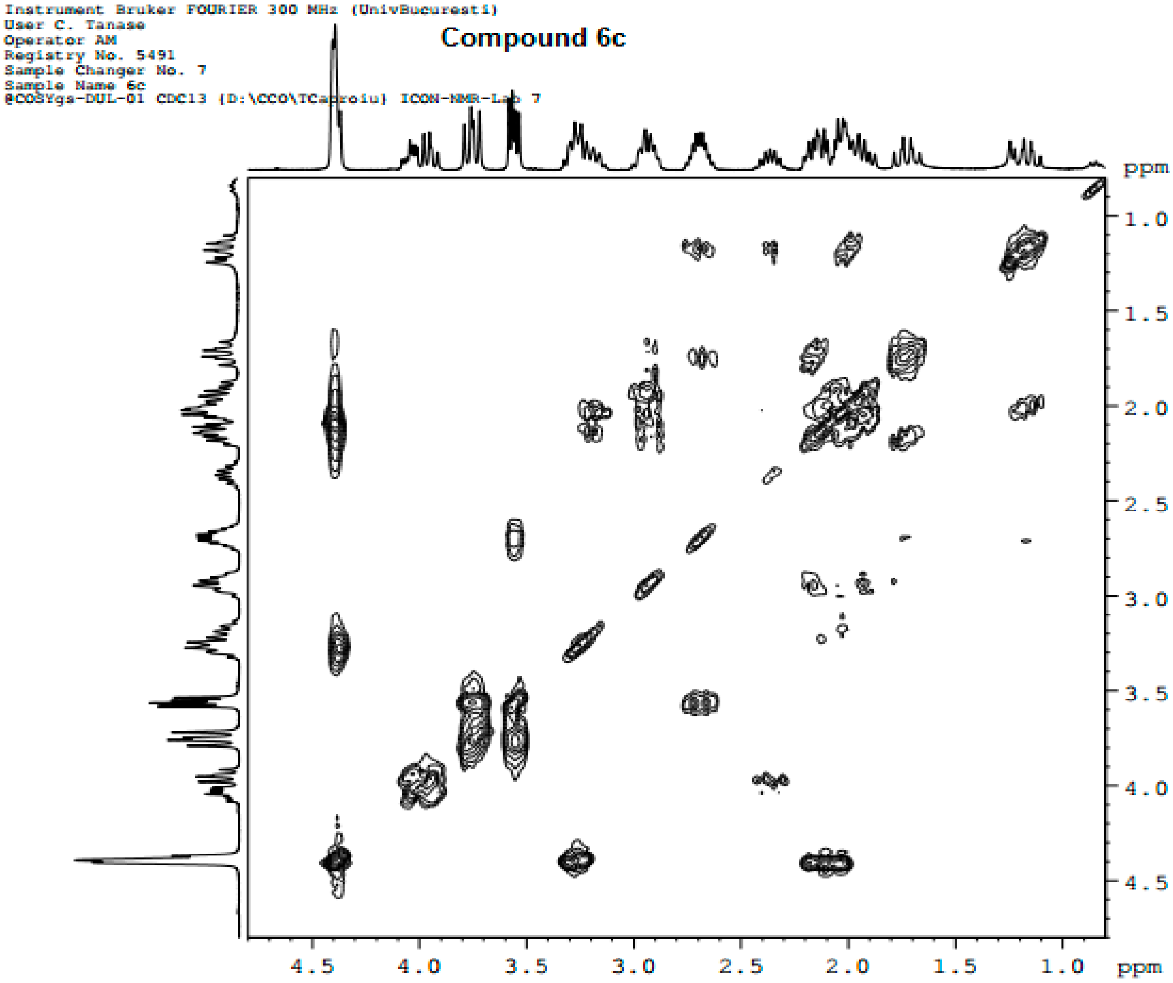
Instrument Bruker FOURIER 300 MHz (UnivBucuresti)
User C. Tanase
Operator AM
Registry No. 5491
Sample Changer No. 7
Sample Name 6c
@COSYgs-DUL-01 CDC13 (D:\CCO\TCaproiu) ICON-NMR-Lab 7
Compound 6c
ppm

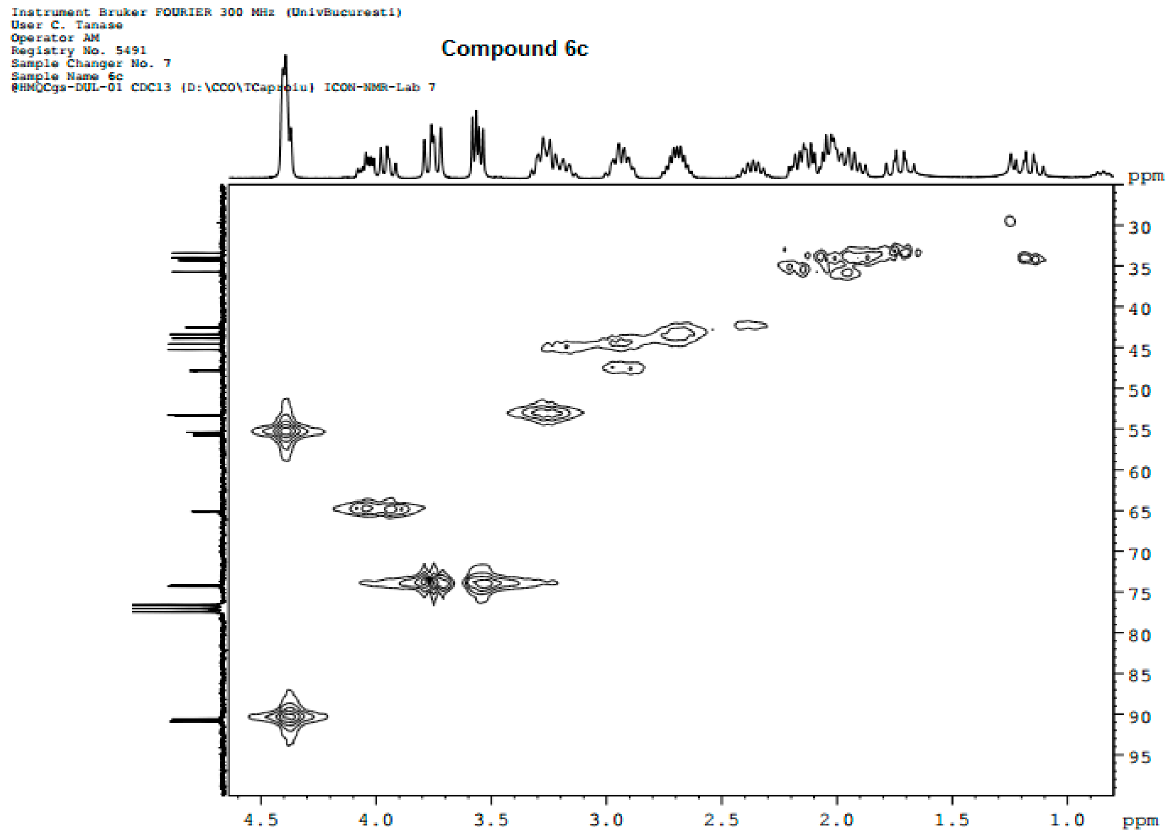

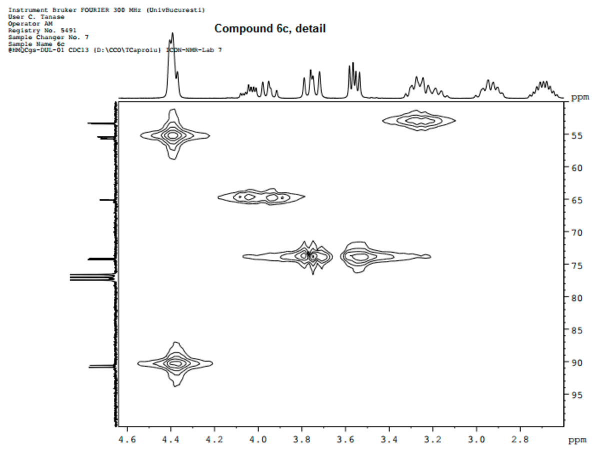

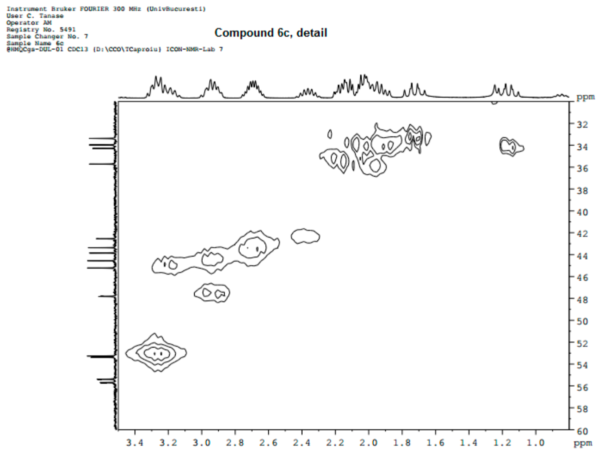

FT-IR Spectra for compounds 3b, 3c, 4c,5b and 5c:

3b. FT-IR (cm^{-1}): 3018m, 2959m, 2915m, 2851m, 2641br, 2150w, 2044w, 1891w, 1685vs,1419m, 1345m, 1249m, 1210m, 1109m, 1058s, 906s, 802w

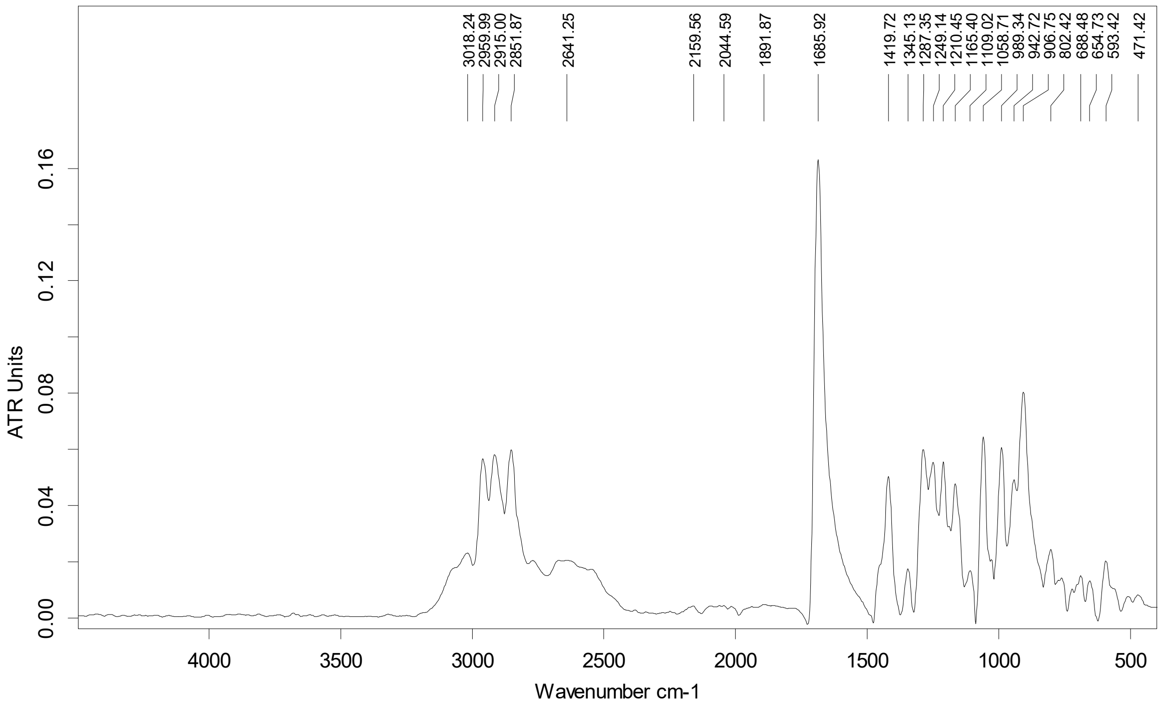

\\unity\ftir\Maria\TANASE\3b.0 3b SOLID IN ATR	27/10/2020

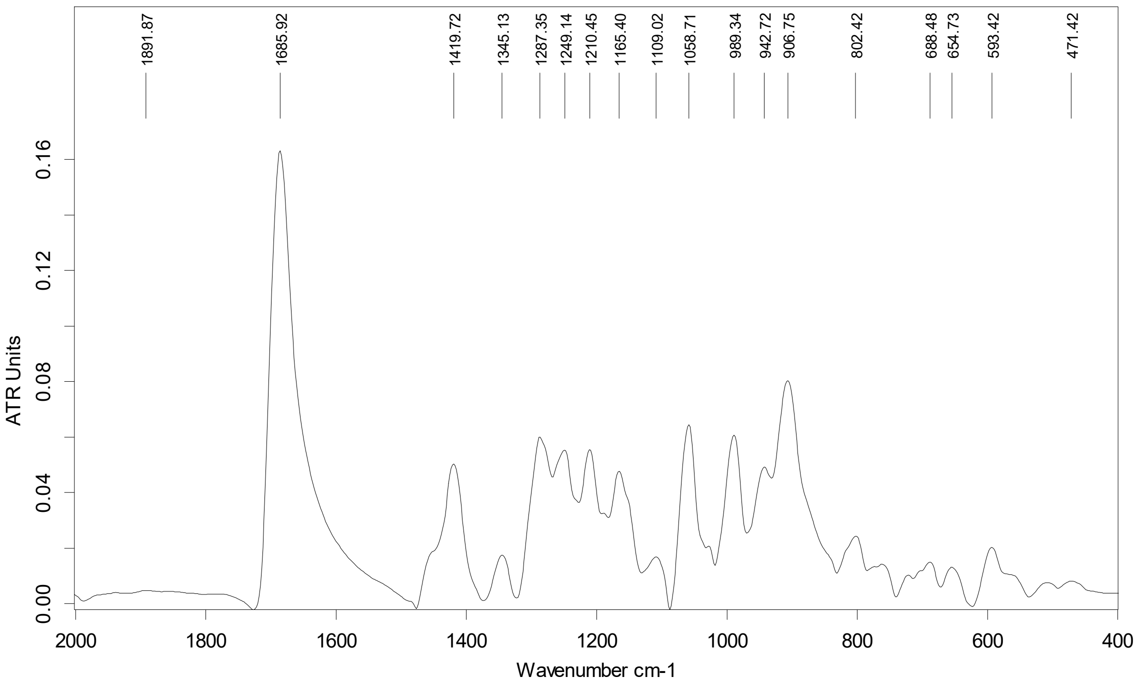

\\unity\ftir\Maria\TANASE\3b.0 3b SOLID IN ATR	27/10/2020

3c. FT-IR (cm-1):3029w, 2964m, 2922m, 2863m, 2684br, 1865w, 1690vs, 1430m, 1349m, 1287m, 1213m, 1065m, 995m, 910m, 804w

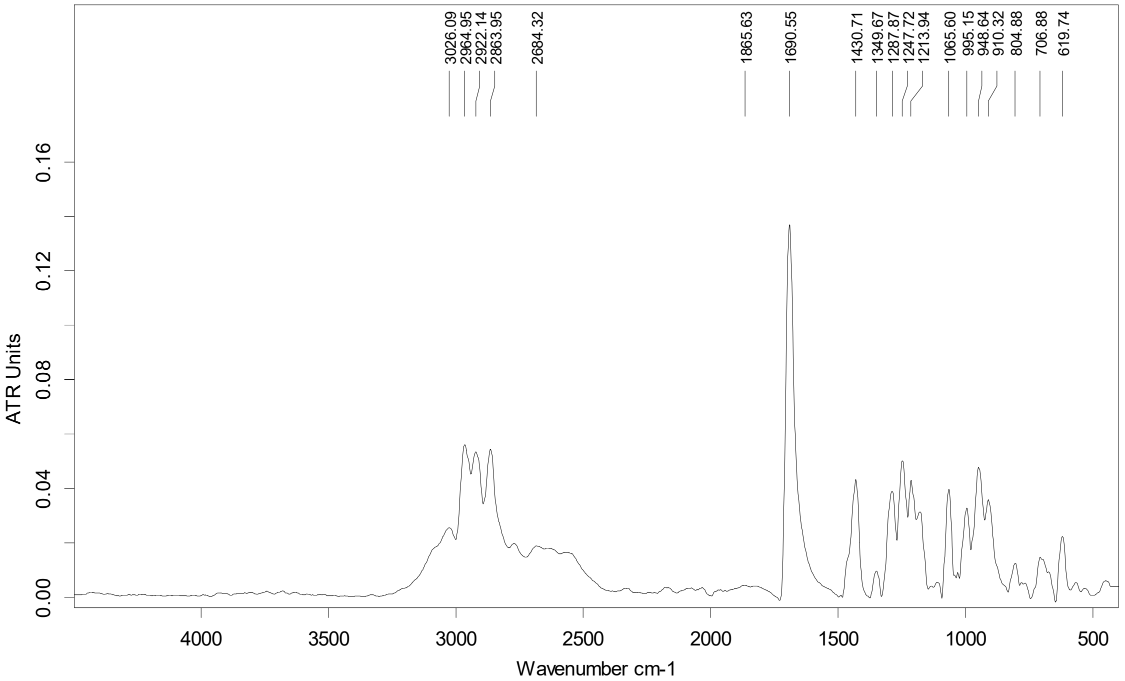

\\unity\ftir\Maria\TANASE\3c.0 3c SOLID IN ATR 27/10/2020

Page 1 of 1

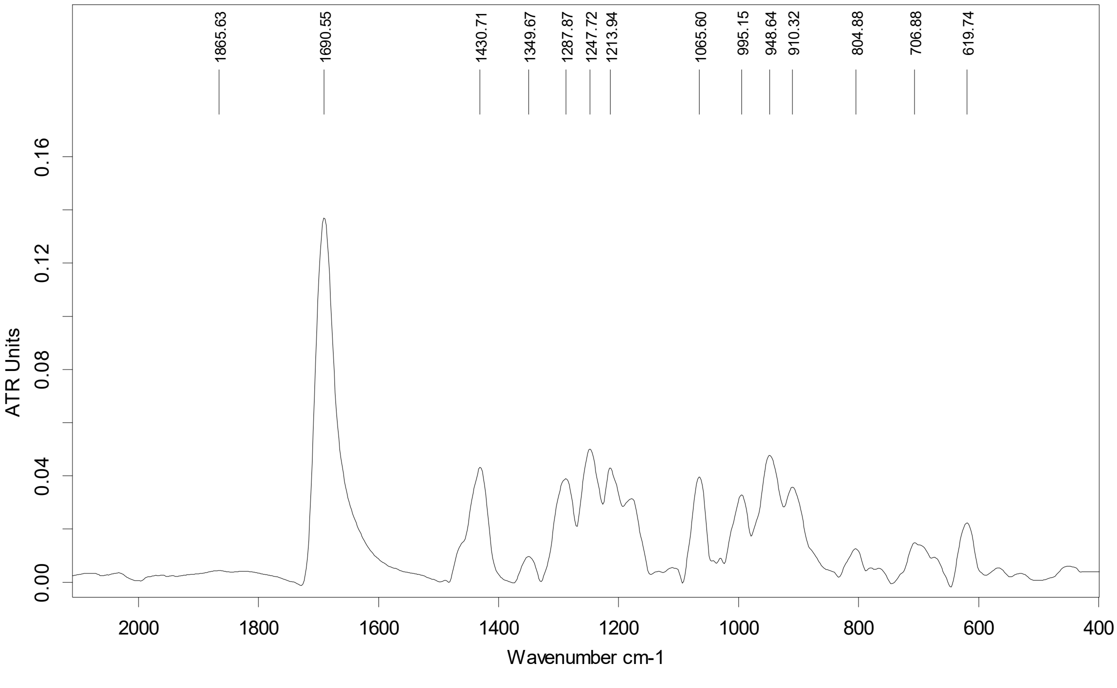

\\unity\ftir\Maria\TANASE\3c.0 3c SOLID IN ATR 27/10/2020

Page 1 of 1

4c, FT-IR (cm[-1]):3443w, 2936s, 2842s, 2693w, 2174w, 2070w, 1791w, 1723vs, 1432m, 1378m, 1282m, 1193m.1163vs, 1058m, 1003m, 916m, 801m

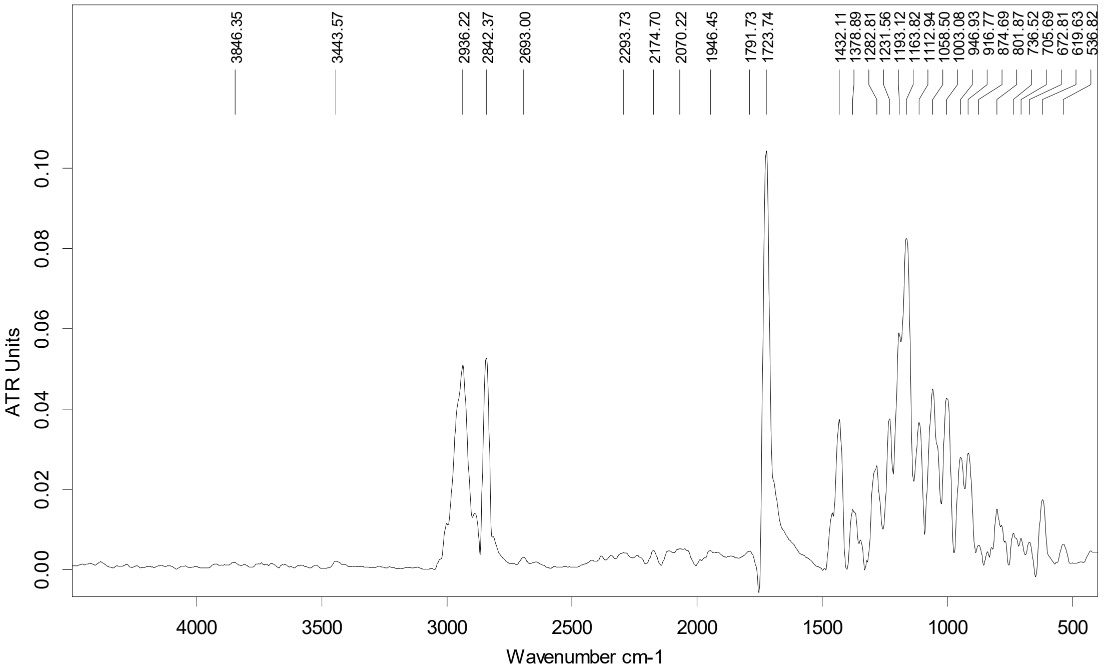

\\unity\ftir\Maria\TANASE\4c.0	4c	SOLID IN ATR		27/10/2020

Page 1 of 1

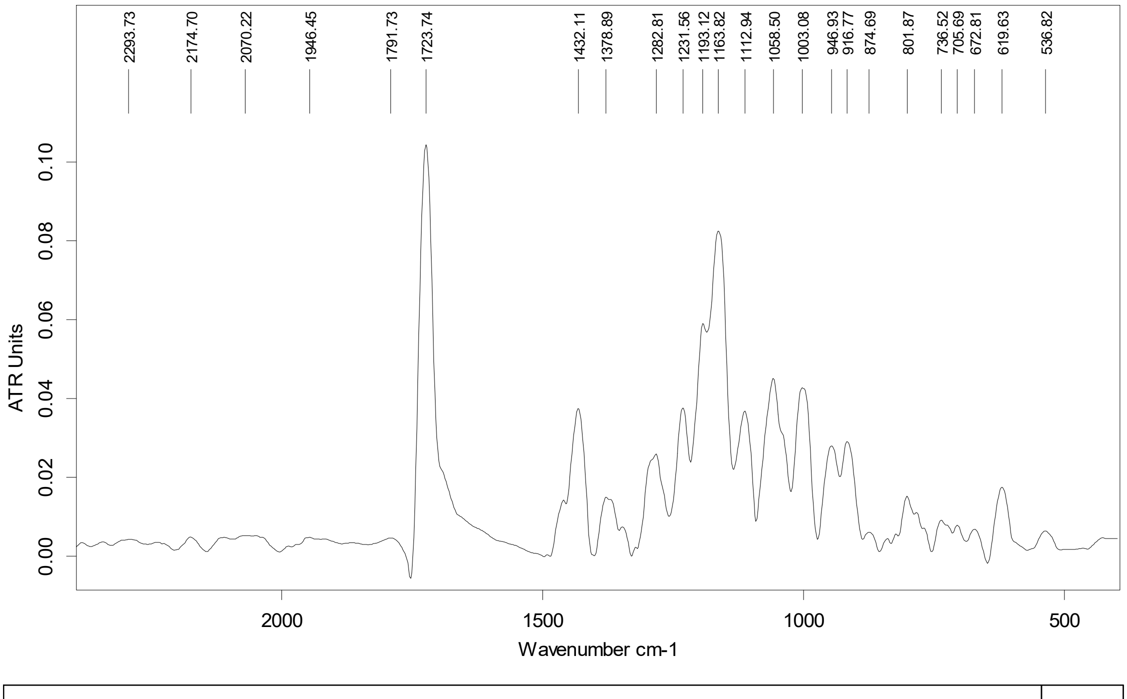

\\unity\ftir\Maria\TANASE\4c.0	4c	SOLID IN ATR		27/10/2020

Page 1 of 1

5b, FT-IR (cm^{-1}): 3384w, 2951s, 2905m, 2850m, 2081w, 1835w, 1696s, 1460w, 1376w, 1291m, 1245s, 1187m, 1167m, 1024vs, 957m, 806s

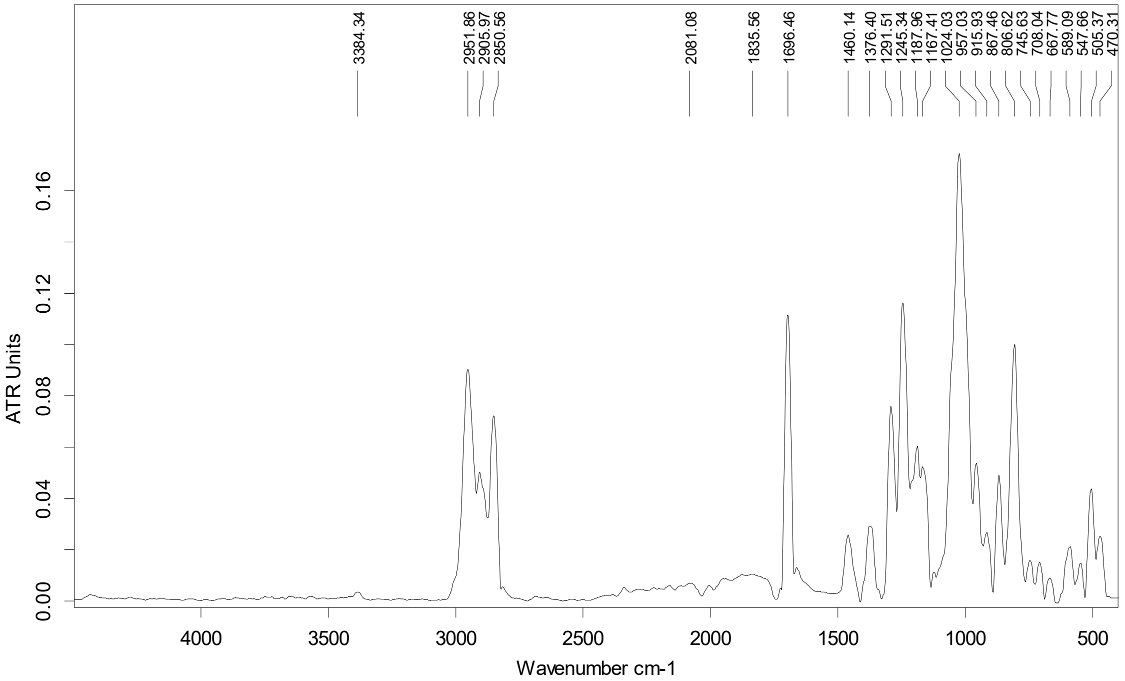

\\unity\ftir\Maria\TANASE\5b.0 5b SOLID IN ATR 27/10/2020

Page 1 of 1

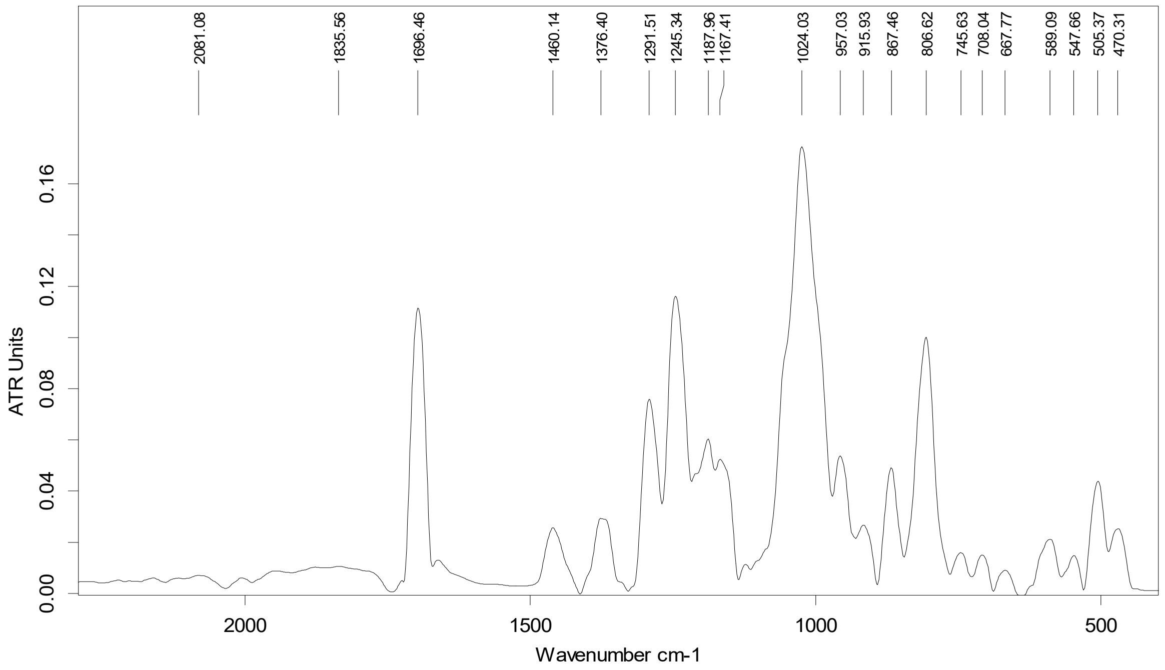

\\unity\ftir\Maria\TANASE\5b.0 5b SOLID IN ATR 27/10/2020

Page 1 of 1

5c, FT-IR (cm^{-1}): 3380w, 2948m, 2899m, 2856m, 2070w, 1853w, 1696s, 1456w, 1364w, 1289m, 1211s, 1186m, 1019vs, 953w, 807s

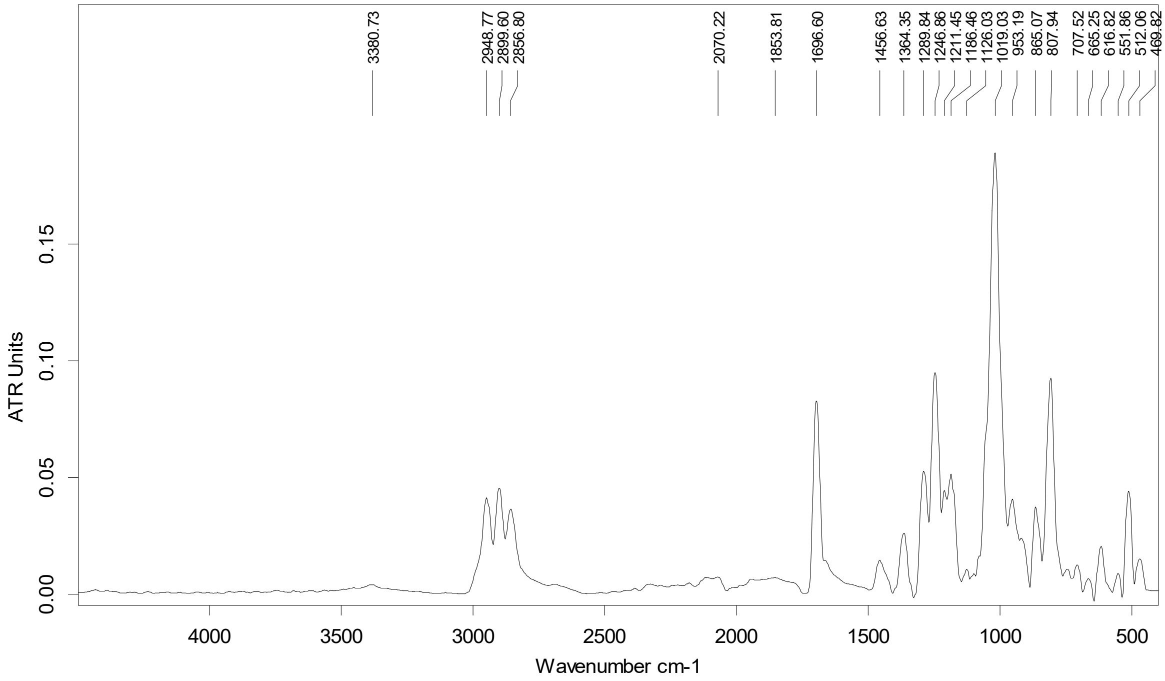

\\unity\ftir\Maria\TANASE\5c.0 5c SOLID IN ATR 27/10/2020

Page 1 of 1

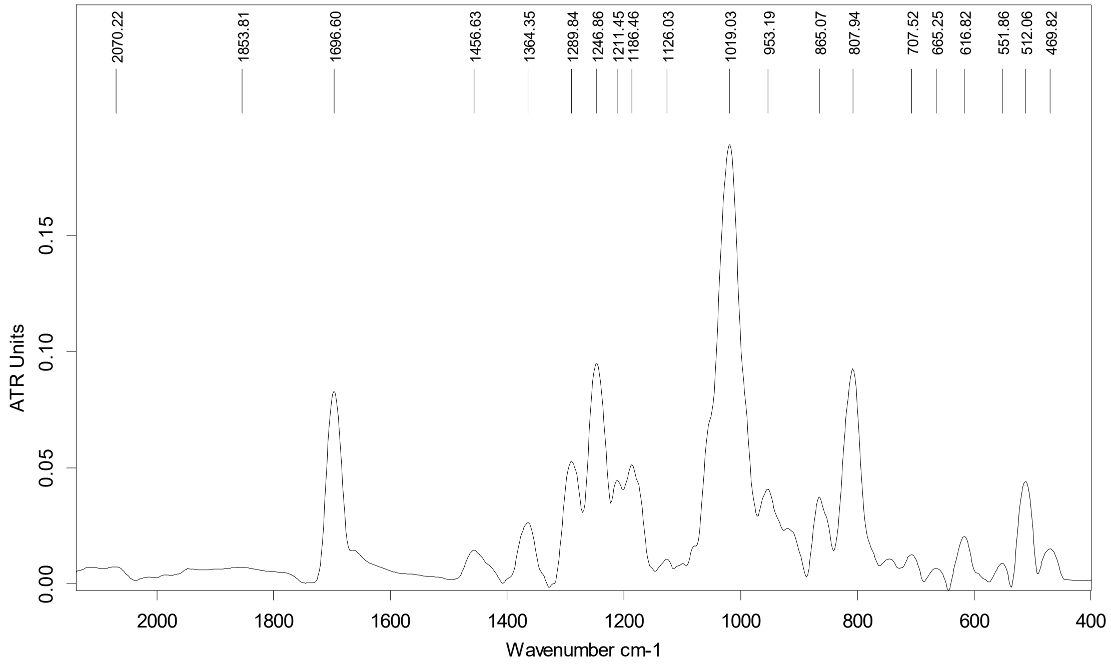

\\unity\ftir\Maria\TANASE\5c.0 5c SOLID IN ATR 27/10/2020

Page 1 of 1

6c, FT-IR (cm^{-1}): 3624w, 3055w, 2927s, 2852s, 2186w, 2083w, 1796e, 1711vs, 1460w, 1399m, 1288m, 1178vs, 1060s, 999s, 912m, 618m

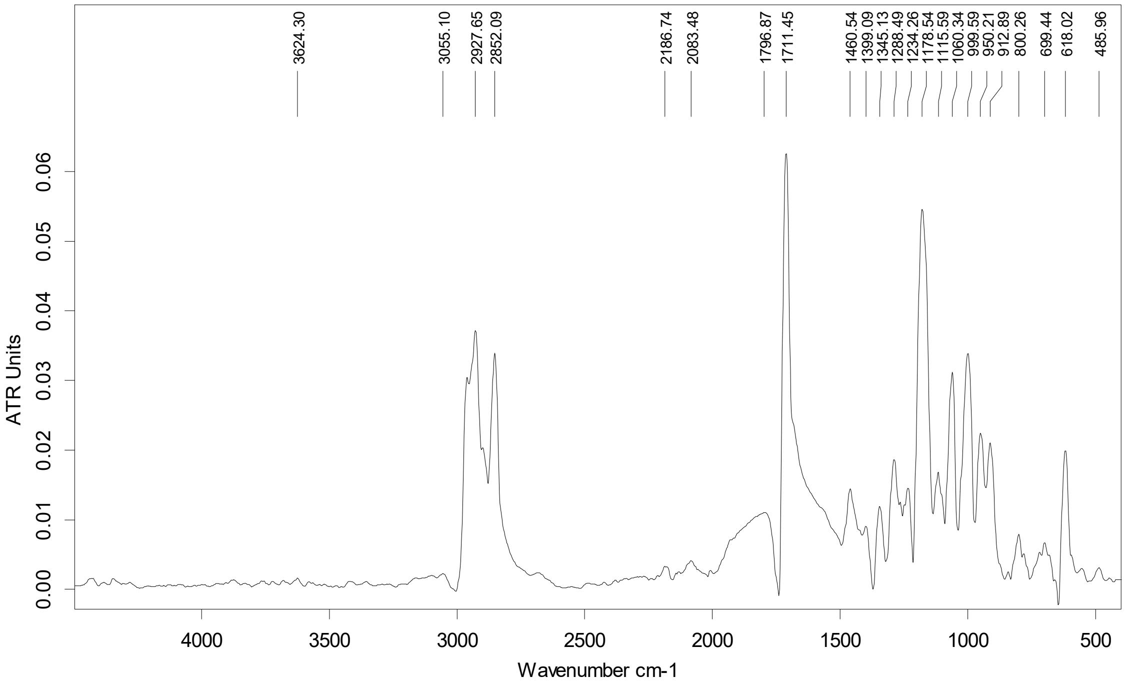

\\unity\ftir\Maria\TANASE\6c.0 6c SOLID IN ATR	27/10/2020

Page 1 of 1

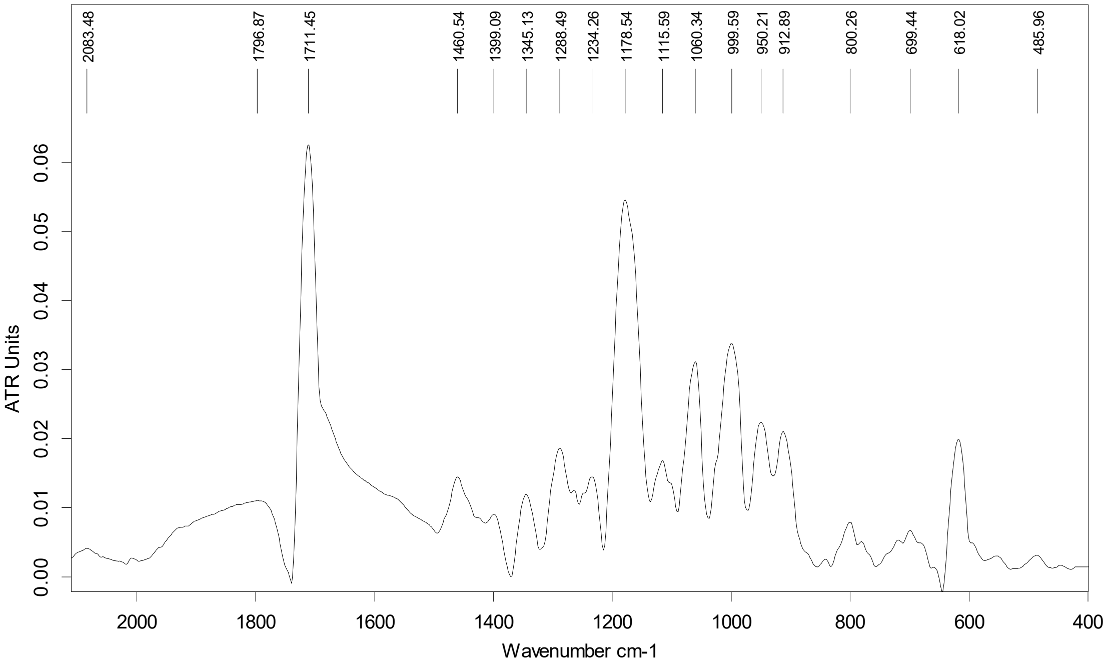

\\unity\ftir\Maria\TANASE\6c.0 6c SOLID IN ATR	27/10/2020

Page 1 of 1

3. HR-MS spectra for compounds 3a-3c, 4a-4c, 6b-6c, 5b and 5c.

3.1. HR-MS spectrum for the compound **3a**, **3b** and **3c**.

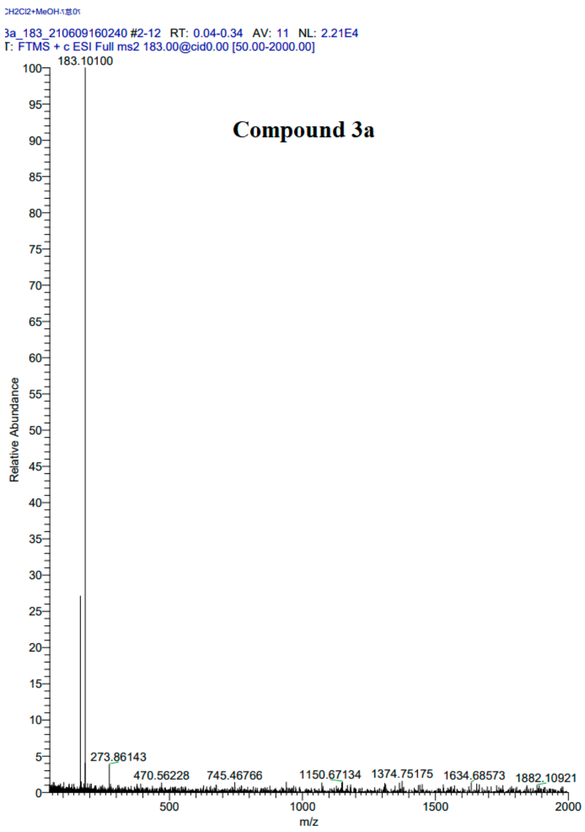

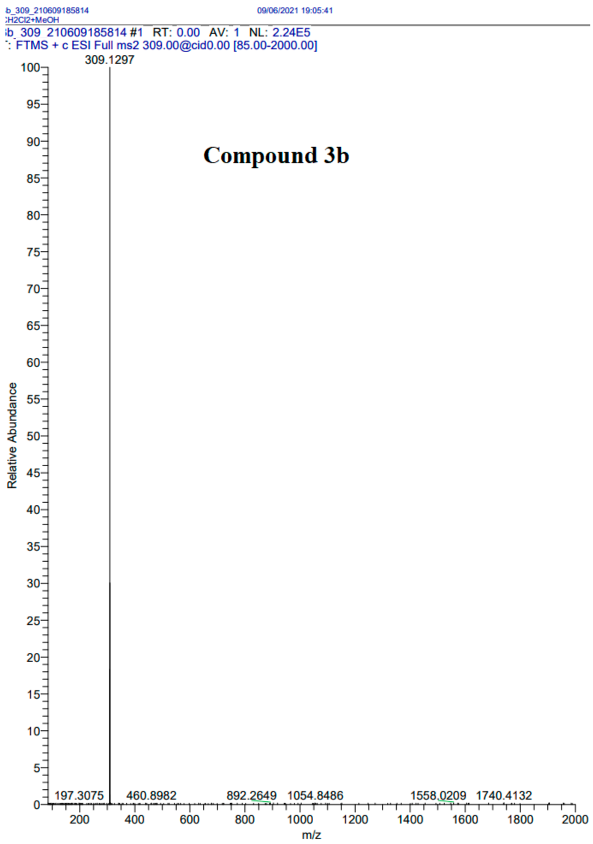

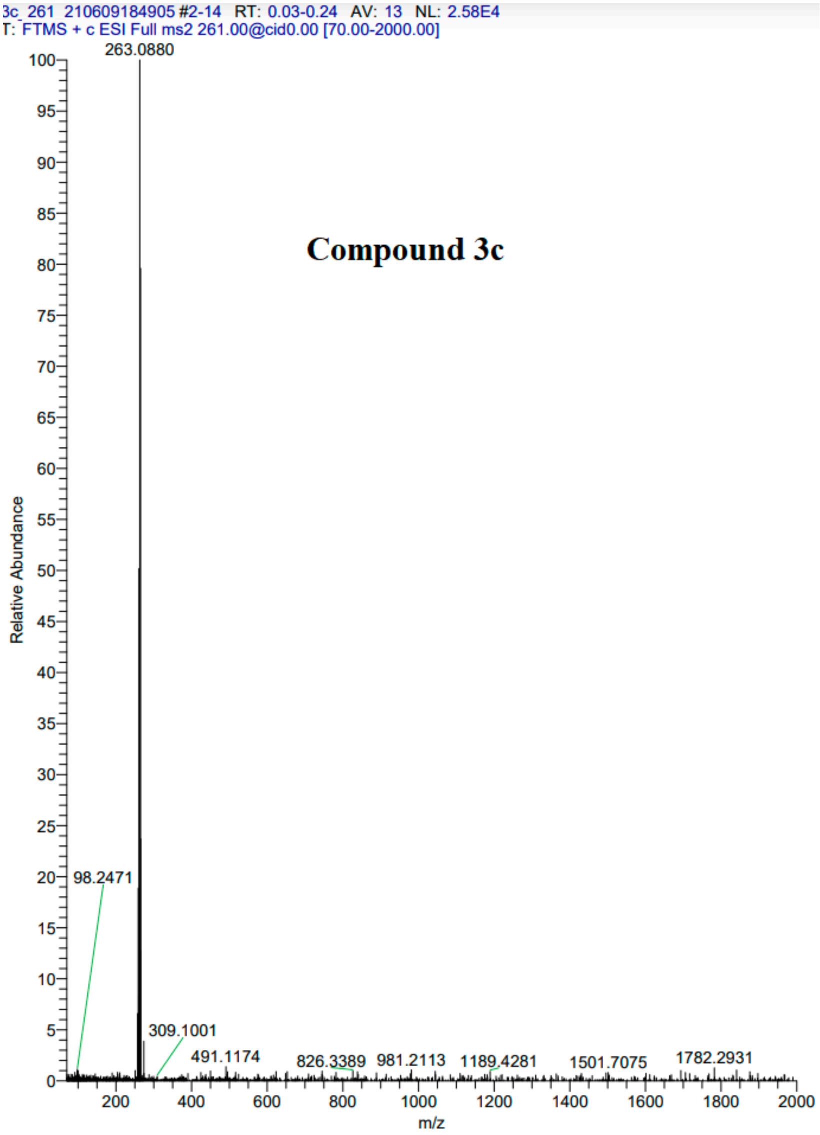

3c_261 210609184905 #2-14 RT: 0.03-0.24 AV: 13 NL: 2.58E4
T: FTMS + c ESI Full ms2 261.00@cid0.00 [70.00-2000.00]
263.0880
Compound 3c
Relative Abundance
98.2471
309.1001
491.1174
826.3389
981.2113
1189.4281
1501.7075
1782.2931
m/z

3.2. HR-MS spectrum for the compound **4a**, **4b** and **4c**.

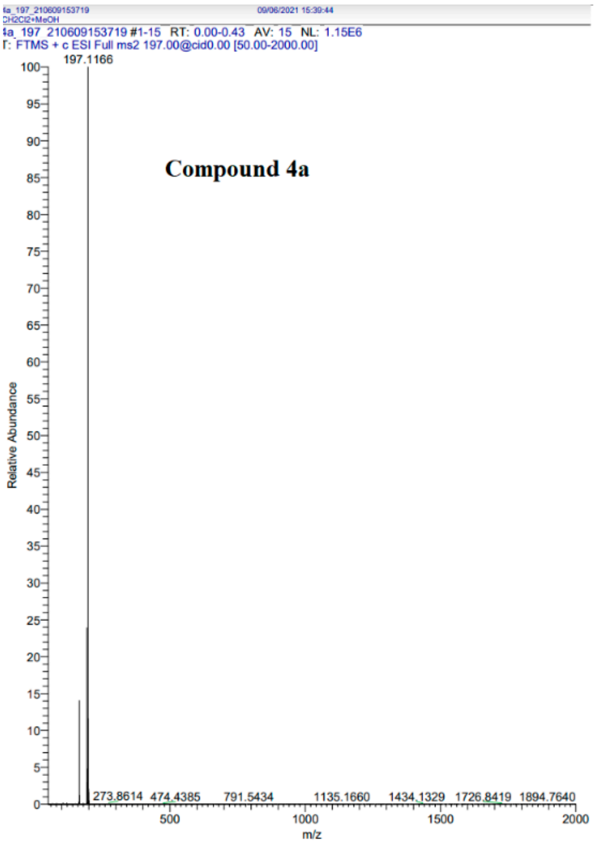

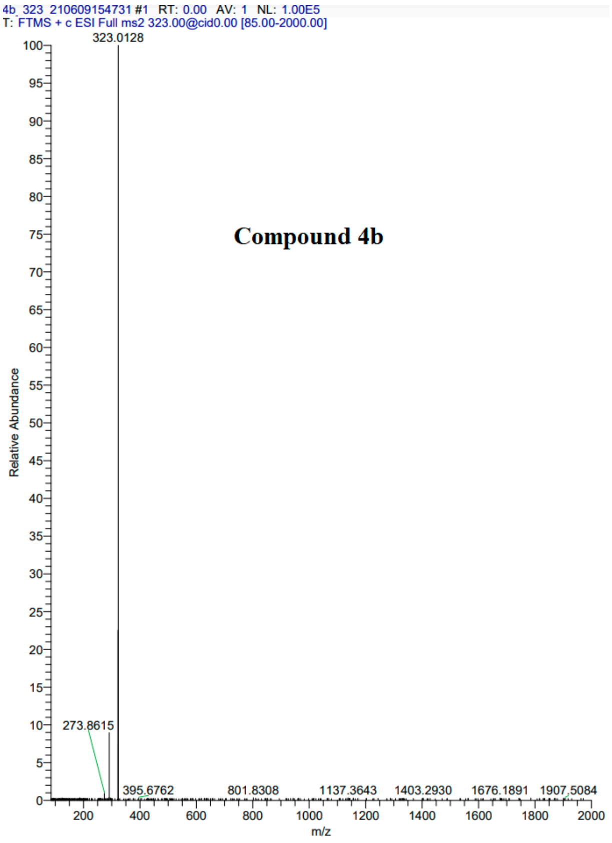

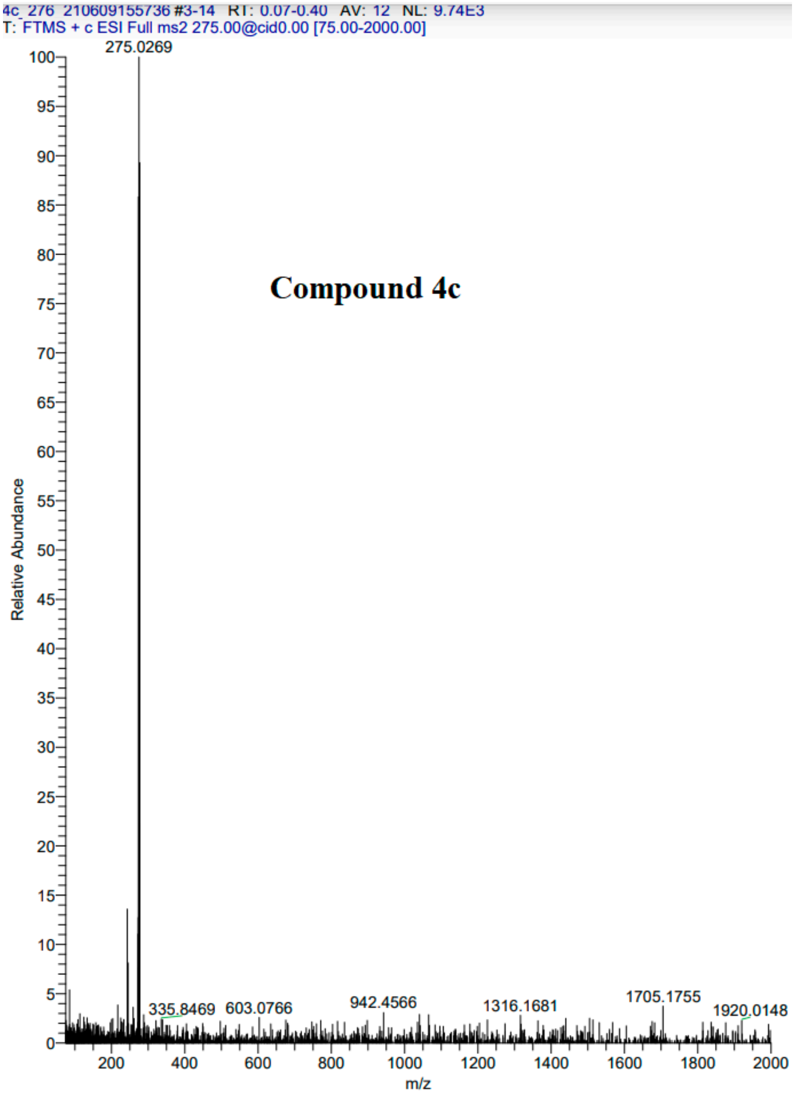
4c_276 210609155736 #3-14 RT: 0.07-0.40 AV: 12 NL: 9.74E3
T: FTMS + c ESI Full ms2 275.00@cid0.00 [75.00-2000.00]
275.0269
Compound 4c
Relative Abundance
335.8469
603.0766
942.4566
1316.1681
1705.1755
1920.0148
m/z

3.3. HR-MS spectrum for the compound **5b**.

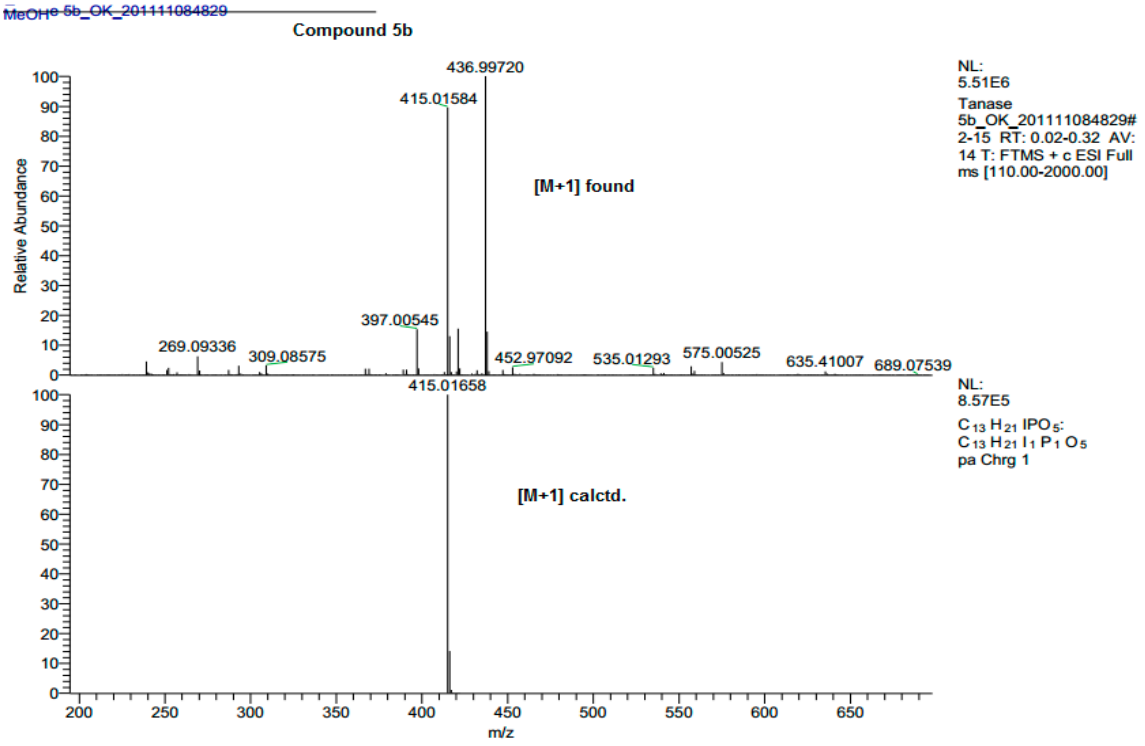

3.4. HR-MS spectrum for the compound **5c**.

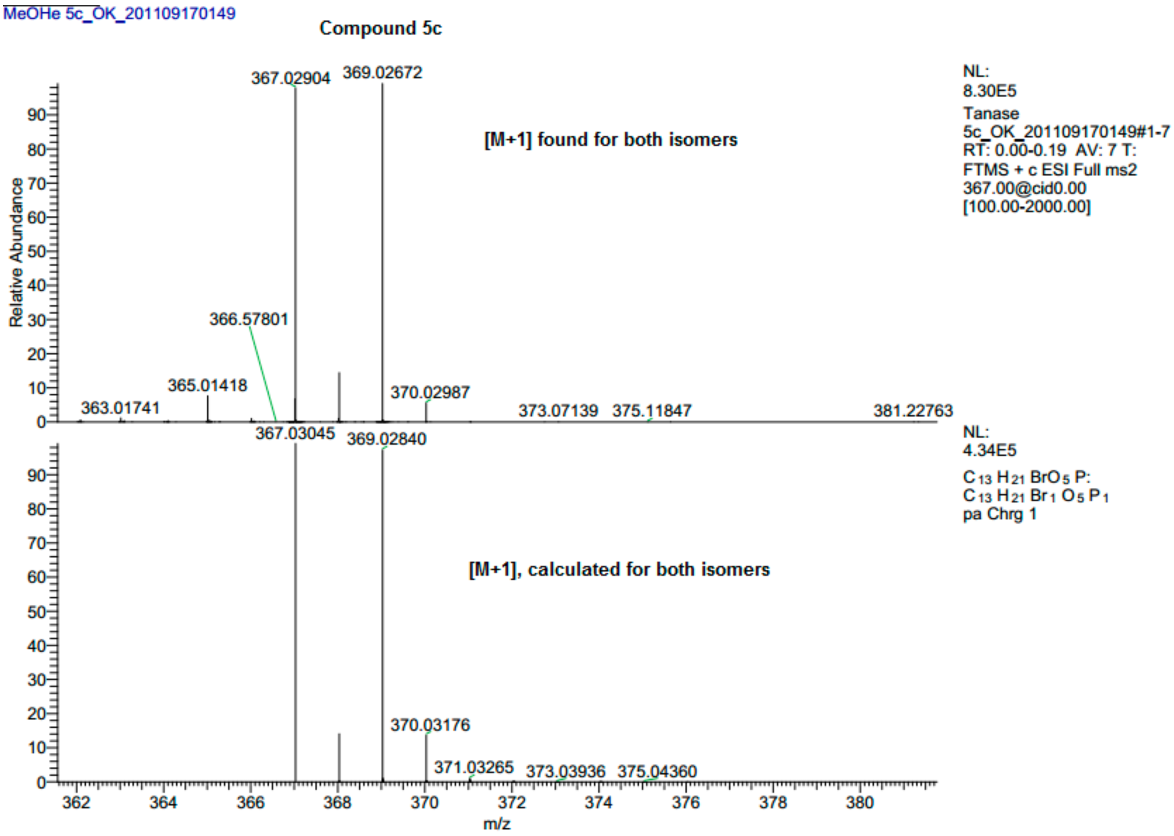

3.5. HR-MS spectrum for the compound **6b** and **6c**.

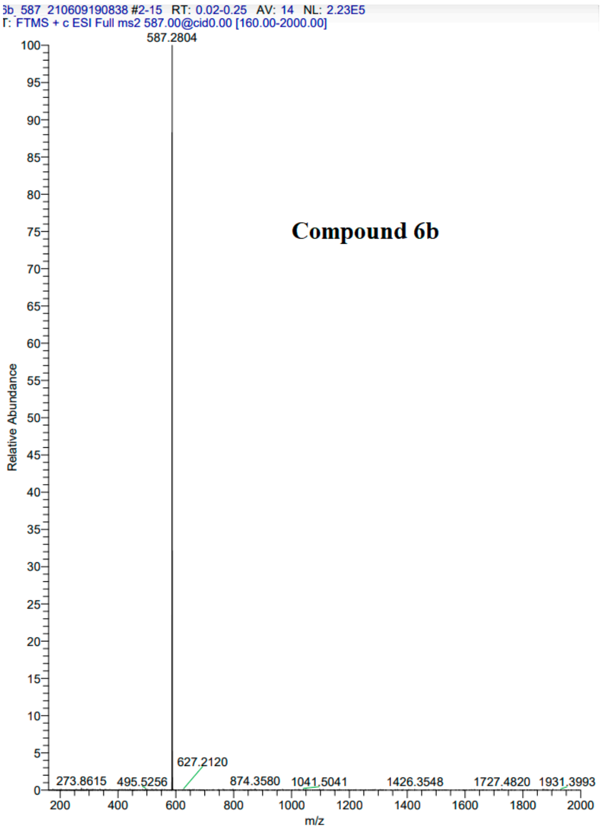

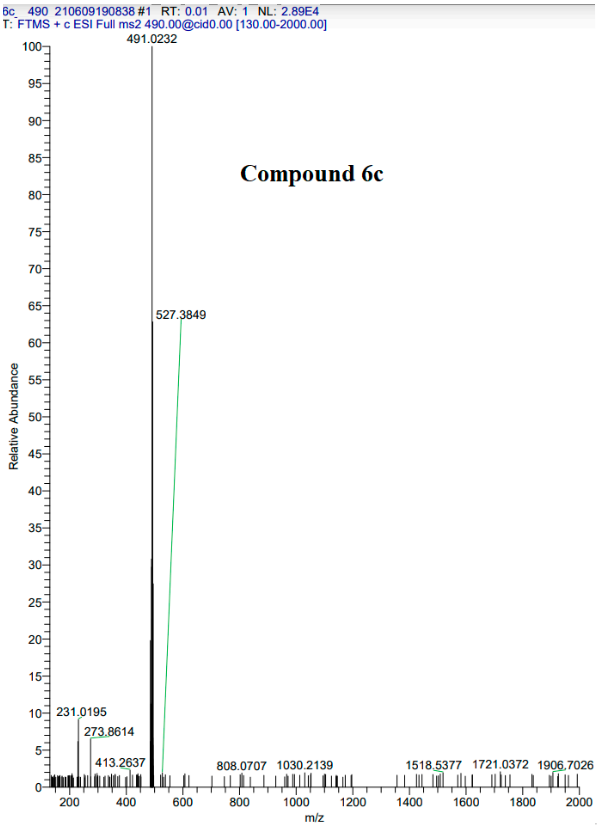

4. X-Ray crystallography data for compounds 6b and 6c.

Crystal structure determination of compound 6b from powder diffraction data

The X-Ray powder diffraction pattern was recorded with the monochromatic radiation (CuKα1 radiation) obtained using a germanium monochromator on a Brucker D8 Advance diffractometer equipped with a LYNXEYE detector (Bruker, Karlsruhe, Germany) and an operating tube at 40 kV, 40 mA. The sample was scanned in the range 2θ between 3 and 40^0 with a step of 0.01 and 3 s/step.
The procedure involved in solving the crystal structure from powder data consists in the following steps: X-ray pattern indexing to obtain the crystallographic system and the unit cell parameters, Pawley refinement and space group assignment, structural model search, and Rietveld refinement. The pattern indexing was done using Reflex module, implemented in Materials Studio software [1]. The following indexing programs were used: DICVOL96 [2] TREOR90 [3], and X-cell [4]. The solution found with all these programs, having the highest figure of merit (FOM) and all reflexions indexed, was: a=5.5124 Å, b=11.2583 Å, c=32.2033 Å, α=90º, β=94.527º, γ=90º, V=1992$Å^3$ and confirmed by Pawley refinement procedure (Rwp=4.26%). It has been established that compound **6b** belongs to $P2_1/n$ space group. Taking into account that the molecular weight of the compound is 584.23g/mol and the unit cell volume 1992.22 $Å^3$, the calculated density results as being ρ=1.95g/cm^3 if we consider that there are 4 molecules per unit cell. For the compound with Br the calculated density was 1.65 g/cm^3 which lead us to believe that the elementary cell and the space group are correctly determined. As a starting point for establishing the structural model, **6c** molecule was considered and the Br atom was replaced with Iodine and then the geometric optimization was done using the Castep code [5]. The structural model was determined by the direct-space Monte-Carlo method, using the parallel tempering procedure implemented in Powder Solve computing program [6]. The structural model obtained was refined by the Rietveld method which was performed with the Reflex module from Material Studio. We mention the most important parameters that have been refined: diffraction peaks, which were approximated as Pseudo-Voight function, (U, V, W) parameters from the Caglioti formula

[7], background profile approximated with a 20 polynomial of order, NA, NB profile parameters in Bragg-Brentano geometry, zero point shift parameter, (P1, P2, P3, P4) peaks asymmetry parameters from Berar-Baldinozzi approximation, (a*, b*, c* and R0) in March-Dollase correction for preferred orientation. The Table S1 (ESI) shows the refinement details for the compound **6c**, which were made on the basis of single crystal data (a); for the compound **6b**, the refinement details were obtained from powder diffraction data.

The agreement between the experimental diffraction pattern and the simulated one after the Rietveld refinement for sample **6b** is presented in Figure S1.

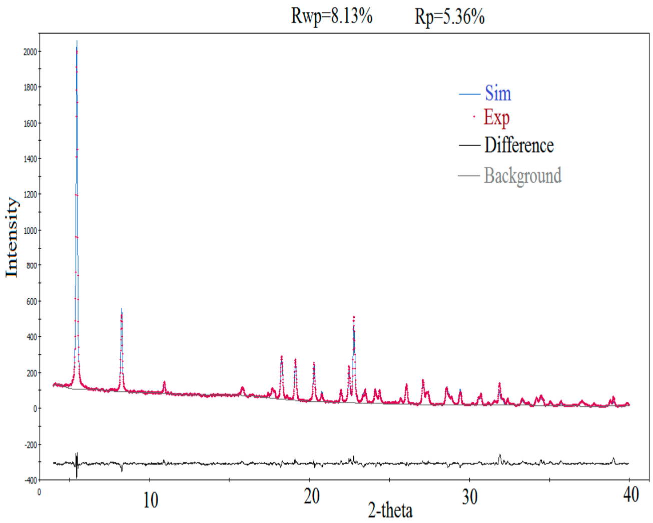

Figure S1. The fit between experimental and simulated **6b** diffraction powder after Rietveld refinement.

Table S1: X-ray refinement details from single crystal (a); and XRPD Rietveld refinement (b)

**(a)**		**(b)**	
Identification code	**6c**	Identification code	**6b**
Empirical formula	$C_{20}H_{26}Br_2O_4$	Empirical formula	$C_{20}H_{26}O_4I_2$
Formula weight	490.23	Formula weight	584.23
Temperature/K	293(2)	Temperature/K	293(2)
Crystal system	triclinic	Crystal system	monoclinic
Space group	P-1	Space group	$P2_1/n$ (14)
a/Å	8.1233(4)	a/Å	5.5121
b/Å	9.8197(3)	b/Å	11.2583
c/Å	12.7861(7)	c/Å	32.2033
α/°	97.873(4)	α/°	90
β/°	100.177(4)	β/°	94.5270
γ/°	95.683(4)	γ/°	90
Volume/Å^3	986.44(8)	Volume/Å^3	1992.22
Z	2	Z	4
ρ_{calc}g/cm^3	1.650	ρ_{calc}g/cm^3	1.950
μ/mm^{-1}	5.399	Radiation	CuKα1 (λ = 1.54056)
F(000)	496.0	2θ range for data collection/°	4 to 40

Crystal size/mm^3	0.35 × 0.08 × 0.07	R_{wp} (%)	8.13
Radiation	CuKα (λ = 1.54184)	R_p (%)	5.36
2Θ range for data collection/°	7.114 to 141.2		
Index ranges	-9 ≤ h ≤ 9, -7 ≤ k ≤ 11, -15 ≤ l ≤ 15		
Reflections collected	6285		
Independent reflections	3677 [R_{int} = 0.0230, R_{sigma} = 0.0276]		
Data/restraints/parameters	3677/1/235		
Goodness-of-fit on F^2	1.070		
Final R indexes [I>=2σ (I)]	R_1 = 0.0628, wR_2 = 0.1848		
Final R indexes [all data]	R_1 = 0.0667, wR_2 = 0.1910		
Largest diff. peak/hole / e Å^{-3}	2.46/-1.01		

Table S2. Distance between atoms (Å) for compounds **6c** and **6b**.

**6c**				**6b**			
Br1--C5	1.967	Br2-C5′	1.970	I1-C5	2.189	I2-C5′	2.104
C5- C4	1.512	C5′- C4′	1.514	C5- C4	1.576	C5′- C4′	1.552
C4- C3a	1.542	C4′- C3a′	1.549	C4 -C3a	1.576	C4′- C3a′	1.497
C3a-C6a	1.569	C3a′-C6a′	1.541	C3a-C6a	1.565	C3a′- C6a′	1.642
C6a -C6	1.531	C6a′- C6′	1.551	C6a- C6	1.456	C6a′- C6′	1.541
C6 - C5	1.511	C6′- C5′	1.502	C6- C5	1.577	C6′ - C5′	1.685
C6 - O7	1.436	C6′- O7′	1.442	C6- O7	1.445	C6′ - O7′	1.557
O7- C7	1.431	O7′- C7′	1.433	O7- C7	1.469	O7′ - C7′	1.407
C7- C1	1.521	C7′- C1′	1.516	C7- C1	1.467	C7′- C1′	1.652
C1- C6a	1.556	C1′- C6a′	1.543	C1- C6a	1.650	C1′ - C6a′	1.576
C1- C2	1.522	C1′- C2′	1.544	C1- C2	1.549	C1′- C2′	1.504
C2 - C3	1.524	C2′- C3′	1.527	C2- C3	1.607	C2′- C3′	1.564
C3- C3a	1.542	C3′- C3a′	1.540	C3- C3a	1.523	C3′- C3a′	1.583
C3- C8	1.502	C3′- C8′	1.496	C3- C8	1.352	C3′- C8′	1.437
C8- O1	1.177	C8′ - O2	1.430	C8- O1	1.232	C8′- O2	1.468

Table S3. Angles between atoms (˚) for compounds **6c** and **6b**.

**6c**			
C4-C5-C6	104.77	C4′-C5′-C6′	105.11
C5-C6-C6a	105.84	C5′-C6′-C6a′	106.36
C6-C6a-C3a	106.33	C6′-C6a′-C3a′	106.22
C6a-C3a-C4	105.09	C6a′-C3a′-C4′	105.11
C3a-C4-C5	103.78	C3a′-C4′-C5′	104.55

C6a-C6-O7	105.82	C6a′-C6′-O7′	106.36
C6-O7-C7	106.48	C6′-O7′-C7′	106.27
O7-C7-C1	105.15	O7′-C7′-C1′	105.19
C7-C1-C6a	102.22	C7′-C1′-C6a′	102.91
C1-C6a-C6	105.00	C1′-C6a′-C6′	104.73
C3-C3a-C6a	103.99	C3′-C3a′-C6a′	103.69
C3a-C6a-C1	106.29	C3a′-C6a′-C1′	107.08
C6a-C1-C2	106.10	C6a′-C1′-C2′	105.47
C1-C2-C3	104.57	C1′-C2′-C3′	103.71
C2-C3-C3a	104.00	C2′-C3′-C3a′	103.57
C3-C8-O1	126.08	C3′-C8′-O2	107.29
C3-C8-O2	110.11	C8′-O2-C8	117.26

**6b**			
C4-C5-C6	107.90	C4′-C5′-C6′	97.62
C5-C6-C6a	103.44	C5′-C6′-C6a′	109.20
C6-C6a-C3a	112.64	C6′-C6a′-C3a′	102.64
C6a-C3a-C4	105.24	C6a′-C3a′-C4′	106.37
C3a-C4-C5	102.04	C3a′-C4′-C5′	107.98
C6a-C6-O7	104.21	C6a′-C6′-O7′	102.91
C6-O7-C7	104.43	C6′-O7′-C7′	105.30
O7-C7-C1	106.34	O7′-C7′-C1′	107..07
O7-C1-C6a	98.71	O7′-C1′-C6a′	97.58
C1-C6A-C6	106.88	C1′-C6a′-C6′	110.54
C3-C3a-C6a	111.36	C3′-C3a′-C6a′	101.33
C3a-C6a-C1	103.16	C3a′-C6a′-C1′	104.44
C6a-C1-C2	103.71	C6a′-C1′-C2′	109.32
C1-C2-C3	108.69	C1′-C2′-C3′	105.11
C2-C3-C3a	98.62	C2′-C3′-C3a′	104.71
C3-C8-O1	132.38	C3′-C8′-O2	115.39
C3-C8-O2	113.32	C8′-O2-C8	124.81

References

1. *Dassault SystemesBiovia, [Materials Studio]*; v8.0.0.843; Dassault Systemes: San Diego, CA, USA, 2014
2. Boultif, A.; Louer, D. Powder pattern indexing with the dichotomy method. *J. Appl. Crystallogr.* **2004**, *37*, 724–731.
3. Werner, P.-E.; Eriksson, L.; Westdahl, M. TREOR, a semi-exhaustive trial-and-error powder indexing program for all symmetries *J. Appl. Crystallogr.* **1985**, *18*, 367–370.
4. Neumann, M.A. *X-Cell*: A novel indexing algorithm for routine tasks and difficult. *J. Appl. Crystallogr.* **2003**, *36*, 356–365, doi:10.1107/S0021889802023348.
5. Clark, S.J.; Segall, M.D.; Pickard, C.J.; Hasnip, P.J.; Probert, M.J.; Refson, K.; Payne, M.C. First principles methods using CASTEP. *Z. für Krist.* **2005**, *220*, 567–570, doi:10.1524/zkri.220.5.567.65075.
6. Engel, G.E.; Wilke, S.; König, O.; Harris, K.D.M.; Leusen, F.J. J. PowderSolve—A complete package for crystal structure solution from powder diffraction patterns. *J. Appl. Cryst.* **1999**, *32*, 1169–1179.

7. Caglioti, G.; Paoletti, A.; Ricci, E.P. Choice of collimators for a crystal spectrometer for neutron diffraction. *Nucl. Instrum.* **1958**, *3*, 223–228.
